# Systemic HER3 ligand-mimicking nanobioparticles enter the brain and reduce intracranial tumour growth

**DOI:** 10.1038/s41565-025-01867-7

**Published:** 2025-02-21

**Authors:** Felix Alonso-Valenteen, Simoun Mikhael, HongQiang Wang, Jessica Sims, Michael Taguiam, James Teh, Sam Sances, Michelle Wong, Tianxin Miao, Dustin Srinivas, Nelyda Gonzalez-Almeyda, Ryan H. Cho, Romny Sanchez, Kimngan Nguyenle, Erik Serrano, Briana Ondatje, Rebecca L. Benhaghnazar, Harry B. Gray, Zeev Gross, John Yu, Clive N. Svendsen, Ravinder Abrol, Lali K. Medina-Kauwe

**Affiliations:** 1 Department of Biomedical Sciences, Cedars-Sinai Medical Center, Los Angeles, CA, USA.; 2 Neurosurgical Institute, Cedars-Sinai Medical Center, Los Angeles, CA, USA.; 3 Board of Governors Regenerative Medicine Institute, Cedars-Sinai Medical Center, Los Angeles, CA, USA.; 4 California State University, Northridge, CA, USA.; 5 California Institute of Technology, Pasadena, CA, USA.; 6 Technion-Israel Institute, Haifa, Israel.; 7 University of California, Los Angeles, Los Angeles, CA, USA.

## Abstract

Crossing the blood–brain barrier (BBB) and reaching intracranial tumours is a clinical challenge for current targeted interventions including antibody-based therapies, contributing to poor patient outcomes. Increased cell surface density of human epidermal growth factor receptor 3 (HER3) is associated with a growing number of metastatic tumour types and is observed on tumour cells that acquire resistance to a growing number of clinical targeted therapies. Here we describe the evaluation of HER3-homing nanobiological particles (nanobioparticles (NBPs)) on such tumours in preclinical models and our discovery that systemic NBPs could be found in the brain even in the absence of such tumours. Our subsequent studies described here show that HER3 is prominently associated with both mouse and human brain endothelium and with extravasation of systemic NBPs in mice and in human-derived BBB chips in contrast to non-targeted agents. In mice, systemically delivered NBPs carrying tumoricidal agents reduced the growth of intracranial triple-negative breast cancer cells, which also express HER3, with improved therapeutic profile compared to current therapies and compared to agents using traditional BBB transport routes. As HER3 associates with a growing number of metastatic tumours, the NBPs described here may offer targeted efficacy especially when such tumours localize to the brain.

The blood–brain barrier (BBB) prevents most systemic therapeutics from entering the brain parenchyma and reaching intracerebral tumours, contributing to poor prognoses for patients^1^. Clinical options are limited to surgery, chemotherapy and radiation therapy; however, disseminated tumours pose challenges for surgical removal, and untargeted drugs reaching such tumours also affect healthy tissue^2^. Targeted antibody therapies such as the HER2/ErbB2 monoclonal antibody trastuzumab (Tz, Herceptin) poorly extravasate in the brain (requiring BBB opening methods)^3,4^ thus poorly affecting brain metastases even when peripheral tumours respond to Tz treatment^5^. The assumption that brain metastasis compromises the BBB is challenged by the inability of systemic treatments to access the brain parenchyma at sufficiently therapeutic levels^6,7^, suggesting that a barrier persists even with a tumour present. Worse yet, Tz treatment is associated with increased brain metastases in breast cancer patients^5,8^. Therapeutic options are further limited for triple-negative breast cancer (TNBC), which lacks HER2 amplification and is highly brain metastatic with poor clinical outcomes^9,10^. Patients with breast cancer brain metastases typically survive less than one year^11^ with options limited to chemotherapy that can effectuate broad off-target toxicity^12,13^, hence improved alternatives are needed. However, transferring systemic targeted therapies across the BBB and into brain-localized tumours remains a major challenge.

Increased cell surface density of HER3/ErbB3 is associated with a growing number of metastatic, resistant and brain-localized tumours, including prostate, gastric, colon, lung, pancreatic, head and neck, ovarian, cervical, glioblastoma, melanoma and breast cancers^14–17^, especially brain-metastatic HER2+ and triple-negative breast tumours^18,19^. HER3 and HER2 belong to the ErbB growth factor receptor kinase family but unlike HER2, a non-functional kinase domain^20^ makes HER3 a poor target for kinase inhibitors. Nevertheless, several HER3-targeted antibodies are being tested in clinical trials for peripheral tumours^15,21^ but may be limited by the resistance of HER3+ tumours to signal-blocking therapies^15^ while the BBB limits antibody access to intracerebral tumours^22–24^.

Here we demonstrate that a bioengineered tumour-invading protein, HPK, can form cargo-encapsulating nanosized biologically based particles (nanobioparticles (NBPs)) that penetrate resistant and metastatic tumours including TNBC. Unlike antibodies, HPK bears a HER3-homing function derived from the natural HER3 ligand neuregulin to mediate tumour-targeting and induce endocytic uptake. HPK also utilizes the capsid-forming and endosomal-disrupting functions of an adenovirus-derived capsid protein to encapsulate and deposit macromolecular and other membrane-impermeable cargo into HER3-expressing tumour cells.

Evaluating preclinical biodistribution revealed that the NBPs entered the brain and we subsequently found that HER3 is prominently associated with both mouse and human brain vasculature and with systemic NBP transfer into the brain parenchyma. These findings corroborate studies showing that antibodies blocking HER3 prevented neuregulin entry into the mouse brain parenchyma^25^, suggesting that HER3 facilitates BBB transcytosis of circulating neuregulins. As neuregulins facilitate neuronal maturation, myelination, and repair^26^, a route mediating systemic neuregulin passage into the brain could be engaged by NBPs. Hence, this study leveraged an induced pluripotent stem cell (iPSC)-derived organ-on-chip, or BBB chip, to interrogate the BBB passage mechanism of NBPs in a human-derived system. The association of HER3 with brain metastasis^21^ led us to test whether HER3 can transport systemic NBPs across the BBB and into intracerebral TNBC tumours and reduce tumour growth when delivering tumoricidal agents compared with currently used clinical therapy and traditional BBB crossing agents.

Studies interrogating nanocarrier interactions with biological barriers can benefit nanomedicine development^27^. Accordingly, these studies have used clinically relevant technologies to identify a new route for systemic macromolecular delivery into the brain with possible advantages over traditional BBB transporters, offering NBPs as an improved targeting option for overcoming therapeutic barriers especially when tumours localize to the brain.

## NBPs stably assemble and reduce growth of peripheral TNBC

**Fig. 1 |:**
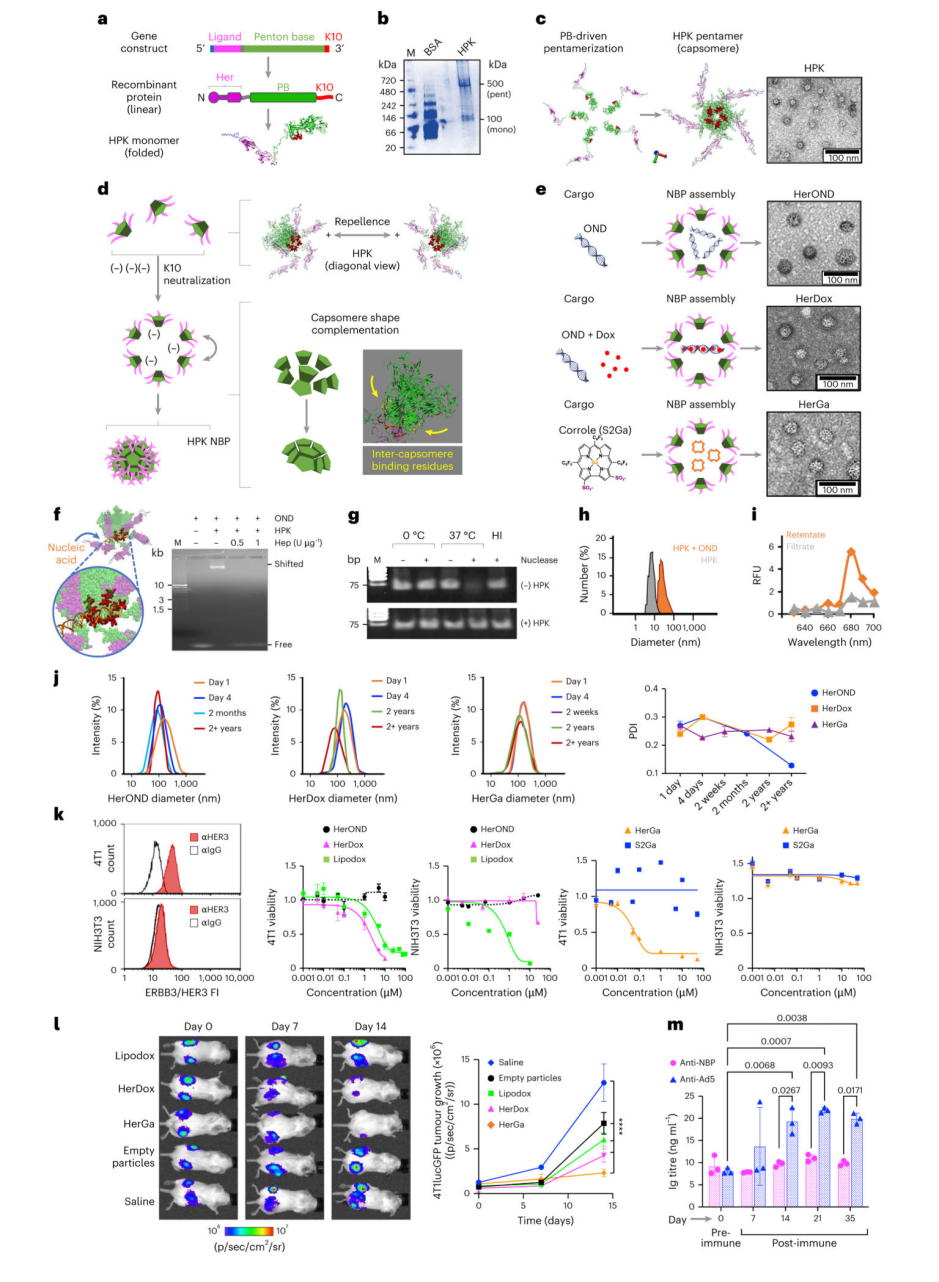
NBP assembly and characterization. **a**, Domain map of the HPK gene and protein linear sequence, and a ribbon model highlighting the HER3-binding motif (Her), penton base (PB) and decalysine (K10). **b**, Native PAGE of purified HPK delineating electrophoretic species matching pentamers (pent) and monomers (mono). M, molecular size marker. **c**, Structural modelling (left) and TEM (right) of HPK capsomeres. **d**, Putative mechanism of NBP self-assembly depicting charge-repelling capsomeres in solution that converge upon charge neutralization by anionic cargo leading to inter-capsomere binding and shape complementation to form a polyhedron. **e**, HPK assembly with indicated cargo forming specified NBPs. Right: TEM of NBPs. **f**, Capsomere binding to nucleic acid cargo. Structural model shows the HPK capsomere interacting with nucleic acid through the K10 domains. Right: electrophoretic mobility shift assay of OND alone (Free) and with HPK (Shifted) ± heparin (Hep). **g**, Gel electrophoresis and ethidium bromide staining of OND ± incubation in serum nucleases ± preassembly with HPK. HI, heat-inactivated serum. **h**, Hydrodynamic diameters of HerOND (HPK + OND) compared with HPK alone. **i**, Fluorimetry of NBP containing NIR-labelled OND after ultrafiltration to isolate assembled particles (retentate) from unassembled cargo (filtrate). RFU, relative fluorescence units. **j**, Hydrodynamic diameters and polydispersion indices (PDIs) of indicated NBPs (HerOND, HerDox, HerGa) after −20 °C storage for various time periods. **k**, Cell surface HER3 and killing curves (showing cell survival at 24 h after treatment) on mouse TNBC (4T1) and fibroblast (NIH3T3) lines. Data are presented as mean ± s.d. (*n* = 3). FI, fluorescence intensity. **l**, BLI and growth of subcutaneous bilateral 4T1 tumours in 8-week-old female BALB/c mice during systemic treatment with indicated reagents. Day 0, first day of treatment (5 days after tumour implantation). ****P = 1.8 × 10^−7^ (saline versus empty particles); *P* = 1.1 × 10^−10^ (saline versus lipodox); *P* = 2.9 × 10^−13^ (saline versus HerDox); *P* = 5.1 × 10^−14^ (saline versus HerGa). Significances determined at 95% confidence interval (CI) by two-way multiple comparisons ANOVA and Tukey post hoc test (*n* = 5 per treatment group). Data are presented as mean ± s.d. **m**, Ig titres in sera of tumour-free BALB/c mice inoculated with empty NBPs (at doses equating to 0.2 mg kg^−1^ HerDox) twice per week for 4 weeks. Statistical significances determined using two-way multiple-comparisons ANOVA (95% CI) and Tukey post hoc test (*n* = 3 mice per treatment group). Data are presented as mean ± s.d.

Non-covalent capture of therapeutic molecules in tumour-targeted nanocapsules can avoid chemical modification that may alter function and therapeutic potency but requires stable encapsulation for effective in vivo delivery. Membrane-impermeable therapeutics also require robust penetration of tumour barriers to access intracellular targets. With these needs in mind, the membrane-penetrating penton base protein, a self-assembled homopentameric ring (or capsomere)^28^ capping each vertex of the icosahedral adenovirus capsid^29,30^, provides an attractive foundation for the recombinant protein HPK which we designed to encapsulate anionic cargo for missile-like delivery to aggressive tumours. HPK contains the receptor-binding region of the HER3 ligand neuregulin-1α^31^ produced as an N-terminal fusion to a penton base protein containing a C-terminal decalysine tail (Fig. 1a).

HPK forms high-molecular-weight electrophoretic species consistent with pentamers under native conditions (Fig. 1b), echoing the ability of recombinant soluble (virus-free) penton base protein to self-pentamerize^30,32^. Computational modelling suggests that the penton base domain of HPK drives the formation of oligomeric barrels consistent with ring-like capsomeres seen under transmission electron microscopy (TEM) (Fig. 1c) Structural modelling shows that homo-oligomerization aligns the decalysines on one end of the capsomere barrel (Supplementary Fig. S1), creating a positively charged surface causing inter-capsomere repellence while attracting anionic cargo (Fig. 1d). Electrostatic binding to anionic cargo should neutralize HPK charge repellence and allow the capsomeres to converge around the cargo, bringing into contact inter-capsomere binding motifs^32^ driving shape complementation (Supplementary Fig. 1). The cork-shaped structure of the penton base domain imparts a natural curvature, leading to a fully formed spherical shell whose assembly may be nucleated by the cargo. In agreement, exposing HPK to anionic cargo under physiological conditions triggers formation of polyhedra or NBPs (Fig. 1e). These NBPs share similar diameter and morphology whether the cargo comprises small nucleic acids such as oligonucleotide duplexes (ONDs), ONDs intercalated with the chemotherapy drug doxorubicin (Dox), or tumour-toxic sulfonated corrole molecules (S2Ga), producing NBPs designated HerOND, HerDox and HerGa, respectively (Fig. 1e).

Studying HerOND assembly demonstrates that the model in Fig. 1d supports the structures shown in Fig. 1e. Specifically, HPK capsomeres slow the electrophoretic migration of OND, indicative of protein binding to the nucleic acid, whereas the highly anionic molecule, heparin, disrupts this interaction (Fig. 1f), suggesting that electrostatic attraction mediates cargo binding. This interaction prevents serum-nuclease-mediated degradation of nucleic acid cargo (Fig. 1g and Supplementary Fig. 2), suggesting that HPK encapsulates the cargo. This is supported by the increase in hydrodynamic diameter when HPK encounters OND versus HPK alone (Fig. 1h) and by the retention of fluorescently tagged ONDs on ultrafiltration membranes when incubated with HPK in contrast to free OND (Fig. 1I).

The scrambled 30 bp OND used to assemble HerOND is functionally inert while serving as a capture device for intercalating Dox to assemble HerDox, whereas HerGa forms through direct binding with HPK. The minimum cargo concentrations showing maximum binding to HPK (Supplementary Figs. 3 and 4) provided the stoichiometric ratios used for assembling NBPs, which retained their general stoichiometries at 5× and 10× scalable preparations (Extended Data Table 1). All three NBPs exhibited negative zeta potentials exceeding −15 mV and negative mobility values (Supplementary Fig. 5 and Extended Data Table 1), low polydispersity and cargo retention upon prolonged incubation and storage (Supplementary Figs. 6 and 7), and long-term (≥1 year) stability at 4 °C, −20 °C and −80 °C (Supplementary Fig. 8) with no notable change in hydrodynamic profile even after several years in frozen storage (Fig. 1j).

To evaluate their in vitro drug-delivery capacity, we exposed each NBP to HER3+ tumour cells (mouse TNBC 4T1) and HER3− non-tumour cells (mouse fibroblast NIH3T3) in culture and compared each to untargeted and drug-free controls. HER3 positivity was determined by specific immunoreactivity (Fig. 1k and Supplementary Fig. 9) above most non-tumour peripheral tissue (Supplementary Figs. 10–12). HerOND used as a drug-free NBP showed no notable cytotoxicity on tumour and non-tumour cells, whereas HerDox and HerGa showed preferential cytotoxicity on the HER3+ tumour cells in contrast to the HER3− fibroblasts (Fig. 1k and Extended Data Table 1). These findings compared favourably against S2Ga alone, which lacks cell membrane permeability^33^, and against the non-targeted clinically used chemotherapy agent liposomal doxorubicin (lipodox or Doxil), which serves here as a benchmark for therapeutic efficacy but showed toxicity on both HER3+ tumour cells and HER3− fibroblasts (Fig. 1k). HerDox also compared favourably against lipodox and empty particles on HER3+ human lines derived from TNBC, glioblastoma and malignant melanoma (whose high HER3/ErbB3 expression echo clinical specimens of malignant and metastatic melanoma, several types of brain tumours, invasive breast carcinoma and TNBC; Supplementary Figs. 10–13), bone-metastatic prostate cancer, ovarian (including chemoresistant) cancer, and Herceptin-resistant HER2+ breast cancer especially compared with Herceptin (Tz; Supplementary Fig. 14). Systemic HerDox biodistribution in mice with HER3+ tumour xenografts yielded tumour-preferential accumulation and retention while clearing from liver and kidney tissue within 24 h (Supplementary Fig. 15)^34^. Systemic HerDox and HerGa compared favourably with lipodox in an immunocompetent orthotopic mouse TNBC model receiving circulating drug dosages equating 0.2 mg kg^−1^ Dox or S2Ga (determination of dosage and regimen is described in the Methods; Fig. 1l and Supplementary Figs. 16 and 17). Drug-lacking NBPs (HerOND or empty particles) showed no growth-promoting effect on tumour cells in vitro (Supplementary Fig. 14 and Fig. 1k) and in vivo (Fig. 1l) while lacking significant induction of neutralizing antibodies (Fig. 1m) and proinflammatory cytokines (Supplementary Fig. 18).

## NBPs bind and enter tumour cells through HER3

**Fig. 2 |:**
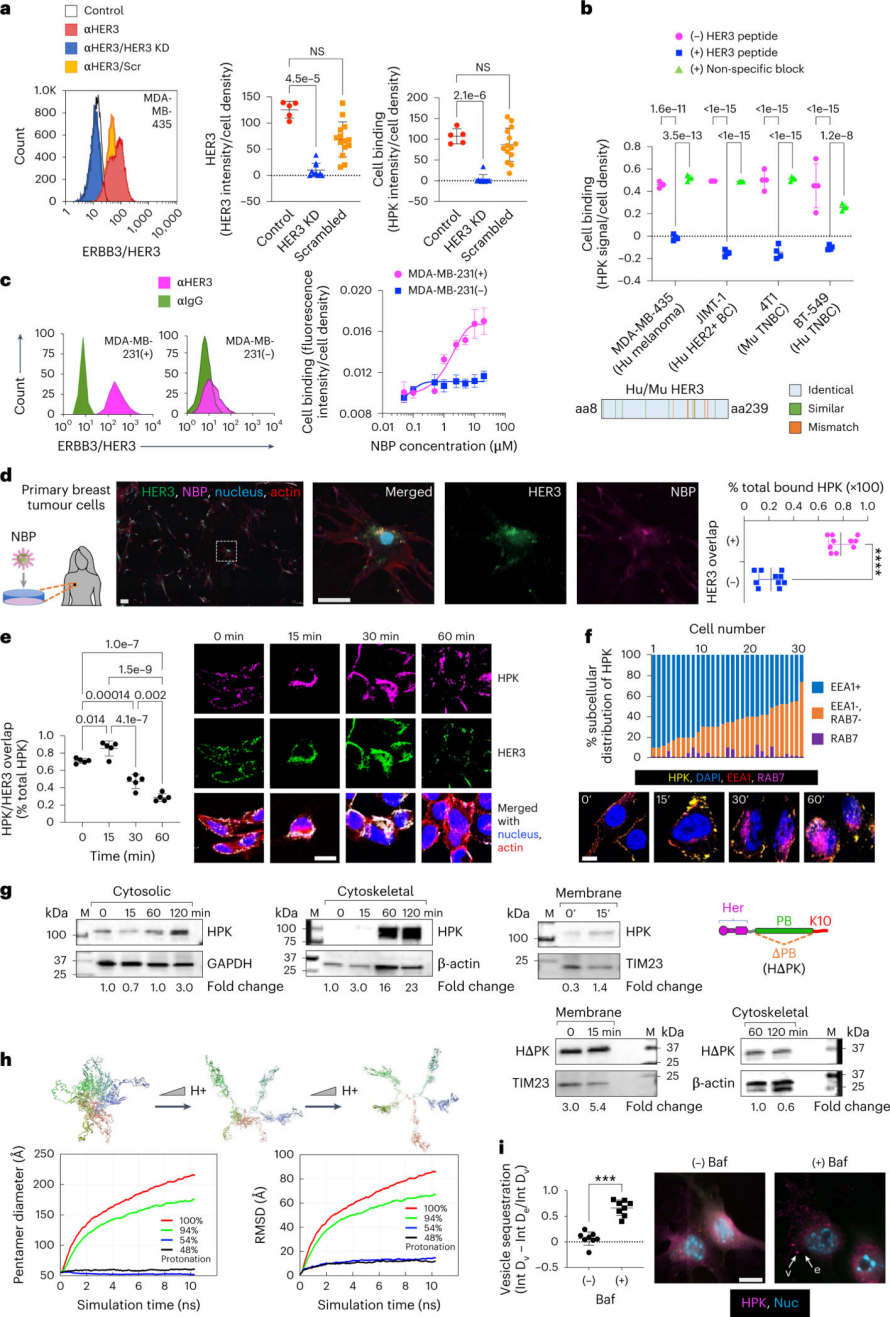
HER3 expression and HPK penetration of tumour cells. **a**, NBP binding on human HER3+ tumour cells ± HER3 silencing or treatment with scrambled (Scr) siRNA 24 h previously (HER3, *n* = 10; Scr, *n* = 14; independent fields per cell population). KD, knock-down. Control, non-transfected cells (*n* = 5 independent fields per cell population). Statistical significances determined using one-way multiple-comparisons ANOVA (95% CI) and Dunnett post hoc test. Data are presented as mean ± s.d. NS, not significant. **b**, Particle binding to indicated cells ± competing HER3 peptide. Statistical significances determined using two-way multiple comparisons ANOVA (95% CI) and Tukey post hoc test, *n* = 4 biological replicates. Data are presented as mean ± s.d. Inset: graphical alignment of mouse and human HER3 ligand-binding domains. **c**, NBP (HerOND delivering fluorescently tagged OND) binding on human MDA-MB-231 TNBC sublines displaying high (+) and low (−) cell surface HER3. *n* = 3 biological replicates. Data are presented as mean ± s.d. **d**, HerOND binding to patient-derived tumour cells. The delineated region is enlarged in the right three panels. Graph shows quantification of NBP overlapped with HER3 versus non-HER3 overlapped NBP. ****P = 1.4 × 10^−10^ (95% CI) using two-tailed unpaired *t*-test, *n* = 10. Data are presented as mean ± s.d. Scale bars, 25 μm. **e**, HER3 + MDA-MB-435 human tumour cells imaged and quantified at indicated time points for HPK particle uptake and intracellular trafficking. Blue, nucleus; red, actin; green, HER3; magenta, HPK. Scale bar, 8 μm. Statistical significances determined using one-way multiple-comparisons ANOVA (95% CI) and Tukey post hoc test, *n* = 5. Data are presented as mean ± s.d. **f**, HPK particle trafficking (immunocytofluorescence) and intracellular distribution (graph) in HER3 + MDA-MB-435 cells relative to early (EEA1) and late (RAB7) endo/lysosomes. Scale bar, 5 μm. **g**, Immunoblots of HER3 + MDA-MB-435 subcellular fractions during HPK or HΔPK uptake. Quantified uptake levels were normalized against respective cell fraction loading controls. Fold change is relative to 0 min. **h**, Snapshots of MD-simulated tHPK pentamer at decreasing pH. Designated colours distinguish each monomer. RMSD, root mean square deviation. **i**, Immunocytofluorescence of 4T1 cells at 30 min capsomere uptake ± bafilomycin-A1 (Baf). Scale bar 8 μm. Graph, vesicular (v) and extravesicular (e) intensities. ***P = 0.0002 (95% CI) using two-tailed unpaired Mann–Whitney test, *n* = 8. Data are presented as mean ± s.d.

We used HerOND to validate the receptor specificity and intracellular trafficking of NBPs while avoiding cytotoxic impact. The human melanoma-like cell line, MDA-MB-435, displays high cell surface HER3 and concomitant NBP binding density that was reduced by silencing HER3 expression (Fig. 2a), suggesting that NBP cell binding is directed by HER3. In agreement, blocking the HER3 ligand (Supplementary Fig. S19) significantly reduced binding to both human (Hu) and mouse (Mu) HER3+ tumour cell lines and highlights the species cross-reactivity of HPK which is supported by the high amino acid sequence identity between human and mouse HER3 (Fig. 2b)^35^. Sublines of the human MDA-MB-231 TNBC cell line isolated for displaying contrasting HER3 densities showed corresponding NBP binding densities (Fig. 2c). HER2+ patient-derived primary tumour cells displayed marked HER3 density and HER3-localized binding by HerOND (Fig. 2d).

Cell binding on HER3 + MDA-MB-435 cells induced robust uptake coincident with HER3 during the early stages of internalization (0–30 min after uptake), whereas HER3 and NBP showed less overlap at later stages (1 h after uptake) with the HER3 density significantly decreasing by 1 h (Fig. 2e). The intracellular localization of NBP remained excluded from the lysosomal biomarker Rab7 (Fig. 2f) and distributed to cytosolic and cytoskeletal compartments (Fig. 2g) consistent with penton base endosomal escape and postendosomal transit along microtubules toward the nucleus^30,36^. Compared with HPK, a penton-base-deleted construct, HΔPK, displayed early sequestration in the membrane compartment and considerably reduced arrival at late (cytoskeletal) trafficking compartments (Fig. 2g and Supplementary Fig. 20), indicating that postendosomal delivery requires the penton base domain.

The protonation of solvent-accessible residues lining the lumen of the HPK capsomere barrel (Supplementary Fig. 21) at low pH may mediate the endosomolytic mechanism. Molecular dynamics (MD) computational simulation of HPK pentamers in acidic conditions predicts that the monomers disperse (Fig. 2h and Supplementary Information), exposing the hydrophobic intrapentamerization domains which could interact with endosomal membrane lipids, leading to membrane destabilization. In agreement, the vesicular proton pump inhibitor bafilomycin sequestered HPK in vesicle-like puncta after uptake and prevented HPK diffusion into the cytoplasm of HER3 + 4T1 TNBC cells (Fig. 2i), suggesting that endosomal penetration is pH-dependent.

## Systemic NBPs exhibit tumour and brain localization in mice

**Fig. 3 |:**
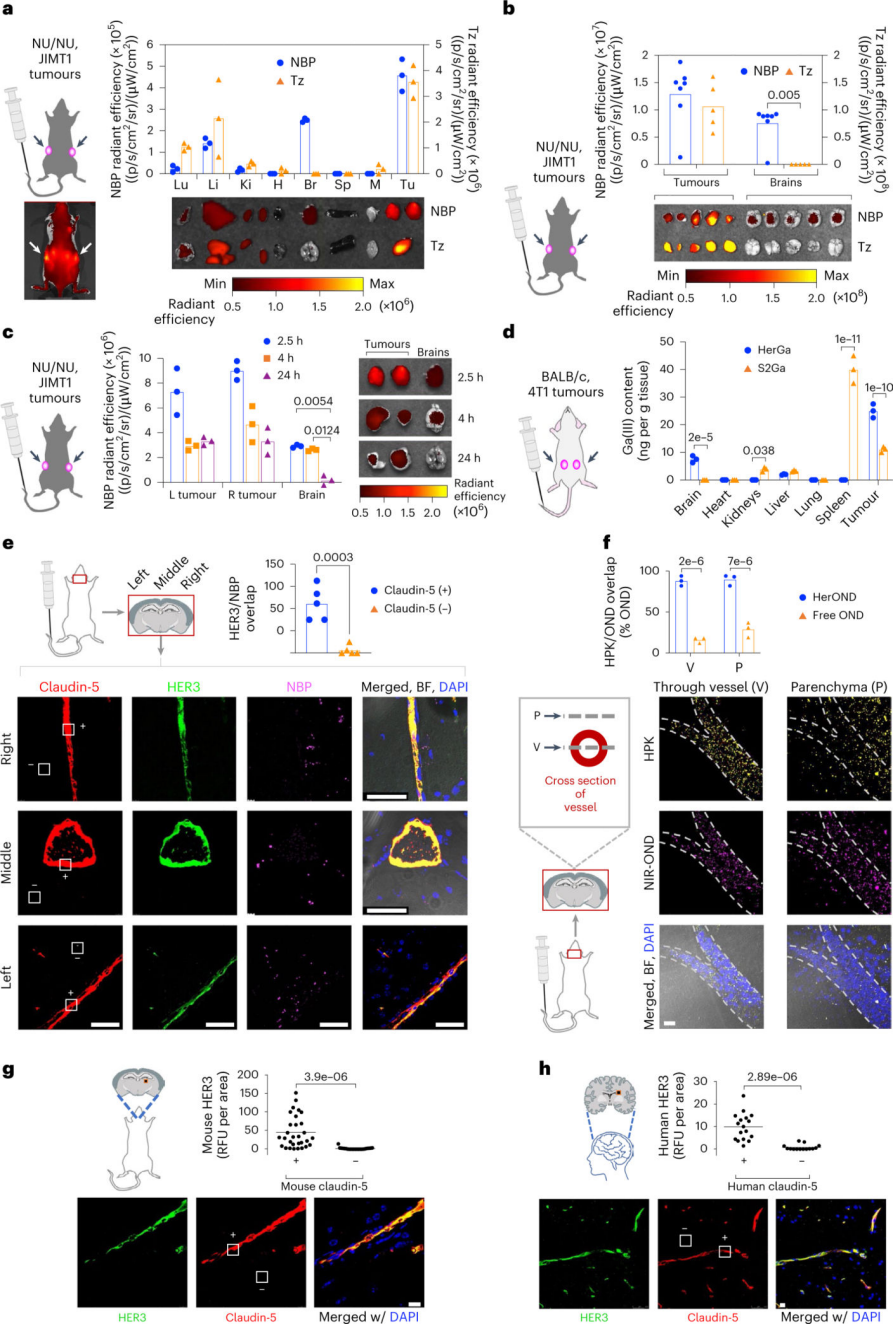
Systemic NBP accumulation in mouse brain. **a**, Tissue distribution of NIR-labelled NBPs or Tz at 4 h after systemic delivery in 8-week-old female NU/NU mice (*n* = 5 per treatment) bearing subcutaneous JIMT-1 breast tumours. Arrows, tumour locations. *x* axis: Lu, lung; Li, liver; Ki, kidney; H, heart; B, brain; S, spleen; M, muscle; Tu, tumour. Data are presented as mean ± s.d. **b**, Brain and tumour distribution of NIR-labelled HPK or Tz at 2.5 h after systemic delivery in 8-week-old female NU/NU mice (*n* = 5 per treatment) bearing subcutaneous JIMT-1 breast tumours. Statistical significance determined using two-tailed unpaired *t*-test (95% CI). Data are presented as mean ± s.d. **c**, Tumour and brain distribution at indicated time points after systemic administration of NBPs delivering NIR-OND in 8-week-old female NU/NU mice (*n* = 6 per treatment) bearing subcutaneous JIMT-1 tumours. Statistical significances determined using two-way multiple-comparisons ANOVA (95% CI) and Tukey post hoc test. **d**, Tissue content of gallium(III)-metallated corrole (S2Ga) measured by ICP-MS at 6 h after systemic delivery alone or by NBPs in 8-week-old female BALB/c mice (*n* = 6 per treatment) bearing orthotopic 4T1 tumours. Statistical significance determined using two-way multiple-comparisons ANOVA (95% CI) and Šídák post hoc test. Data are presented as mean ± s.d. **e** NBP and HER3 localization at claudin-5-positive (+) and claudin-5-negative (−) areas in the brain at 2.5 h after systemic delivery in 8-week-old female BALB/c mice (*n* = 5 per treatment). Statistical significance determined using two-tailed unpaired *t*-test (95% CI). Data are presented as mean ± s.d. Scale bars, 50 μm. **f**, HPK and NIR-OND overlap analysis from brain specimens (*n* = 3) in **e**, showing adjacent *z*-axis planes transecting the vessel (V) and overlying parenchyma (P). Free OND, NIR-OND without overlapping HPK. Statistical significances determined using two-way multiple-comparisons ANOVA (95% CI) and Šídák post hoc test. Data are presented as mean ± s.d. Scale bar, 20 μm. **g**,**h**, Immunohistofluorescence of frontal cortex from non-diseased adult murine (**g**) and human (**h**) brains showing HER3 overlap with blood vessels within brain specimens. White squares delineate representative claudin-5-positive (+) and claudin-5-negative (−) areas. Data points represent the means of individual regions of interest (mouse, *n* = 29; human, *n* = 17) in each indicated zone. Statistical significances determined using two-tailed unpaired *t*-test (95% CI). Scale bars, 20 μm.

We compared HerOND with the HER2 antibody Tz for in vivo tumour-homing capacity after systemic delivery in mice bearing peripheral HER2 + JIMT-1 human breast tumours which display both HER2 and HER3 (Supplementary Fig. 14) and inherently resist Tz^37^. HerOND delivering near-infrared fluorescence (NIR)-labelled oligonucleotides exhibited tumour-preferential accumulation and unexpected detection in the brain in contrast with NIR-labelled Tz which accumulated in HER2+ tumours but not in the brain (Fig. 3a,b). Systemic HerOND was detected in tumours and brains at 2.5 and 4 h after administration and remained detectable in tumours but not in brains at 24 h (Fig. 3c). Inductively coupled plasma mass spectrometry (ICP-MS) of immunocompetent mouse tissue lysates after systemic delivery of HerGa validated these findings (Fig. 3d) and showed that gallium(III) metal (which is rarely present endogenously) was highest in the brain and syngeneic TNBC tumours after HPK-mediated delivery but undetectable in brains after S2Ga delivery and significantly lower than HerGa in tumours. Systemic HerOND also exhibited brain accumulation in tumour-free immunocompetent mice in contrast to labelled Tz and non-targeted protein (BSA) (Supplementary Fig. 22), and remained detectable in the brains of tumour-free mice up to 4 h after administration after which the NIR signal cleared from brains by 24 h (Supplementary Fig. 23).

Immunohistological evaluation of brains collected 2.5 h after systemic administration of HerOND revealed robust HER3 associated with the brain vasculature (delineated by the tight junction marker claudin-5) (Fig. 3e), while NBPs overlapped both the vasculature and extravascular parenchyma (Fig. 3f), suggesting that the NBPs may extravasate in the brain. Analysis of serial visual planes along the *z* axis of brain specimens revealed that HPK and NIR-OND coincided both within the vessel and within the parenchyma outside of the vessel, whereas non-overlapped NIR-OND was low to negligible in both locations (Fig. 3f), suggesting that the cargo remained associated with NBPs in both vasculature and parenchyma. Brains from non-tumour-bearing mice not receiving NBPs were compared to non-diseased adult human brain, and both species showed prominent HER3 coinciding with brain vasculature (claudin-5), whereas HER3 was nearly undetectable in the extravascular brain parenchyma (Fig. 3g,h and Supplementary Fig. 24), suggesting that HER3 inherently associates with the brain vasculature.

## HER3 associates with NBP transit across a human BBB model

**Fig. 4 |:**
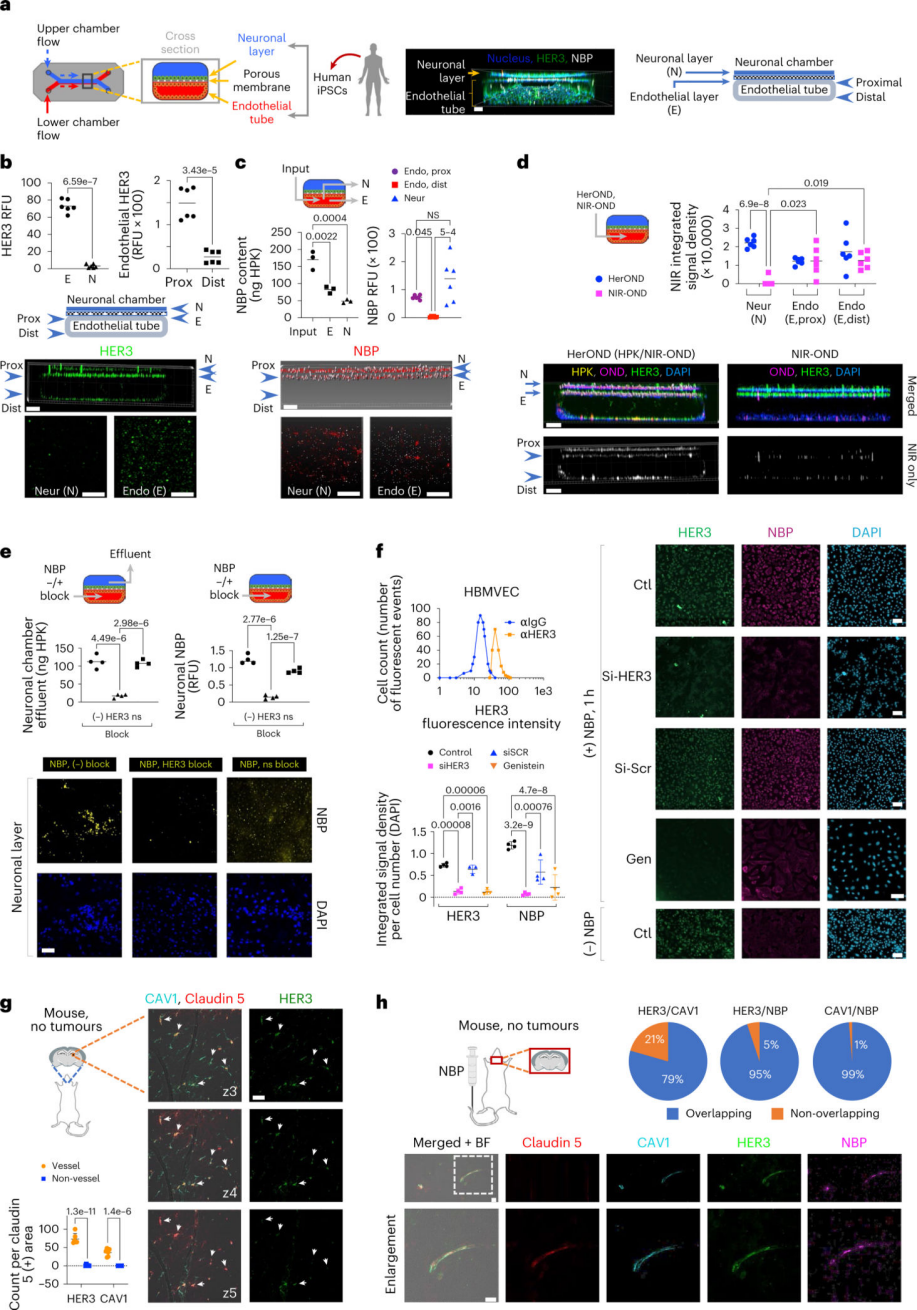
HER3 and NBP in the BBB. **a**, Left: BBB chip schematic; middle: 3D chip imaging 24 h after NBP injection into the endothelial flow chamber; right: cross-sectional map of BBB chip. **b**, HER3 in BBB chips showing cross-sectional and 2D surface views of neuronal (N) and endothelial (E) layers. Graphs show the relative HER3 levels on N and E surfaces and on proximal and distal endothelium (*n* = 6). Significances determined by two-tailed unpaired *t*-tests (95% CI). **c**, BBB chips 4 h after endothelial flow of NBPs delivering NIR-OND, showing cross-sectional and 2D surface views of N and E layers. Graphs, NBP content (left) collected from endothelial (E) and neuronal (N) chamber effluents (n = 3), and (right) immunodetection on proximal versus distal endothelial and neuronal layers (*n* = 6). Significances determined using one-way ANOVA (95% CI) and Tukey post hoc test. **d**, BBB chips 4 h after endothelial flow of NBPs delivering NIR-OND versus NIR-OND alone. Significances determined using two-way ANOVA (95% CI) and Šídák post hoc test (*n* = 6). **e**, NBP content in neuronal chamber effluents (left graph) and immunodetection in neuronal layer during 4 h of endothelial flow ± non-specific (ns) or HER3 blocking. Significances determined using one-way ANOVA (95% CI) and Tukey post hoc test (*n* = 4). **f**, Cell surface HER3 (upper graph; αIgG, secondary antibody alone) and NBP entry of HBMVECs (immunofluorescence imaging and quantification) ± silencing by HER3 siRNA (siHER3) versus scrambled siRNA (siSCR) or genistein treatment (Gen), captured at 1 h after NBP uptake. Significances determined using two-way ANOVA (95% CI) and Šídák post hoc test (*n* = 4). Data presented as mean ± s.d. **g**, HER3 and caveolin-1 (CAV1) localization relative to vasculature (claudin-5) in brains of non-diseased 8-week-old female BALB/c mice, showing 1 μm serial planes (z3–z5) along the viewing *z* axis. Arrows, HER3-positive areas aligning with CAV1 and claudin-5-positive areas. Graph compares each biomarker at vessel versus non-vessel areas. Significances determined using two-way ANOVA (95% CI) and Šídák post hoc test (*n* = 6). Data presented as mean ± s.d. **h**, Localization of NBP, HER3, CAV1 and claudin-5 in brains of 8-week-old female BALB/c mice at 4 h after systemic delivery of NBPs. Scale bars, 150 μm (**a**), 100 μm (**b**–**d**), 50 μm (**e**,**h**), 30 μm (**f**,**g**).

NBP extravasation in the human BBB could be evaluated using an organ chip (BBB chip) derived from iPSCs differentiated into apposing neuronal and endothelial layers to recapitulate the brain–vascular interface^38–40^. Channels creating the closed vessel and overlaying neuronal tissue enable molecular flow through the endothelial tube (Fig. 4a), the integrity of which is validated using fluorescently labelled dextrans of various sizes^38–40^. In support, the brain microvascular endothelial cells (BMECs) comprising the endothelial tube express tight junction proteins including zona occludens-1, glucose transporter-1 and occludin^38–40^, with claudin-5 displayed exclusively in the endothelial layer in contrast to the neuronal layer (Supplementary Fig. 25). The endothelial surface displayed considerable HER3 in contrast to the neuronal layer with higher densities on the proximal (Prox) endothelial surface abutting the neuronal layer compared to the distal (Dist) endothelium (Fig. 4d). HerOND (carrying NIR-OND) flowing through the endothelial tube preferentially localized at the endothelial–neuronal (proximal) interface rather than at the distal endothelial surface (Fig. 4c), and the majority of cell-bound NBPs coincided with HER3 on the proximal surface (Supplementary Fig. 26). Approximately 42% of NBPs injected into the endothelial tube were collected from the neuronal chamber effluent (Fig. 4c), suggesting that HerOND crossed the endothelial barrier. In agreement, HerOND was detected in the neuronal chamber (Fig. 4c,d), whereas free NIR-OND was largely retained in the endothelial tube (Fig. 4d), consistent with the inability of free nucleic acids to permeate cell membranes and the vasculature^41^, and further confirming endothelial tube integrity, suggesting that endothelial passage requires HPK. Interfering with HER3 binding by ligand inhibition (compared with a non-specific competitor, BSA) significantly reduced NBP emergence into the surface and effluent of the neuronal chamber (Fig. 4e), suggesting that BBB transit requires HER3. Taken together these findings suggest that HPK mediates BBB passage and HER3 contributes to this activity.

Commercially obtained human brain microvascular endothelial cells (HBMVECs) enabled confirmation that HER3 is directly present on isolated endothelial cells and that silencing HER3 significantly reduced binding and uptake of HerOND (Fig. 4f). Caveolae have been implicated as facilitating BBB transcytosis of viruses and macromolecules^42,43^, and therapeutic delivery across the lung endothelium^44^, and hence we assessed the contribution of caveolae to the endocytic transport of NBPs in HBMVEC. The caveolae-endocytosis inhibitor, genistein^45^, significantly reduced CAV1, HER3 and NBP uptake in HBMVECs (Fig. 4f and Supplementary Fig. 27) but not in human HER3+ tumour cells (Supplementary Fig. S27). These findings agree with the considerable *ErbB3* and *CAV1* transcript levels in isolated iPSC-derived HBMVECs (human BMECs)^39^ compared with iPSC-derived neural cells and the *GAPDHS* housekeeping gene (Supplementary Fig. 28). In further support, tumour-free mouse brain specimens show that CAV1 and HER3 coincide on the brain vasculature (Fig. 4g,h, Supplementary Fig. 29 and Supplementary Information) at discrete puncta resembling vesicles (Supplementary Fig. 30). HerOND also coincides specifically with HER3/CAV1/claudin-5-overlapping sites in the brain after systemic delivery in mice (Fig. 4h). Taken together these findings suggest that caveolae associate with HER3-mediated endothelial transit of NBPs.

## Systemic NBPs accumulate in intracranial TNBC tumours

**Fig. 5 |:**
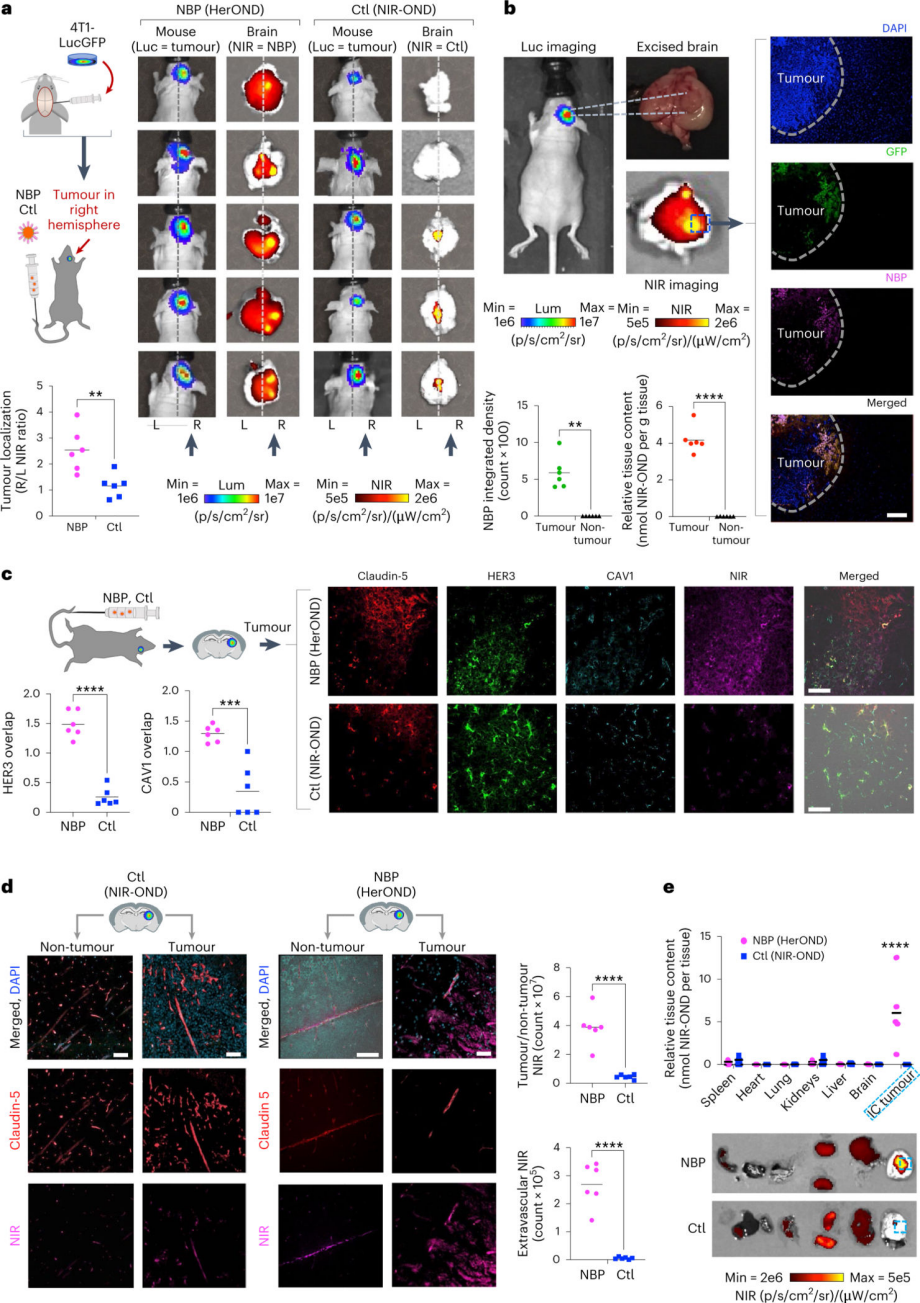
NBP targeting of IC tumours. **a**, NIR signal localization relative to IC TNBC tumours at 4 h after tail vein injection of NBP delivering NIR-OND (HerOND) in 8-week-old female NU/NU mice (*n* = 5 per treatment). Ctl, control (NIR-OND alone). Schematic: stereotactic implant of luciferase (Luc) and GFP-expressing TNBC cells in mouse brains 8 days before single tail vein injections of indicated reagents. Images show BLI of mice (indicating tumour location) and fluorescence imaging of extracted brains (showing localization of NIR-labelled reagent). Vertical arrows indicate tumour locations in right hemispheres. Dashed line delineates left and right hemispheres. Graph summarizes tumour localization of injected reagents. **P = 0.0022 (95% CI) using two-tailed unpaired *t*-test. Lum, luminescence. **b**, Localization of NBPs delivering NIR-OND at 4 h after tail vein injection in 8-week-old female NU/NU mice bearing IC TNBC tumours. Graphs summarize tumour versus non-tumour localization of NBPs based on (left) fluorescence measurement (**P = 0.0013, *n* = 6) and (right) NIR-OND content/tissue weight (****P = 3.2 × 10^−5^, *n* = 6 brains with tumours). Significances determined using two-tailed paired *t*-tests (95% CI). **c**, Comparison of NIR-OND (Ctl) and HerOND (NBP delivering NIR-OND) in IC TNBC tumour regions at 4 h after systemic delivery in 8-week-old female NU/NU mice. Micrographs show channel-separated images of IC tumour specimens counterstained for indicated biomarkers. Graphs summarize the overlap of NBP or Ctl with HER3 and CAV1 in tumour regions. ****P = 3.2 × 10^−5^, ***P = 0.0004 (*n* = 6) using two-tailed unpaired *t*-tests (95% CI). **d**, Comparison of tumour versus non-tumour regions at 4 h after systemic delivery of NIR-OND (Ctl) or HerOND (NBP delivering NIR-OND) in 8-week-old female NU/NU mice bearing IC TNBC tumours. Micrographs show channel-separated images of IC tumour specimens counterstained for indicated biomarkers. Graphs compare NBP and Ctl in tumour versus non-tumour regions (****P = 6.6 × 10^−5^) and in extravascular (claudin-5-negative) regions (****P = 8.5 × 10^−6^) using two-tailed unpaired *t*-tests (95% CI), *n* = 6. **e**, Tissue distribution of NIR-OND (Ctl) or HerOND (NBP delivering NIR-OND) at 4 h after systemic delivery in 8-week-old female NU/NU mice (*n* = 5 per treatment) bearing IC TNBC tumours. Images of harvested tissue show IC tumours delineated by dashed lines. Graph summarizes relative tissue content of NIR-OND. ****P = 6.39 × 10^−6^ (95% CI) using two-way multiple comparisons ANOVA and Tukey post hoc test. Scale bars, 35 μm.

Systemic NBPs carrying NIR-OND (HerOND) showed preferential accumulation in brain right hemispheres of mice bearing right hemisphere intracranial (IC) implants of TNBC tumours (4T1lucGFP expressing luciferase and GFP) in contrast to free NIR-OND (‘Ctl’), which was either undetectable or showed no right-hemisphere preference (Fig. 5a). NBPs coincided with IC tumours (identified by GFP positivity) while being undetectable in non-tumour brain regions (Fig. 5b). NIR-OND was nearly exclusive to claudin-5-positive areas in both tumour (Fig. 5c,d) and non-tumour regions (Fig. 5d), suggesting that free NIR-OND was restricted to the vasculature. In contrast, HerOND showed considerable extravascular spread in the tumour area of the brain in contrast to the non-tumour area in which the NIR signal was considerably lower and restricted to the vasculature (Fig. 5c,d). HerOND but not NIR-OND overlapped considerably with HER3 and CAV1 in tumour regions (Fig. 5c). Phagocytosis by resident macrophages or microglia is likely to be negligible as these cells express low to undetectable *ErbB3* RNA compared with breast cancer cells and may comprise 2.5% or less of the tumour cell population (Supplementary Fig. 31). Meanwhile, systemic NBPs accumulated preferentially in IC tumours compared with non-tumour tissue, in contrast to NIR-OND, which showed considerable accumulation in the kidneys (Fig. 5e). These findings support a mechanism of NBP passage across the BBB via caveolae-associated HER3 and accumulation in IC TNBC using HER3 entry and pH-mediated membrane disruption (Supplementary Fig. 32). Discrimination between endothelial mediated transcytosis versus tumour cell mediated uptake is supported by our observation that a tumoricidal NBP (HerGa) elicits apoptosis of human TNBC cells but not human endothelial cells despite both cell types exhibiting similar cell surface HER3 levels (Supplementary Fig. 33).

**Fig. 6 |:**
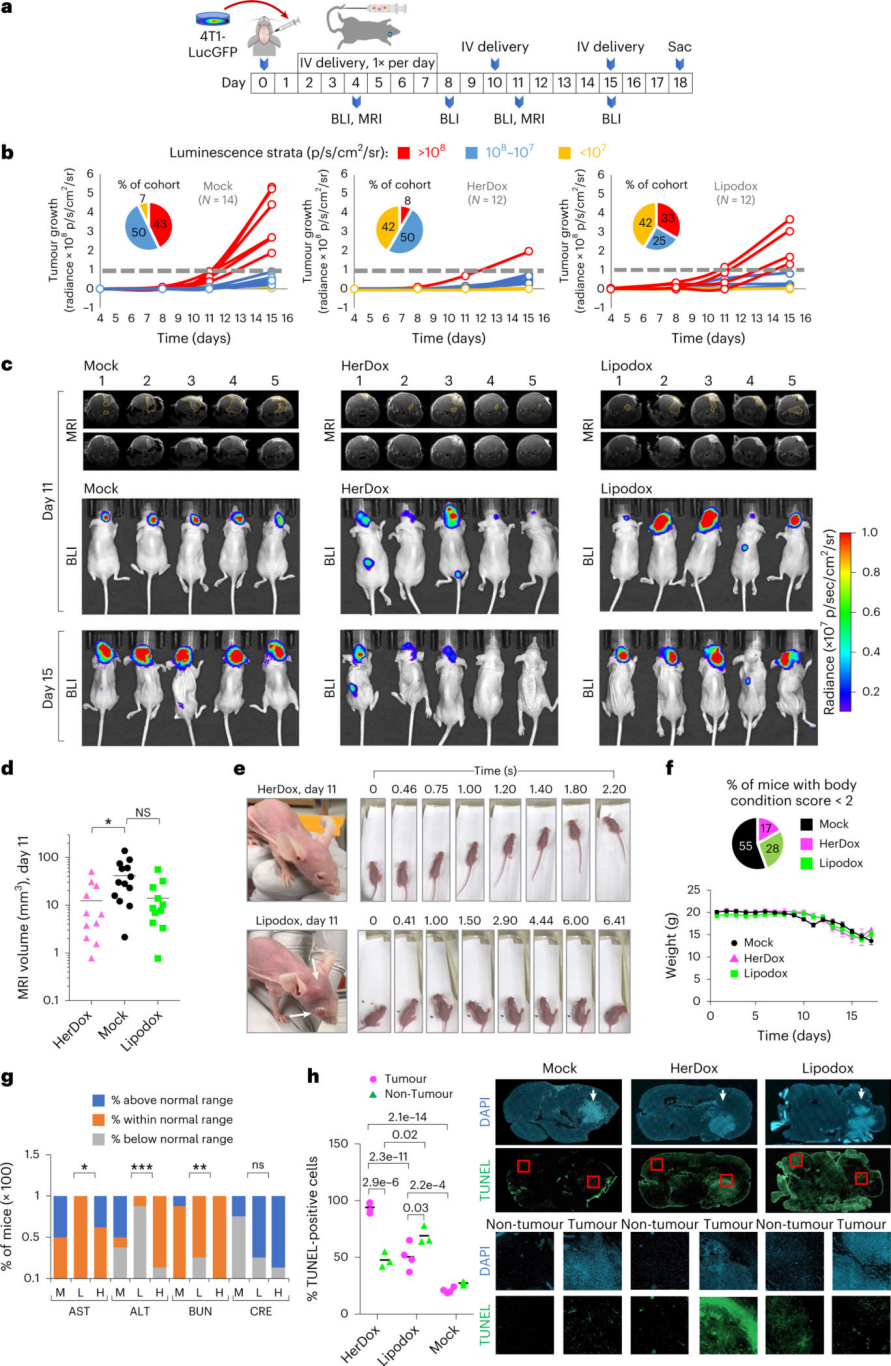
HerDox benchmarked against Lipodox in the IC TNBC model. **a**, Timeline and regimen of tumour implantation, tumour growth monitoring and treatment in 8-week-old female NU/NU mice bearing IC TNBC tumours. BLI and MRI were performed at the indicated time points. **b**, IC tumour growth in mice described in **a** receiving HerDox (*n* = 12), Lipodox (*n* = 12) and mock (*n* = 14) treatments. Each growth curve is designated a colour: accelerated growth, red; slow tumour growth, blue; no detectable tumour growth, yellow. **c**, Brain MRI (coronal view) and BLI of representative mice from each cohort in **b**. MRI images are shown with and without outlines of tumours, as delineated by blinded MRI core staff. **d**, Quantification of IC tumour volumes determined by MRI at mid-treatment (day 11 of regimen). Data represent the mean and individual measurements of each cohort. *P = 0.044 (HerDox, Lipodox: *n* = 11; mock, *n* = 13) determined by one-way multiple-comparisons ANOVA (95% CI) and Kruskal–Wallis test. **e**, External health and mobility of representative mice from HerDox- and Lipodox-treated cohorts on day 11. Arrows indicates extracranial tumour growth with disease affecting the eye. Image stills were extracted from videos in the Supplementary Information. **f**, Average weights of each cohort (mean ± s.d.) throughout the experimental timeline (HerDox, Lipodox: *n* = 12; mock, *n* = 14; at beginning of treatment). Pie chart summarizes the distribution of mice requiring euthanasia due to BCS < 2. **g**, Blood analytes from treated mice. M, mock; L, Lipodox; H, HerDox; AST, aspartate transaminase; ALT, alanine transaminase; BUN, blood urea nitrogen; CRE, creatinine. Significances show comparison of HerDox versus Lipodox groups. *P = 0.0228 (AST), ***P = 0.0005 (ALT), **P = 0.0099 (BUN), determined by unpaired *t*-tests at 95% CI (*n* = 8 per cohort). **h**, Detection of apoptosis by TUNEL stain in brain specimens from treated mice. Channel-separated images show tumour and non-tumour areas. Statistical significances determined using one-way multiple-comparisons ANOVA and Tukey post hoc test at 95% CI (*n* = 3 independent fields each averaging 227 cell events). Arrows point to tumors within brain specimens.

To evaluate therapeutic efficacy, we compared HerDox against Lipodox in mice bearing IC 4T1lucGFP tumours and monitored tumour growth by bioluminescence imaging (BLI) and magnetic resonance imaging (MRI) (Fig. 6a). Stratification of the outcomes revealed three phenotypes distinguishing HerDox from Lipodox treatments. The first was characterized by accelerated growth once tumours exceeded 10^8^ relative luminescence units (RLU) accompanied by concomitant decline in health and was observed in 43% (6/14) of Mock-treated and in 33% (4/12) of Lipodox-treated animals but only in 8% (1/12) of HerDox-treated animals 15 days after tumour implantation (red data, Fig. 6b). The remaining mice in each cohort displayed the second phenotype, characterized by luminescence maintained below 10^8^ RLU, observed in 92% of the HerDox cohort compared with 57% of the Mock and 67% of the Lipodox cohorts (combined blue and yellow data, Fig. 6b). A subset of this second phenotype was characterized by negligible tumour growth, observed in 42% of both the HerDox and Lipodox cohorts but in only 7% of the Mock cohort (yellow data, Fig. 6b). HerDox significantly reduced overall tumour growth rates relative to Mock-treated mice as observed by BLI and MRI (Fig. 6c,d and Supplementary Fig. 34), whereas Lipodox produced a broad range of effect (Supplementary Fig. 35). At mid-study (day 11) representative members of the Lipodox-treated group showed worsened health deterioration, including extracranial metastases and reduced ambulatory ability, whereas such characteristics were not detected in the HerDox-treated mice at this time point (Fig. 6e and Supplementary Information). Considerably higher proportions of Lipodox-treated (71%) and Mock-treated (100%) mice required early euthanasia due to body condition score (BCS) < 2 (Supplementary Table 1) compared with the HerDox-treated (43%) mice, despite similar declines in weight among all cohorts (Fig. 6f). Blood analytes signifying organ toxicity fell within normal range for the majority of HerDox- and Lipodox-treated mice except ALT levels were below normal range for the majority of Lipodox-treated mice in contrast to the HerDox-treated mice, while creatinine levels were above the normal range for the majority of both HerDox- and Lipodox-treated mice while showing no significant differences between cohorts (Fig. 6g and Supplementary Fig. 36). Blood LDH signifying tissue damage showed no significant differences between treated and mock cohorts (Supplementary Fig. 37).

Lipodox treatment elicited greater apoptotic damage to non-tumour brain areas compared with HerDox treatment whereas HerDox elicited significantly higher tumour apoptosis compared with Lipodox treatment and compared with non-tumour cells in the contralateral brain region (detected by terminal deoxynucleotidyl transferase dUTP nick end labelling (TUNEL) stain; Fig. 6h). HerDox treatment also yielded a reduction in tumour HER3 (Supplementary Fig. 38), which taken altogether suggests that HerDox retains the tumoricidal activity of Dox while improving targeted toxicity to brain-localized tumours and preserving healthy brain tissue compared with Lipodox.

**Extended Data Fig. 1 |:**
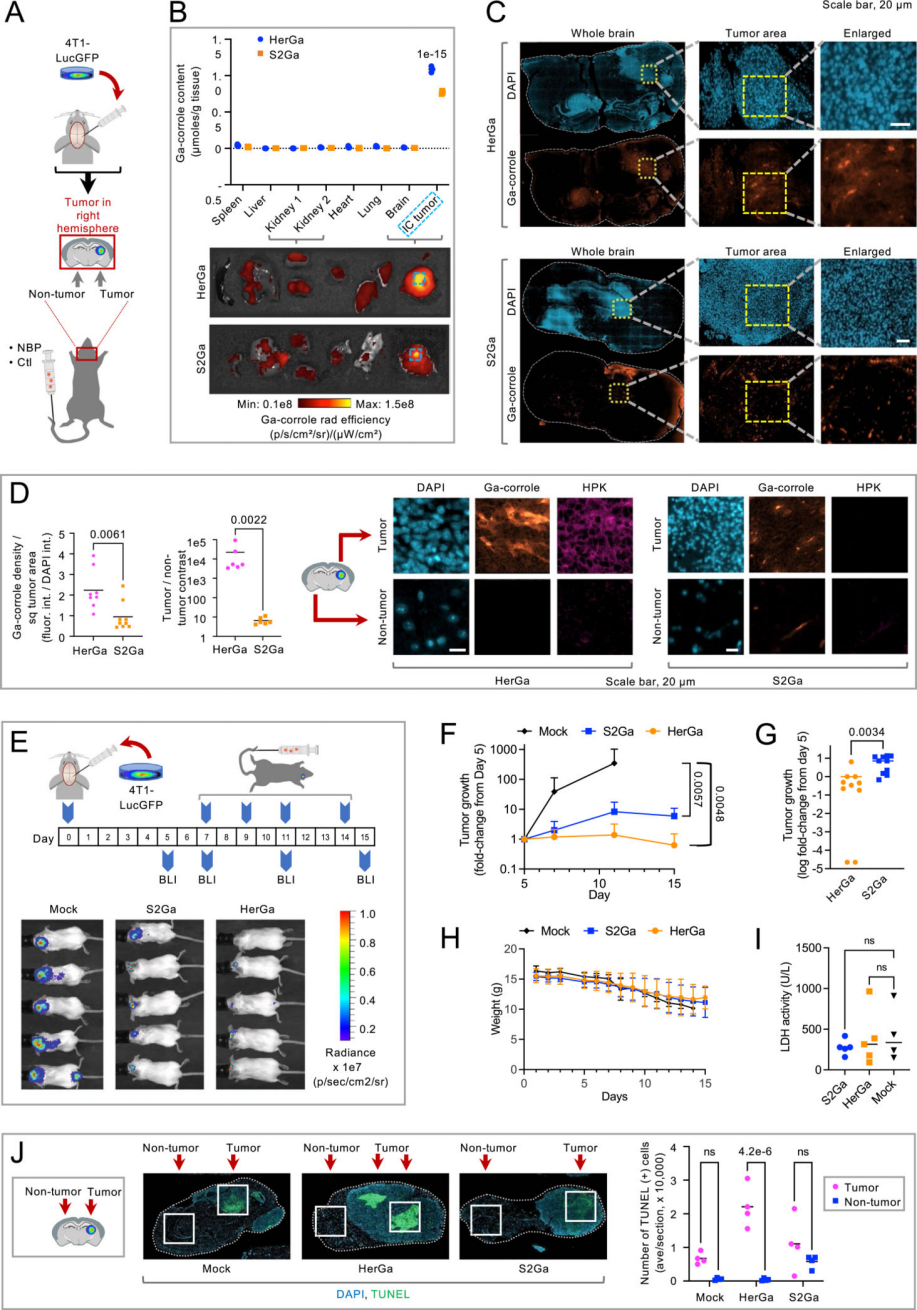
Comparison to serum transferrin (Tf). **A**, Schematic, stereotactic TNBC implant in brains of 8-week female BALB/c mice receiving systemic NBP or control reagents (Ctl) 8 days post-implant. **B**, Tissue-distribution of Ga-corrole comparing delivery by HPK (HerGa) and by serum Tf (S2Ga) after tail vein injection in mice (n = 5/treatment) described in **A**. Images, tissue harvested 4 h after systemic delivery. IC tumours delineated by dashed line. Graph, relative tissue content of gallium(III) metal. Significance compares to all other HerGa and S2Ga tissues (n = 5/treatment) using two-way multiple comparisons ANOVA (95% CI) and Šídák post hoc test. Data represent mean and individual measurements. **C**, Immunohistofluorescence of brain sections from **B** showing localization of Ga-corrole at 4 h after tail vein injection of HerGa or S2Ga. **D**, Tumour vs non-tumour regions from **C** at 4 h after systemic delivery of HerGa or S2Ga. Micrographs, channel-separated images of IC tumour specimens counterstained for nuclei and HPK. Graphs, HerGa vs S2Ga in tumour vs non-tumour regions (n = 6). Data represent mean and individual measurements. **E**, Timeline and regimen of tumour implantation, tumour growth monitoring, and treatment in 8-week female BALB/c mice bearing IC TNBC tumours. Bioluminescence imaging (BLI) performed at indicated time points. Images, BLI of mice from each cohort at experimental endpoints (Mock, Day 12; S2Ga and HerGa, Day 15). **F**, IC tumour growth in mice (n = 5) from **E** receiving indicated treatments. Data represent mean + /-SD. **G**, Tumour growth change in S2Ga vs HerGa treatment cohorts (n = 5/treatment) from **E** at experimental endpoints for each mouse. Each data point represents an individual bioluminescent region of interest (ROI) delineating one or more tumour sites per brain. n= Data represent mean and individual measurements. **H**, Average weights of each cohort (mean ± SD) throughout the experimental timeline in **E** (n = 5/treatment). **I**, Tissue damage assessment by measuring LDH from blood samples (HerGa, S2Ga: n = 5; Mock, n = 4) of mice in ***E*** (ns, no statistical significance). Data represent mean and individual measurements. **J**, Apoptosis detected by TUNEL stain in brain specimens from treated mice in **E**. Tumour and non-tumour areas delineated. Data represent mean and individual measurements (n = 4).

To evaluate HPK bioparticles against molecules that engage more traditional routes of BBB passage for targeting intracerebral tumours, we first compared human gene expression of HER3 (*ErbB3*) against conventional BBB transporters, transferrin receptor (TfR, encoded by the *TFCR* gene) and glucose transporter (GLUT1, encoded by the *SLC2A1* gene). *ErbB3* RNA is significantly increased in brain metastatic TNBC (Supplementary Fig. 39), and significantly decreased in the peripheral (non-central nervous system) endothelia and other normal non-central nervous system tissue compared with *TFCR* and *SLC2A1* transcripts (Supplementary Fig. 40). At the protein level, all three receptors localize to the vasculature in normal human brain specimens (Supplementary Fig. 41). Next, we leveraged the preferential binding of sulfonated gallium-metallated corrole (S2Ga) compounds to serum transferrin (Supplementary Fig. 42)^46^ to compare transferrin-mediated delivery against HPK bioparticles. Mice with intracranial 4T1lucGFP tumours received either S2Ga or HerGa by systemic delivery through the tail vein (Extended Data Fig. 1a) and the inherent fluorescence of the corrole enabled detection in tissue. Both HerGa and S2Ga showed preferential accumulation in the brain, specifically in the brain-localized tumours, in contrast to extracranial tissue, with HerGa yielding significantly higher tumour accumulation compared with S2Ga (Extended Data Fig. 1b). Closer inspection reveals that HerGa exhibited penetration and diffusion into the intracerebral tumour cells, whereas S2Ga collected interstitially (Extended Data Fig. 1c), with HerGa yielding a significantly higher tumour to non-tumour contrast ratio compared with S2Ga (Extended Data Fig. 1d). Systemic HerGa was evaluated for therapeutic efficacy in BALB/c mice bearing intracranial TNBC (4T1lucGFP) tumours resulted in considerable reduction of tumour growth in contrast to S2Ga as observed by BLI (Extended Data Fig. 1e) and luciferase activity during tumour progression (Extended Data Fig. 1f) and at experimental endpoints (Extended Data Fig. 1g). HerGa treatments corresponded to notable extension of survival compared with mock treatments and showed a modest improvement over S2Ga treatment (Supplementary Fig. 43). Blood analytes from HerGa treatments (AST, ALT, BUN) fell within the normal range (Supplementary Fig. 44). Whereas all mice showed similar weight loss (Extended Data Fig. 1h) and no detectable difference in LDH levels signifying tissue damage (Extended Data Fig. 1i), brain histologies revealed that HerGa induced significantly greater tumour-specific (versus non-tumour) apoptosis (detected by TUNEL stain) compared with S2Ga which elicited modest TUNEL signal in both tumour and non-tumour regions (Extended Data Fig. 1j). Tumour specimens from HerGa treatments also exhibited downregulation of HER3 and the mitochondrial metabolic marker, TSPO, in contrast to S2Ga treatment (Supplementary Fig. 45), agreeing with the mitochondrial targeting of corroles once inside the tumour cell^33^.

## Conclusions

This study suggests that NBP interaction with HER3 activates a singular route for ligand-directed nanocarrier transfer across the BBB and into IC tumours, and offers advantages over using conventional BBB transporters, TfR and GLUT1, for targeting brain metastases. HPK may have utility for distinguishing distinctly different transport mechanisms in endothelia versus tumour cells entailing non-degradative CAV1-dependent transendothelial transport followed by tumour entry through an endosomolytic pathway. This may have broad applicability as a growing array of aggressive tumour types express high HER3, which in turn associates with brain metastases and brain-localized tumours^19,47–49^. Our studies delineating the endosomolytic mechanism were limited to tumour cells and thus have not definitively demonstrated that such a mechanism is lacking in endothelial cells. Additionally, our findings cannot discount the possibility that supportive cell types closely associated with the vasculature could contribute to the extravasation functions observed here. These possibilities are being explored further in ongoing studies.

### Online content

Any methods, additional references, Nature Portfolio reporting summaries, source data, extended data, supplementary information, acknowledgements, peer review information; details of author contributions and competing interests; and statements of data and code availability are available at https://doi.org/10.1038/s41565-025-01867-7.

## Methods

### Recombinant protein production

See Supplementary Methods.

### Structural modelling and MD simulation

The HPK monomeric and pentameric structures were generated as previously described^50^. A truncated version of the HPK monomer and pentamer was built (tHPK) to model the effect of pH on the titratable residues in the penton base core of the HPK pentamer. The protein sequence of tHPK monomer was: TGGRNSIRYSELAPLFDTTRVYLVDNKSTDVASLNYQNDHSNFLTTVIQNNDYSPGEASTQTINLDDRSHWGGDLKTILHTNMPNVNEFMFTNKFKARVMVSRLPTKDNQVELKYEWVEFTLPEGNYSETMTIDLMNNAIVEHYLKVGRQNGVLESDIGVKFDTRNFRLGFDPVTGLVMPGVYTNEAFHPDIILLPGCGVDFTHSRLSNLLGIRKRQPFQEGFRITYDDLEGGNIPALLDVDAYQASLKDDTEQGGGGAGGSNSSGSGAEENSNAAAAAMQPVEDMNDHAIRGDTFATRAEEKRAEAEAAAEAAAPAAQPEVEKPQKKPVIKPLTEDSKKRSYNLISNDSTFTQYRSWYLAYNYGDPQTGIRSWTLLCTPDVTCGSEQVYWSLPDMMQDPVTFRSTRQISNFPVVGAELLPVHSKSFYNDQAVYSQLIRQFTSLTHVFNRFPENQILARPPAPTITTVSENVPALTDHGTLPLRNSIGGVQRVTITDARRRTCPYVYKALGIVSPRVLSSRT.

The pentameric core structure of the tHPK particle was investigated under four different pH conditions (pH 7, 5, 3, 1). The protonation states of the titratable residues were obtained from the propKa server^51,52^, yielding a corresponding net charge on the tHPK bioparticle at different pH values: −60 at pH 7, −5 at pH 5, +255 at pH 3 and +300 at pH 1. The tHPK molecular structures at four pH levels (tHPK7, tHPK5, tHPK3, tHPK1) were relaxed using implicit solvent generalized Born MD simulations with the AMBER ff14SB force field^53^ that are part of the AMBER v.18 simulation package^54^. The protonation states of the titratable residues were set for the specific pH values and not allowed to change during the biophysical simulations. All bioparticle structures were relaxed using 10 ns of simulation time. The pH 5 and 7 (tHPK5/tHPK7) bioparticles were simulated for an additional phase of 50 ns because the pH 1 and 3 (tHPK1/tHPK3) pentameric bioparticles broke apart into monomers within the first phase of 10 ns.

### Particle assembly

See Supplementary Methods.

### Electron microscopy

The Electronic Imaging Center for Nanosystems at the University of California Los Angeles provided fixation and TEM through a core services voucher.

### Electrophoretic mobility shift assay

See Supplementary Methods.

### Serum digest (protection) assay

See Supplementary Methods.

### Dynamic light scattering

See Supplementary Methods.

### Cells

See Supplementary Methods.

### Patient-derived tissue

Deidentified surgical specimens of two independent breast cancer tissues and one normal breast tissue were obtained by informed consent under protocol number 29973 which received ethical approval by the Cedars-Sinai Medical Center Institutional Review Board. Resected breast tissues were immediately placed in cold, sterile DMEM after excision and cut into 2–4 mm pieces before undergoing enzymatic and mechanical dissociation using the gentleMACS Octo Dissociator multitissue kit and protocol (Miltenyi Biotec). Resuspended cells were then promptly plated into flasks, multiwell plates or chamber slides for the indicated treatments. Human brain specimens were received from three fresh male cadaver brains, aged 68, 71 and 76 years (Tissue for Research). Samples were preserved in 10% buffered formalin.

### Cell surface detection of HER3

See Supplementary Methods.

### Receptor binding

See Supplementary Methods.

### Intracellular trafficking

The intracellular trafficking of HPK was evaluated on HER3 + MDA-MB-435 cells (whose relatively broad cytoplasmic areas are conducive to such studies) following our previously established procedures^50^, with the following modifications: 12-well plates containing 10,000 cells per well plated on coverslips were briefly prechilled and exposed to 7 μg HPK per well in Buffer A for 1 h to promote receptor binding but not internalization. Equivalent samples received 100 nM bafilomycin-A1 in Buffer A for 30 min before adding HPK. Plates were then transferred to 37 °C to promote synchronized uptake and intracellular trafficking. Cells were fixed at indicated time points after warming, processed for the immunoidentification of HPK using an antibody that recognizes the polyhistidine tag (RGS-His antibody; Qiagen 1:100), and counterstained with 4,6-diamidino-2-phenylindole (DAPI). Images were acquired using a high-throughput digital microscope (Molecular Devices ImageXpress Pico Automated Cell Imaging System) using a 40× magnification lens. Exposure times for each fluorescence wavelength remained fixed to compare between treatments and time points. Where indicated, vesicular-like sequestration of HPK was quantified by subtracting the measured integrated density (Int D) of extravesicular (e) from the vesicular (v) regions normalized by v, or (Int <di>D_v_ − Int D_e_)/Int D_v_.

Endosome maturation staining antibodies against RAB7 and early endosome antigen 1 (EEA1) were purchased from Abcam (ab50533 1:50 and ab206860 1:100, respectively). Samples were imaged using a Leica SPE laser-scanning confocal microscope with Leica Application Suite X (LAS X) 3.3.0.16799. Acquired images were imported and separated into individual channels. Individual cells in selected channels were delineated, and pixel overlap was evaluated using ImageJ.

### Subcellular fractionation

Subconfluent (70% confluency) HER3 + MDA-MB-435 tumour cells grown in complete media were rinsed with 1 × PBS, serum-starved in Buffer A (DMEM containing 20 mM HEPES, pH 7.4, 2 mM MgCl_2_ and 3% BSA) for 1 h at 37 °C, rinsed with 1 × PBS, detached with 2 mM EDTA/PBS and neutralized with double the volume of 1 × PBS++. An aliquot containing 6 × 10^6^ cells was washed with PBS and resuspended in 0.7 ml Buffer A containing 5 nM indicated proteins (quantified by Bradford Assay). Cells were incubated with rocking for 1 h at 4 °C to promote receptor binding but not uptake, followed by transfer to 37 °C to promote synchronized cell uptake. At the indicated time points, cells were pelleted (10 min, 5,000 rpm, 4 °C) and washed in a mildly acidic buffer (1 ml of 1 × PBS, pH 6) for 5 min to remove the remaining cell surface protein. Cell pellets were then rinsed with 1 × PBS and processed for subcellular fractionation (Qproteome Cell Compartment Kit, Qiagen) following the manufacturer’s protocol. Indicated fractions were isolated, and protein precipitation was performed by incubation in 4 vol of ice-cold acetone for 15 min, followed by pelleting (10 min, 14,000 rpm, 4 °C), removal of the supernatant and resuspension in storage buffer (10% glycerol and 5% SDS in dH_2_O). Samples were subject to reducing sodium dodecyl sulfate–polyacrylamide gel electrophoresis (SDS–PAGE) and immunoblotted using antibodies recognizing recombinant protein (Qiagen RGS·His Antibody 34610, 1:100 in 3% BSA) and corresponding fraction controls: cytosolic (GAPDH; R&D Systems, MAB5718, 1:10,000 in 3% BSA); cytoskeletal (β-actin; R&D Systems, MAB8929, 1:5,000 in 3% BSA); and membrane (TIM23; BD Biosciences, 611222, 1:5,000 in 3% BSA). Primary incubation was performed overnight at 4 °C followed by incubation with anti-rabbit or anti-mouse horseradish peroxidase-containing secondary at room temperature for 2 h (Abcam, goat anti-rabbit AB6721 1:3,000 and goat anti-mouse AB6789 1:2,000, respectively). Immunoblots were imaged using the high-sensitivity setting on a Bio-Rad chemidoc imager.

### BBB chip

The organ-on-a-chip is composed of a flexible polydimethylsiloxane elastomer that contains two closely apposed and parallel microchannels (1 mm × 1 mm top channel; 1 mm × 0.2 mm bottom channel)^55^ separated by a porous, flexible polydimethylsiloxane membrane (50 μm thick, with 7-μm-diameter pores, spaced 40 μm apart, resulting in 2% porosity over a surface area of 0.171 cm^2^ separating the two channels) coated with Matrigel in the top channel, and collagen–fibronectin extracellular matrix in the bottom channel. Cell aggregates (‘EZ-spheres’)^56^ were derived from human cells obtained through Institutional Review Board protocol number 21505. To generate BBB chips, EZ-spheres containing iPSC-derived neural progenitor cells were dissociated into single cells using accutase and were seeded into the top channel (‘brain channel’) at a density of 1.25 × 10^6^ cells ml^−1^ in terminal differentiation media (TDM, containing Rock inhibitor 1:2,000 (Stemgent)). The neural progenitor cells were allowed to settle for 2 h and were then flushed with TDM without Rock inhibitor. Media was replaced with 100 μl TDM every other day. Five days later, human induced pluripotent stem cell-derived brain microvascular endothelial-like cells were seeded into the bottom channel (‘vascular channel”) at 15 × 10^6^ cells ml^−1^ in S3 BMEC medium containing Rock inhibitor (1:2,000) and inverted for 2 h. A second seeding was performed after 2 h using the same protocol without inversion for 2 h. Following the second incubation period, the BBB chips were flushed with S3 BMEC medium without Rock inhibitor. The following day, chips were flushed with fresh TDM and S4 BMEC medium. The following day, the chips were added to Emulate pods and placed on an active flow of 30 μl h^−1^. Chips were validated via paracellular permeability assays using dextran–fluorescein isothiocyanate overnight to confirm the barrier function of the chips under flow before release to investigators for experimental testing. Validated BBB chips were treated with NBPs at a concentration equating to 1 μg ml^−1^ of HPK that was passed through the endothelial channel with or without 10:1 blocking peptide purchased commercially from Sinobiological (10201-H08H). After 4 h of constant flow, the chips were fixed using 4% paraformaldehyde and subjected to immunocytofluorescent staining. Chips were imaged on a Nikon A1R confocal microscope with NIS Elements v.5.42.01 and IMARIS v.10.2.0 software for image acquisition and analysis. Notably, gene silencing via CRISPR/Cas9 has been a technical and viability challenge in these cells^57^ and hence the contribution of HER3 toward extravasation was examined by ligand inhibition.

### Sandwich ELISA

See Supplementary Methods.

### Animal subjects

Immunodeficient (NU/NU) and immunocompetent (BALB/c) mice were obtained from Charles River Laboratories. All procedures involving mice were performed following protocol numbers 6037 and 5790, which had received ethical approval by the Cedars-Sinai Institutional Animal Care and Use Committee, in accordance with the institutional and national Guide for the Care and Use of Laboratory Animals. The criteria for euthanasia included tumour ulceration, interference with ambulation and access to food and water, or BCS < 2 (emaciation, prominent skeletal structure, little/no flesh cover, visible and distinctly segmented vertebrae)^58–60^. Blood collected when the animals were killed was processed using serum separator tubes (BD Microtainer tube with serum separator additive/gel, Becton Dickinson) following the manufacturer’s protocol and isolated sera were transferred to an external reference lab (IDEXX BioAnalytics) that provided the measurements of serum analytes. Samples were provided in a blinded/anonymous fashion (sample labelling lacked identifying information). Normal ranges for blood analytes of healthy, tumour-free female NU/NU and BALB/c mice were obtained from Charles River Laboratories^61,62^.

### Tumour models

Peripheral breast tumour models used for the biodistribution and therapeutic efficacy studies were established in 6-week-old female mice. For xenograft models, immunodeficient (NU/NU) mice received bilateral flank implants of JIMT-1 human HER2+ breast tumour cells (1 × 10^7^ cells per implant). For immune-competent models bearing peripheral TNBC tumours, BALB/c mice received bilateral mammary fat pad injections of 4T1LucGFP cells (1 × 10^4^ cells per injection in 0.1 ml PBS). Bioluminescences of 4T1LucGFP tumours were acquired as described later below. Primary tumour volumes (height × width × depth) were monitored approximately three times per week under single-blinded conditions (treatment groups unknown to the individual acquiring measurements). For evaluating therapeutic efficacy, mice were randomly allocated at tumour establishment (≥100–150 mm^3^) into separate treatment groups (*n* = 5 mice per group) and received the indicated therapeutic reagents or controls by tail vein injection twice per week for 4 weeks. HerDox and lipodox dosages equated to 0.2 mg kg^−1^ based on doxorubicin content. Additional cohorts shown in the Supplementary Materials received 0.02 mg kg^−1^ HerDox. Empty particles (lacking the doxorubicin) equated to the 0.2 mg kg^−1^ dose. Saline (mock) treatments were administered at equivalent volumes as the experimental reagents. HerGa and S2Ga dosages equated to 0.2 mg kg^−1^ based on gallium corrole content. Because the HPK protein mediates the receptor-targeted delivery of NBPs and targeting to tumours is largely dependent on and limited by HER3 cell surface levels, in vivo dosage concentration, dosing number and frequency were determined based on the following parameters: circulating blood concentration of drug was based on the minimally effective concentration for reducing HER3+ tumour cells (4T1 mouse TNBC) but not HER3− non-tumour cells (NIH3T3 mouse fibroblasts). From the minimally effective concentration, we could then determine the therapeutically effective ratio of drug molecules to cell number (drug to cell ratio) and extrapolate this to the estimated cell number in the tumours in vivo, using primary tumour size as an initial gauge^63^. For tumours measured based on bioluminescence, cell numbers were estimated from a calibration curve plotted from the bioluminescence measurements of known tumour cell titrations implanted in control mice. The total desired accumulation of drug in the tumour (drug to cell ratio) informed the total number of doses to administer at the determined circulating blood concentration of drug. Frequency of dosing is based on the time course of biodistribution and tissue clearance in tumour models. These same parameters were used to design the treatment regimen in mice with IC tumours as described below.

For the implantation of IC tumours, anaesthetized 4-week-old female mice were positioned in a stereotactic frame, and a burr hole was created in the skull using a steel bit 2 mm right of the sagittal and 2 mm anterior to the lambdoid suture. A stereotactic frame was used to guide a Hamilton syringe, and 10,000 cells in 2 μl were implanted at 4 mm depth. After implantation, bone wax was used to seal the hole, and the incision was sealed with surgical staples, which were removed 5 days later. Immunodeficient NU/NU mice were used for evaluating HerDox and Lipodox (*n* = 12 for HerDox and Lipodox; *n* = 14 for mock). Treatments were delivered via the tail vein at a dose of 0.004 mg kg^−1^ based on doxorubicin content at a regimen meeting the parameters described earlier. Immunocompetent BALB/c mice were used to evaluate HerGa and S2Ga (*n* = 5 per treatment group), each delivered at 0.2 mg kg^−1^ based on gallium corrole content at a regimen meeting the parameters described earlier. Tumour growth was monitored via luciferase beginning on day 4 after implantation and randomized before treatments.

To ensure rigour, all measurements were collected in blinded fashion with cohort identities unknown to the researcher.

### Bioluminescence and NIR fluorescence acquisition

Luciferase monitoring was performed every 4 days, beginning on day 4 postimplantation. Mice received 200 μl intraperitoneal of 30 mg ml^−1^ D-Luciferin (Caliper) dissolved in Dulbecco’s PBS 10 min before imaging using an in vivo imaging system (IVIS). D-Luciferin was allowed to circulate in the animals for 15 min, followed by imaging using a PerkinElmer IVIS Lumina Spectrum. Total flux (photons s^−1^) signals were quantified using equally sized regions of interest (ROIs) centred around the cranial region in LivingImage software v.4.8.0.

NIR image acquisition was performed on freshly excised organs at indicated time points. Average radiant efficiencies ([p/s/cm^2^/sr]/[μW/cm^2^]) were quantified using ROIs outlining individual organs in LivingImage software.

### MRI

A 9.4 T MRI system (BioSpec 94/20USR, Bruker) was used for imaging tumour locations and volumes. The tumour perimeters were visually highlighted by intravenous injections of 7.5 μmol gadovist contrast agent. Mice were imaged under inhaled 1.7% isoflurane anaesthetic. Images were collected with an in-plane resolution of 70 μm using an acquisition matrix of 256 × 196 and zero filling in the phase encoding direction to 256, using a field of view of 1.80 cm × 1.80 cm. Twenty consecutive 0.7 mm slices covered the tumour-implanted region. Two averages were collected, with a repetition time of 750 ms and an echo time of 8.77 ms, for a total scan time of 4.9 min using a mouse four-channel brain array coil (T11071V3, Bruker) for reception and a whole-body transmission coil (T10325V3, Bruker) for excitation. Volume calculations for positive contrast brain regions were determined by integration over the entire tumour by a blinded analyser. For each slice containing tumour-enhanced regions, an ROI was drawn to encompass the area. The area of each slice was multiplied by the slice thickness, and the individual volumes were summed to determine the area of the tumour. Bruker Paravision 5.1 software was used for the analysis. MRI core staff performed quantifications in a blinded fashion.

### Biodistribution

For the quantification of particle delivery by ICP-MS, each mouse was administered a single tail vein injection of HPK bioparticles loaded with gallium(III)-metallated corrole (HPK-S2Ga or HerGa)^64^ or S2Ga alone, at 1.5 nmol S2Ga per injection. At 6 h after injection (*n* = 2 per treatment), mice were killed, and the major organs (brain, heart, kidneys, liver, lungs, spleen and tumours) were excised, weighed and transferred to the University of California Los Angeles ICP-MS core facility. Samples were digested overnight and processed by ICP-MS to measure the tissue content of gallium(III) metal.

Each mouse receiving NBPs (*n* = 5) was administered a single tail vein injection of HPK bioparticles loaded with NIR Alexa Fluor 680-labelled oligonucleotides at a dose equal to 1.50 nmol oligonucleotide per injection. Mice were monitored by epifluorescence imaging at the indicated time points after injection using an IVIS Spectrum (PerkinElmer), followed by tissue harvesting and the acquisition of average radiant efficiency ([p/s/cm^2^/sr]/[μW/cm^2^]) per tissue. Where indicated, the relative tissue content of NIR-OND was determined based on the extrapolation of measurements acquired from extracted tissue against a standard curve of known NIR-OND titrations in tissue lysates. Tumours were implanted 8 days before mice received systemic NBPs, allowing sufficient time for damaged vessels to repair, and NIR-OND alone was used to detect the possibility of vascular leakage.

Mice (*n* = 5 per treatment) receiving directly labelled protein or particles (NIR-labelled HPK capsomeres, Tz or BSA) were injected with the indicated treatment through a single tail vein injection at 12 nmol of labelled protein per injection. Proteins were labelled with Alexa Fluor 680 at primary amines and isolated from the unconjugated dye by size-exclusion chromatography using a commercial protein-labelling kit, following the manufacturer’s protocol (LifeTechnologies).

### Immunogenicity assay

Female BALB/c mice (∼6 weeks old; Charles River) received tail vein injections of empty (no drug) NBPs at 0.5 mg kg^−1^ HPK per injection (equating to ~0.2 mg kg^−1^ HerDox) twice per week for four weeks. Serum isolated from blood collected at indicated time points underwent serial dilutions to 1 × 10^−4^ dilution and was processed by ELISA using either empty NBPs (5 μg ml^−1^, 0.5 μg per well) or Ad5 (5 × 10^6^ PFU per well) as capture antigens. Mouse Ig was detected using horseradish peroxidase-conjugated anti-mouse antibody, and ELISAs were performed according to standard procedures. Serial dilutions of mouse Ig were used as reference antibody titres.

### Immunocytofluorescence and immunohistofluorescence

See Supplementary Methods.

### TUNEL assay

See Supplementary Methods.

### Statistics and reproducibility

Except where indicated, in vitro data are presented as the mean of triplicate samples ± s.d. from at least three independent experiments. For normally distributed in vitro data, significant differences were determined by one-way analysis of variance (ANOVA) followed by Tukey’s post hoc analysis, unless otherwise indicated. In vivo data are presented as mean ± s.d. Non-parametric analyses were used to determine significant differences within in vivo experiments, where appropriate, using Kruskal–Wallis tests followed by Mann–Whitney post hoc analysis. All measurements were taken from distinct samples.

### Reporting summary

Further information on research design is available in the Nature Portfolio Reporting Summary linked to this article.

## Extended Data

**Extended Data Table 1 |:** Particle characteristics

	HerOND	HerDox	HerGa
Diameter (nm) ^a^	42.6 ± 1.2	42.8 ± 9.7	42.8 ± 9.3
PDI ^a^	0.13 ± 0.007	0.27 ± 0.032	0.28 ± 0.018
Stoichiometry, 1× ^b^	3:1 HPK:OND	1:0.2:2.8 HPK:OND:Dox	1:30 HPK:S2Ga
Stoichiometry, 5× ^b^	2.7:1	1:0.15:4.1	1:39
Stoichiometry, 10× ^b^	2.9:1	1:0.3:2.7	1:26
Zeta potential (mV) ^c^	−33.3 ± 0.91	−22.2 ± 0.94	−16.5 ± 2.95
Mobility (μmcm/Vs) ^c^	−1.53 ± 0.06	−1.56 ± 0.7	−1.55 ± 0.21
Conductivity (mS/cm) ^c^	17.6 ± 0.05	17.5 ± 0.72	19.5 ± 0.90
EC50, 4T1 (μM) ^d^	>10	1.1	0.381
EC50, NIH-3T3 (μM) ^d^	>10	>10	>10

a Samples stored at −20 °C for at least 1 month. Five measurements each, consisting of a number of runs determined automatically (ranging between 12–18 runs).

b Protein content determined by denaturing SDS-PAGE of NBPs compared against a standard curve; where applicable, OND content determined by electrophoretic band density after HPK removal by proteinase K digest (see Supplementary Fig. S4); Dox and S2Ga content determined by absorbance and fluorescence measurement sweeps compared against respective standard curves spiked in protein lysates.

c HerOND, 5 measurements each at 20 runs per measurement; HerDox, 6 measurements each at 20 runs per measurement; HerGa, 13 measurements at 20 runs per measurement.

d Minimum of three separate cell culture wells per concentration.

## Supplementary Material

Supplementary Materials

**Supplementary information** The online version contains supplementary material available at https://doi.org/10.1038/s41565-025-01867-7.

## Data Availability

Databases/datasets analysed in the current study are publicly accessible through the following accession codes: brain metastatic TNBC (GSE76714), normal breast (GSE7307), normal brain (GSE3594) RNA-seq datasets; iPSC-derived brain microvascular endothelial cells and neural cells RNA-seq dataset (GSE97324).
