# Supplementary material for: Systemic HER3 ligand-mimicking nanobioparticles enter the brain and reduce intracranial tumour growth
Source: Nat Nanotechnol. Author manuscript; Available in PMC 2025 Jun 7. (PMC12095042; doi:10.1038/s41565-025-01867-7)
Supplement: Supplementary Materials [file NIHMS2077590-supplement-Supplementary_Materials.pdf]

# SUPPLEMENTARY MATERIALS

## Contents


***SUPPLEMENTARY MOVIES***

Supplementary Movie 1. HPK capsomere undergoing protonation. https://www.youtube.com/watch?v=QiX_6bY_hig

Supplementary Movie 2. Z-stacked images of mouse brain specimen stained for HER3, Claudin 5, and CAV1. https://youtu.be/CLIQZziWroE

Supplementary Movie 3. Representative mouse from HerDox cohort. https://youtu.be/LfKK-MbU10g

Supplementary Movie 4. Representative mouse from Lipodox cohort. https://youtu.be/QKFty53h0fE

***SUPPLEMENTARY METHODS***

S1.1 Isothermal titration calorimetry.

S1.2 ITC of HerDox

S1.3 Genomic analyses of HER3 expression in normal and tumor tissues

S1.4. HER3 antibody specificity

***SUPPLEMENTARY TABLES***

Supplementary Table 1. Summary of cohort numbers during treatment of mice bearing IC tumors.

***SUPPLEMENTARY FIGURES***

Supplementary Figures S1-S5, S6-S8. Particle characterization.

Supplementary Figure S9-S13. HER3 patient TMAs and bioinformatics.

Supplementary Figure S14-S18. HER3 cell lines, peripheral tumor models, cytokines.

Supplementary Figure S19-S21. Cell penetration.

Supplementary Figure S22-S26, S27-S28, S29-S30. Brain vasculature.

Supplementary Figure S31. Microglia, tumor cell population.

Supplementary Figure S32. BBB IC delivery overview.

Supplementary Figure S33. NBP on tumor and endothelial lines.

Supplementary Figure S34-S38. HerDox therapeutic efficacy.

Supplementary Figure S39-S41. HER3, TFR, GLUT1.

Supplementary Figure S42-S45. HerGa therapeutic efficacy.

***SUPPLEMENTARY METHODS***

**S1.1 Isothermal titration calorimetry.** Isothermal titration calorimetry (ITC) experiments were performed using a MicroCal PEAQ-ITC at 25°C. Heat changes during assembly of HPK with ssOND were measured after suspending ssOND in RNA annealing buffer (10 mM Tris-HCl, pH. 7.5; 50 mM NaCl, and 1 mM EDTA, in ultrapure nuclease-free water.).

To avoid buffer mismatch, HPK was buffer exchanged into the same RNA annealing buffer. The ssOND (300 µL at 1.0 µM) was placed in a stirred cell and titrated with 5.4 µM HPK at 2 µL HPK/titration in a 2.5 min injection to allow for the titration peak to return to baseline. The dissociation constant ($K_d$) was calculated using MicroCal PEAQ-ITC analysis software and Prism GraphPad software, using the one-site model. Control experiments included titrating HPK into buffer, buffer into ssOND, and buffer into buffer to ensure that measured changes were not due to buffer mismatches or other artifacts. The three controls were combined into a composite and used to subtract the heat of dilution and background noise from the baseline. ITC measurements of the HPK assembly with dsOND and the HerDox assembly are described below.

**S1.2 ITC of HerDox.** The two-step assembly of HerDox requires two separate ITC measurements: 1) binding of Dox to the oligonucleotide duplex, dsLLAA; and 2) binding of HPK to Dox-intercalated dsLLAA. The complementary oligonucleotide duplexes forming dsLLAA were prepared by mixing together equal molar concentrations of the 30-base oligonucleotide, LLAA-5 (5'CGCCTGAGCAACGCGGCGGGCATCCGCAAG-3'), and its corresponding reverse complement LLAA-3 in annealing buffer pH 7.4. The mixture was boiled in a beaker filled with water for 5 min, then the beaker was transferred to the benchtop and allowed to cool to room temperature. All following dilutions of dsLLAA or Dox were done in Sodium Phosphate buffer (pH 7.4) to match the storage buffer of HPK.

*ITC #1) To determine Dox: dsLLAA binding molar ratio*. The ITC experiment was carried out using MicroCal PEAQ-ITC at 25 °C. 300 µL of 4.0 µM dsLLAA in the cell was titrated with 500 µM Dox. Titrations took place by injecting 2 µL Dox in a 2.5 min injection for the titration peak to return to the baseline. The $K_d$ was calculated using the MicroCal PEAQ-ITC analysis software using the one-site model. Control experiments were carried out by titration of 500 µM Dox into buffer (Sod. Phosphate pH 7.4), buffer into 4.0 µM dsLLAA, and buffer into buffer. The three controls were used a composite for the ITC experiment to subtract the heat of dilution and background noise from the baseline.

*ITC #2) To determine the dissociation constant and HPK: Dox-dsLLAA binding molar ratio.* A fresh batch of Dox-dsLLAA was mixed in a molar ratio of 7: 1 Dox:dsLLAA (based on findings from ITC #1) To a final dsLLAA concentration of 12 µM. The mixture was incubated at room temperature for t least 30 minutes. The ITC experiment was carried out using MicroCal PEAQ-ITC at 25 °C. 300 µL of 3.5 µM

HPK in the cell was titrated with 12 μM dsLLAA in the form of Dox-dsLLAA. Titrations took place by injecting 2 μL Dox in a 2.5 min injection for the titration peak to return to the baseline. The $K_d$ was calculated using the MicroCal PEAQ-ITC analysis software using the one-site model. Control experiments were carried out by titration of 12 μM Dox-dsLLAA into buffer (sod. Phosphate pH 7.4), buffer into 3.5 μM HPK, and buffer into buffer. The three controls were used a composite for the ITC experiment to subtract the heat of dilution and background noise from the baseline.

**S1.3 Genomic analyses of HER3 expression in normal and tumor tissues.** All genomic datasets used in our studies are publicly available. The R2 Genomics Analysis and Visualization Platform (http://r2.amc.nl) was used to access the Roth database of normal tissues, The Cancer Genome Atlas (TCGA) database (TCGA-1097) of breast invasive carcinomas, and the Brown-198 database of TNBC samples. The normal database was filtered to only include normal breast tissue (breast, nipple, and breast adipose). HER3 (*ERBB3*), TfR (*TFRC*), and GLUT1 (*SLC2A1*) genes were used to interrogate the Normal Endothelial cell (HUAEC/HUVEC)-Luttun-38 and TNBC metastatic brain tumors-Biernat-71 databases and all normal R2 databases, excluding CNS tissue. The latter encompasses the following individual databases: adrenal (Various-13); B cell (Comerma-24; Johnsen-38; Jima-12; Kauppinen-38; Nussenzweig-8); blood (Tompkins-857; Uyhelji-555; Jarvela-96; Sindhi-26;Tangye-14; Yamaguchi-30; Yamaguchi-30 fRMA; Fioretos-16; Novershtern-211; Villani-1244; Villani-1140); colon (Marra-32; Vivier-4); developmental (embryonic, Tanavde-136; fetal, Wang-4089; fetal, Wang-5290; fetus, Bianchi-40; HES iPSC, Linnarsson-337; HSC, Ogic-21; Stem cell fetal, Xian-24 huex10p; Stem cell fetal, Xian-24 huex10t; embryogenesis, Yi-18; Stem cells, Linnarsson-1715); endothelial (Luttun-38); Epithelial (Shelhamer-9); fallopian tube (Shaw-24); fibroblasts (Mazda-6); leukocytes (Clark-108; Clark-114; Matthes-33 fRMA; Matthes-33); liver (McGilvray-8444; McGilvray-4059); lymphocytes (Goerdt-20; Franken-440; Franken-352; Franken-440; Franken-352; Lye-154; Lye 154 fRMA); macrophages (Salazar-44); mesenchymal (Wezel-15 huex10p; Wezel-15 huex10t); monocytes (PBM-26); muscle (Gordon-22; Hofman-121 u133a; Hofman-121 u133b; Asmann-40); pancreatic (Groop-89; Taneera-63); placenta (Bammler-12); platelets (Shaw-154); skeletal (Stephan-4); spermatogonia (Brinster-6; Spiess-8); T cell (Wicker-35); and thymus (Ferrando-21). Gene expression data is represented as a $\log_2$ transformation.

**S1.4. HER3 antibody specificity**. The Thermo Fisher Scientific H3.105.5 (Ab105) mouse antibody recognizing human and mouse HER3 was used at 1:500 dilution following the indicated methodological procedures as described on patient-derived breast tumor specimens (**Fig. 2D**), human tumor cells for intracellular trafficking studies (**Fig. 2E**), and isolated human brain microvessel endothelial cells (**Fig. 4H**). Antibody specificity was validated by siRNA-mediated knock-down of HER3 transcript expression as described previously (Ref #51), which reduced immunorecognition of HER3 to

undetectable levels on human brain microvessel endothelial cells (**Fig. 4H**). Secondary antibody alone (Invitrogen goat anti-mouse IgG secondary antibody Alexa Fluor® 488 conjugate A-11017 at 1:500 dilution) lacked detectable immunoreactivity in the absence of the primary HER3 antibody on the same cells (**Fig. 4H**). The R&D Systems sheep anti-mouse HER3 antibody (AF4518) which also cross reacts with human HER3 was used at 1:400 dilution on mouse and human tumor and non-tumor cell lines (**Fig. 1L; Supplementary Figs. S15, S24, S30**), TMAs (**Supplementary Figs. S10-S12**), BBB chips (**Fig. 4 A, B, D, E**), and mouse tissue specimens undergoing co-immunostaining for CAV1 (**Fig. 4I-J, 5C-D; Supplementary Fig. S26, S27**) or other biomarkers (**Supplementary Figs. S35, S38, S42**). HER3 specificity was validated by siRNA-mediated knock-down of HER3 transcript expression as described previously,[1] which reduced immunorecognition of HER3 to undetectable levels on human tumor cells (**Fig. 2A**). Secondary antibody alone (Donkey anti-sheep IgG Alexa Fluor® 488 conjugate ab150177 or Alexa Fluor® 647 conjugate ab150179) lacked detectable immunoreactivity in the absence of the primary HER3 antibody on mouse tumor and non-tumor cells (**Fig. 1L; Supplementary Fig. S15**) and human tumor and endothelial lines (**Supplementary Fig. S30**). Immunohistological staining in brain specimens were independently confirmed with a rabbit antibody recognizing both mouse and human HER3 (Cell Signaling D22C5) and a goat anti-rabbit IgG secondary antibody (Cell Signaling Alexa Fluor® 488 conjugate #4412) (**Fig. 3 G, I, J**; **Supplementary Fig. S22**). Secondary antibody alone lacked detectable immunoreactivity in the absence of the primary HER3 antibody on human tumor cell lines (**Supplementary Fig. S9)**

***SUPPLEMENTARY TABLE 1***. Summary of cohort numbers during treatment of mice bearing IC tumors.

| | Mock | HerDox | Lipodox |
|---|---|---|---|
| N [†] | 14 | 12 | 12 |
| Day 15 tissue harvest [‡] | 4 | 5 | 5 |
| BCS<2 [§] | 10 | 3 | 5 |
| Post Day 15[#] | 0 | 4 | 2 |

[†] Total per cohort.

[‡] Sacrificed after final BLI for tissue/blood harvest and comparative assessments.

[§] Requiring euthanasia before endpoint due to no/low mobility and a Body Condition Score of less than 2 (emaciation, prominent skeletal structure, little/no flesh cover, visible and distinctly segmented vertebrae).

[#] Surviving beyond final BLI without requiring euthanasia.

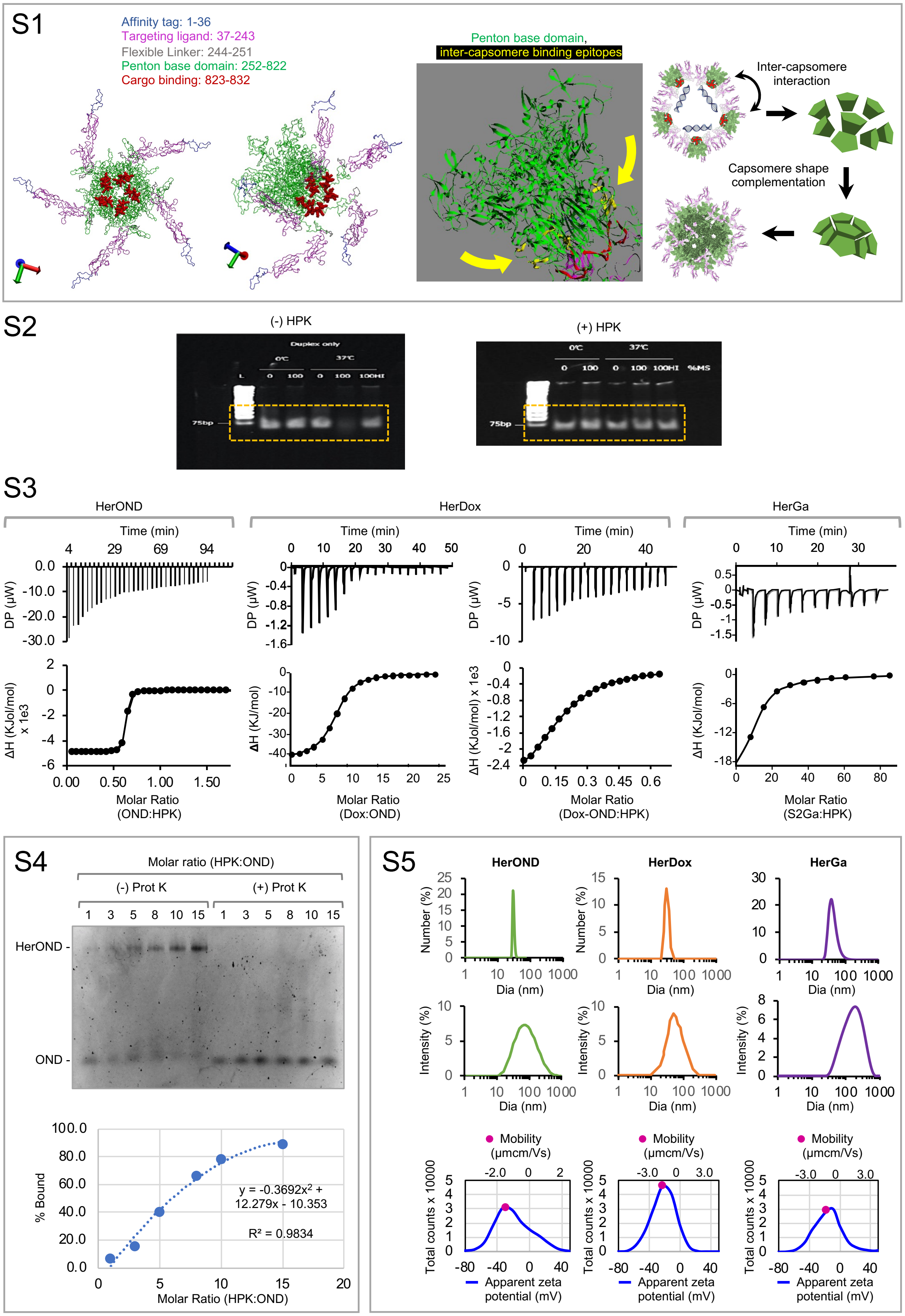
S1
Affinity tag: 1-36
Targeting ligand: 37-243
Flexible Linker: 244-251
Penton base domain: 252-822
Cargo binding: 823-832
Penton base domain,
inter-capsomere binding epitopes
Inter-capsomere interaction
Capsomere shape complementation
S2
(-) HPK
(+) HPK
75bp
S3
HerOND
HerDox
HerGa
Time (min)
DP (μW)
ΔH (KJol/mol) x 1e3
ΔH (KJ/mol)
ΔH (KJol/mol)
Molar Ratio (OND:HPK)
Molar Ratio (Dox:OND)
Molar Ratio (Dox-OND:HPK)
Molar Ratio (S2Ga:HPK)
S4
Molar ratio (HPK:OND)
(-) Prot K
(+) Prot K
HerOND -
OND -
% Bound
y = -0.3692x² + 12.279x - 10.353
R² = 0.9834
Molar Ratio (HPK:OND)
S5
HerOND
HerDox
HerGa
Number (%)
Intensity (%)
Dia (nm)
Mobility (μmcm/Vs)
Total counts x 10000
Apparent zeta potential (mV)

**Supplementary Figure S1.** Left, ribbon structure of the HPK pentamer with functional domains delineated and cargo loading decalysine (K10) shown as filled structure. Middle, computational model of HPK pentamer focused on the penton base domain with arrows pointing to inter-capsomere binding epitopes highlighted in yellow: SELA (aa 309-312), NDYS (aa 350-353), and RSTR (aa 703-706). Right, schematic illustrates how cargo loading through electrostatic binding to the K10 (red) domain may drive capsomere convergence and shape complementation facilitating polyhedra formation.

**Supplementary Figure S2.** Gel electrophoresis and ethidium bromide staining of OND -/+ incubation in serum nucleases −/+ pre-assembly with HPK. H.I., heat-inactivated serum.

**Supplementary Figure S3.** Evaluating binding of HPK with each indicated cargo using isothermal titration calorimetry (ITC). Upper graphs show the raw thermograms used to generate the corresponding binding isotherms (lower graphs).

**Supplementary Figure S4.** EMSA and proteinase K digestion assay to validate cargo (OND) loading into HPK NBPs (HerOND). Gel shows electrophoresed particle titrations loaded after particle assembly or incubated with proteinase K (Prot K) before loading on gel. Each lane contains equivalent amount of OND cargo with titrating concentrations of HPK pre-assembled at indicated ratios. Graph summarizes % of cargo encapsulated in particles based on densitometry of high MW species bands and validated by Prot K digest to release and quantify encapsulated OND.

**Supplementary Figure S5.** Graphs showing dynamic light scattering measurements (number and intensity) of indicated NBPs and corresponding zeta potential and mobility measurements.

***SUPPLEMENTARY FIGURES (S6-S8)***

# S6

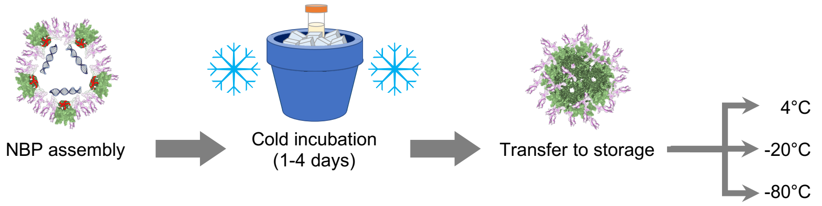


# S7

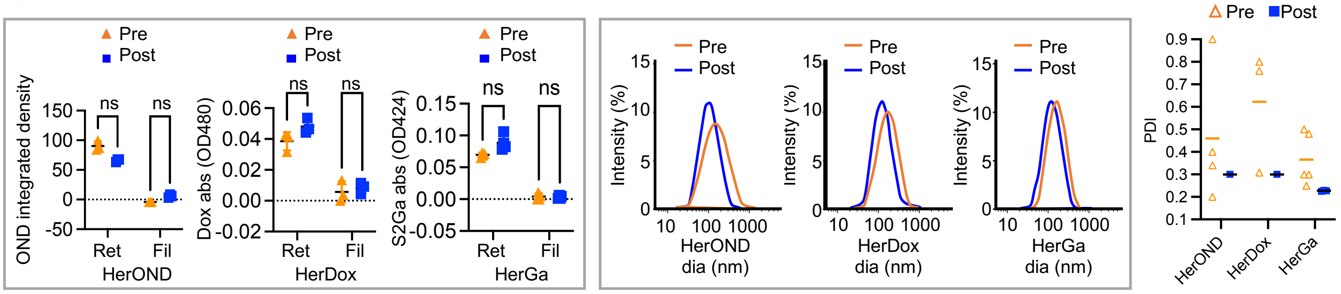


# S8

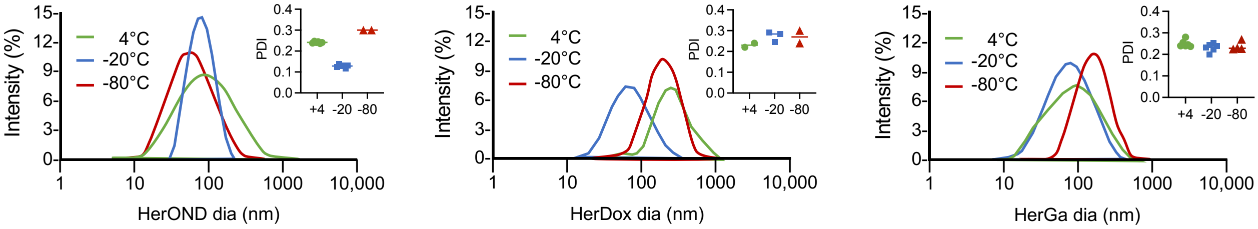

**Supplementary Figure S6.** Schematic illustrating the cold incubation of NBPs before transfer to long-term storage. Immediately after initial assembly of each indicated NBP (as described in the *Methods*), mixtures are incubated 1-4 days on ice in a cold room (4°C) environment. Where indicated, mixtures were evaluated for retention / release of cargo and hydrodynamic diameters / polydispersity before transfer to long term storage at indicated temperatures.

**Supplementary Figure S7.** Stability of NBPs before (pre) and after (post) cold incubation on ice (n=3). NBPs were brought to room temperature before subject to (left 3 graphs) ultrafiltration and (middle 3 graphs) DLS. Retentates (Ret) and filtrates (Fil) were collected at each time point and corresponding cargo measured by gel assay (OND), Dox absorbance and corrole absorbance. Right graph, PDIs of each NBP at the beginning (pre) and after (post) cold incubation as illustrated in *Supplementary Fig. S6*.

**Supplementary Figure S8.** Hydrodynamic diameter measurement of NBPs by DLS after storage at indicated temperatures for at least 1 year. Insets show PDIs of each NBP stored under each condition.

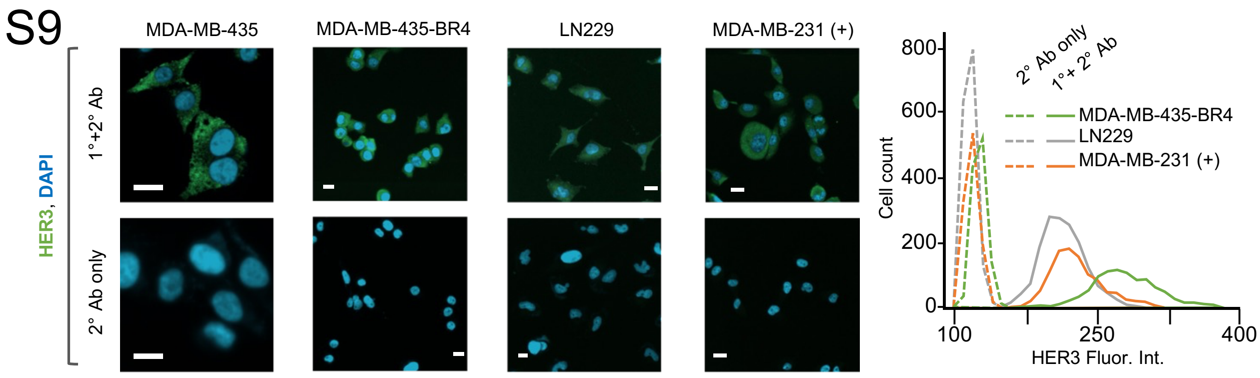
S9
MDA-MB-435
MDA-MB-435-BR4
LN229
MDA-MB-231 (+)
HER3, DAPI
1°+2° Ab
2° Ab only
2° Ab only
1°+ 2° Ab
MDA-MB-435-BR4
LN229
MDA-MB-231 (+)
Cell count
800
600
400
200
0
100
250
400
HER3 Fluor. Int.

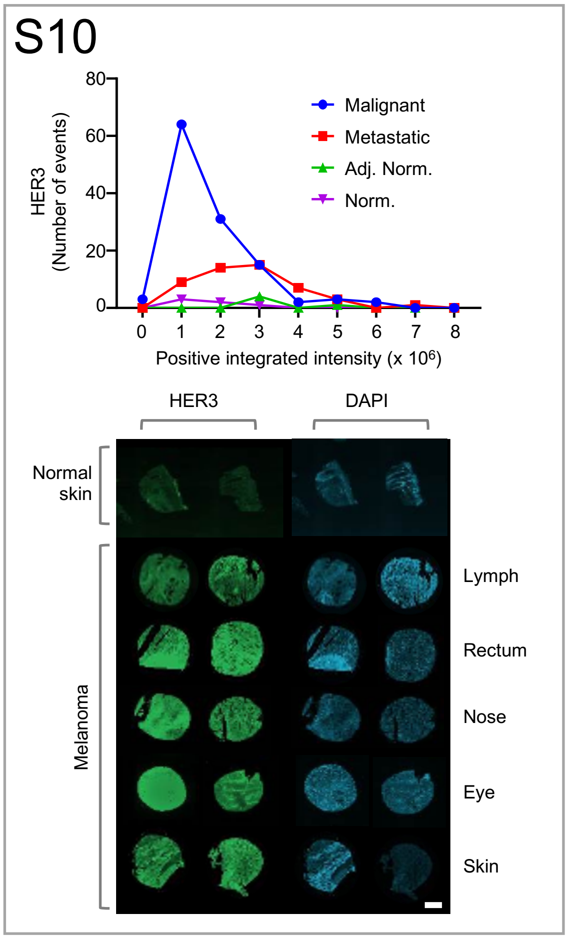
S10
HER3
(Number of events)
80
60
40
20
0
Malignant
Metastatic
Adj. Norm.
Norm.
0 1 2 3 4 5 6 7 8
Positive integrated intensity (x 10^6)
HER3
DAPI
Normal
skin
Melanoma
Lymph
Rectum
Nose
Eye
Skin

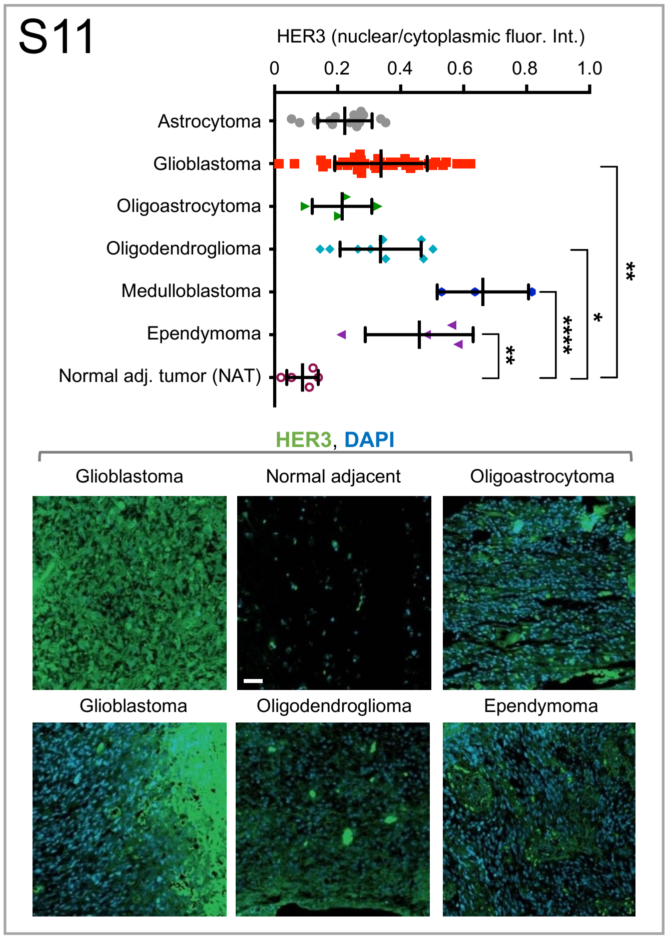
S11
HER3 (nuclear/cytoplasmic fluor. Int.)
0 0.2 0.4 0.6 0.8 1.0
Astrocytoma
Glioblastoma
Oligoastrocytoma
Oligodendroglioma
Medulloblastoma
Ependymoma
Normal adj. tumor (NAT)
**
****
*
**
HER3, DAPI
Glioblastoma
Normal adjacent
Oligoastrocytoma
Glioblastoma
Oligodendroglioma
Ependymoma

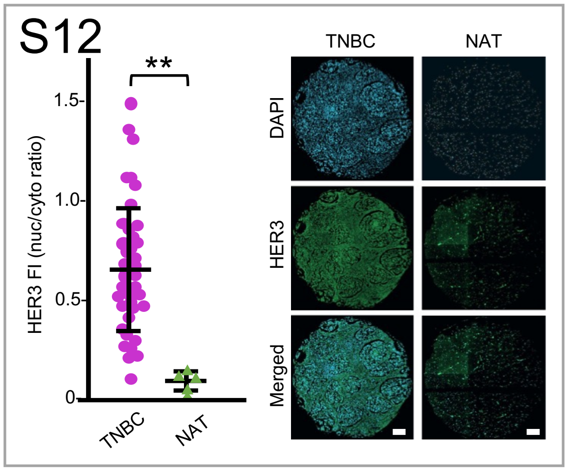
S12
**
HER3 FI (nuc/cyto ratio)
1.5
1.0
0.5
0
TNBC
NAT
TNBC
NAT
DAPI
HER3
Merged

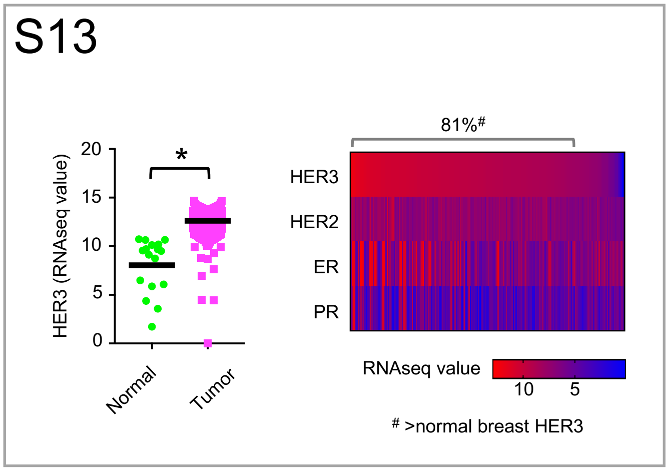
S13
HER3 (RNAseq value)
20
15
10
5
0
*
Normal
Tumor
81%#
HER3
HER2
ER
PR
RNAseq value
10
5
# >normal breast HER3

**Supplementary Figure S9.** Immunocytofluorescence staining of human cancer cell lines using a HER3-specific primary (1°) antibody followed by Alexa488-tagged secondary (2°) antibody compared to the secondary antibody alone. The cell lines shown (obtained from ATCC) represent: melanoma-like tumor cells originally obtained from the pleural effusion of a breast cancer patient (MDA-MB-435) and its brain-metastatic sub-line (MDA-MB-435-BR4), a glioblastoma-derived cell line (LN229), and a TNBC line (MDA-MB-231). Scale bar: 8 µm. Inset, flow cytometry graph summarizing the HER3 fluorescence intensities of each cell line (MDA-MB-435 flow cytometry shown in Fig. 2A).

**Supplementary Figure S10.** HER3 immunofluorescence staining (micrograph) and intensity measurements (graph) of a patient-derived human melanoma tissue microarray (BioMax) showing malignant, metastatic (lymph) and normal skin specimens. Micrograph shows representative samples of high HER3 intensity specimens compared to normal skin specimens.

**Supplementary Figure S11.** HER3 immunofluorescence staining (micrographs) and intensity measurements (graph) of a patient-derived human brain cancer tissue microarray (BioMax) showing several types of brain tumors. Scale bar, 100 µm. Glioblastoma vs. NAT, **p=0.0026; Oligodendroglioma vs. NAT, *p=0.018; Medulloblastoma vs. NAT, ****p=<0.0001; Ependymoma vs. NAT, **p=0.0013.

**Supplementary Figure S12.** Immunohistofluorescence of specimens from a human TNBC microarray. NAT, normal tissue adjacent to tumor. Graph, quantification of HER3 in TNBC (N=48) vs NAT (N=5) from TMA specimens. Scale bar 140 µm. **p=0.0025.

**Supplementary Figure S13.** Graph, HER3 gene expression comparing invasive breast cancers (N=199) and normal breast tissue (N=17). *p=0.036. Heatmap, gene expression of indicated biomarkers in TNBC tumors (N=198) ranked by HER3 expression levels and delineating % above normal breast HER3.

## SUPPLEMENTARY FIGURES (S14-S18)

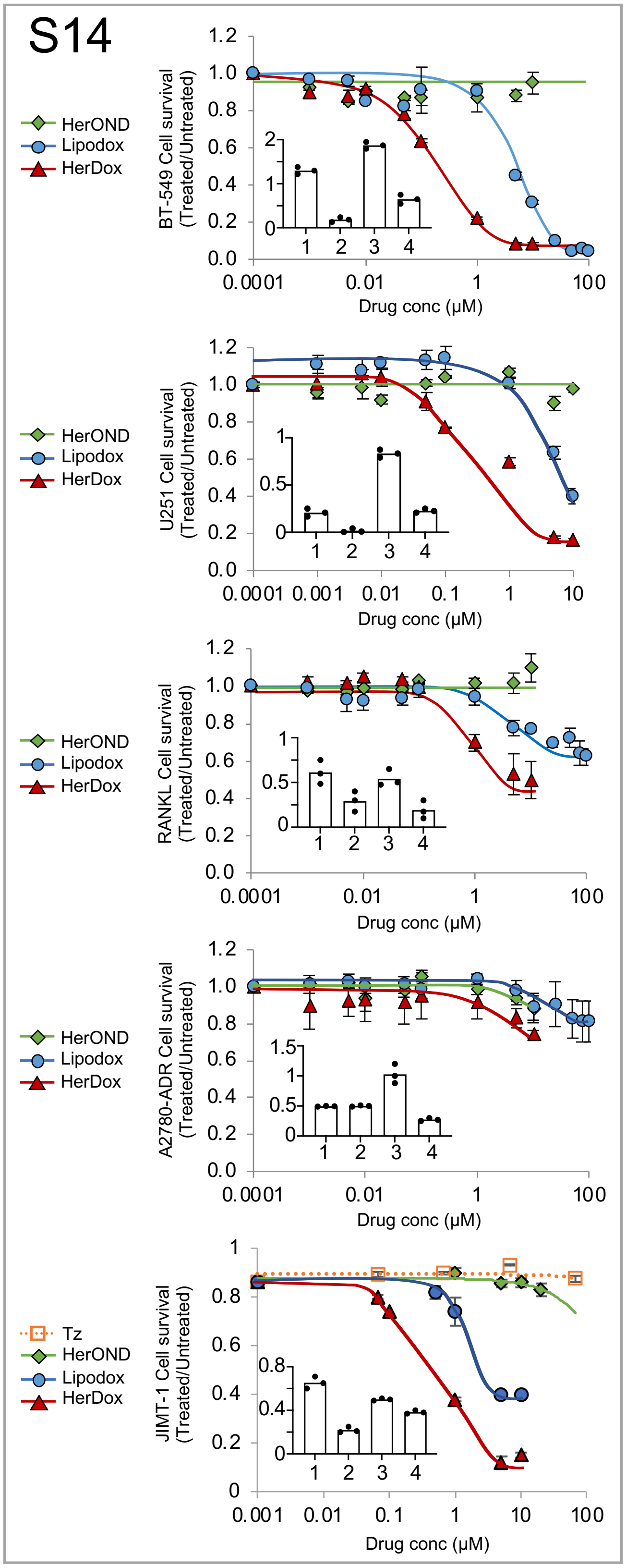


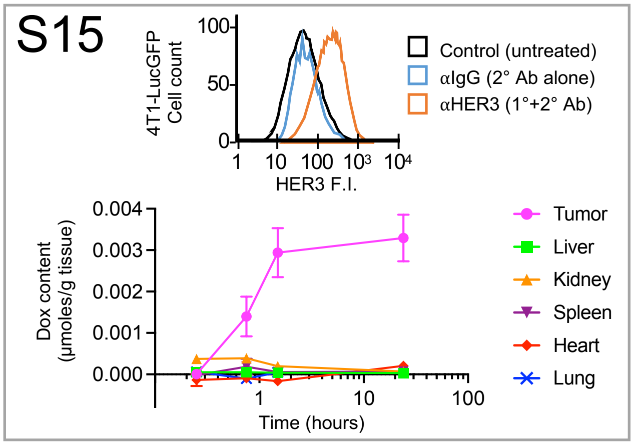


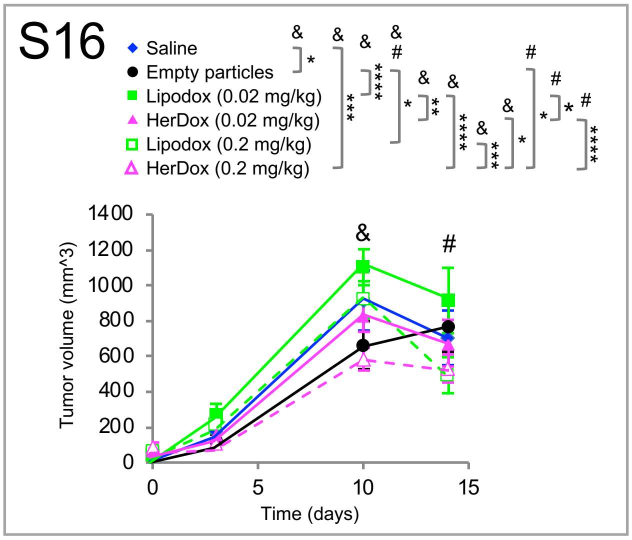


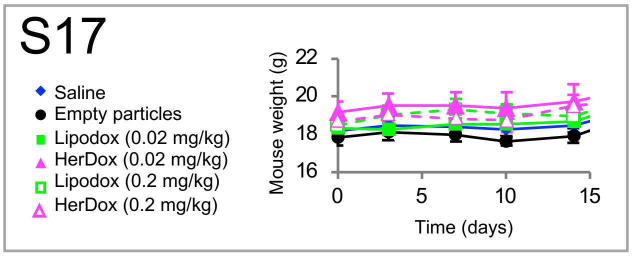


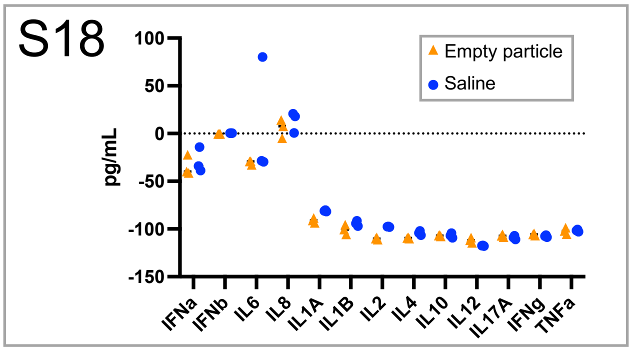

**Supplementary Figure S14.** Killing curves on human tumor lines derived from triple negative breast cancer (BT-549), glioblastoma (U251), bone-metastatic prostate cancer (RANKL), adriamycin-resistant ovarian cancer (A2780-ADR), and Herceptin/trastuzumab (Tz)-resistant HER2+ breast cancer, comparing HerDox to Lipodox empty (no Dox) NBP (HerOND), and Tz (where indicated) at 24h after treatment. Cytotoxicity was measured by crystal violet staining following standard procedures. n=3 independent experiments performed in triplicate. Insets (smaller graphs) show relative cell surface ErbB receptor levels (HER1, 2, 3, 4) acquired by cell surface ELISA. HER levels figure for JIMT1 panel is reproduced from Sims et al. (Ref #65) Supplemental Fig. S1 under Creative Commons license. Antibodies and associated methodological procedures can be found in the same publication.

**Supplementary Figure S15.** Time course of Dox distribution in indicated tissue after tail vein injection of HerDox in mice with subcutaneous HER3+ tumors. Dox content per g tissue was quantified by measuring fluorescences extrapolated against a standard curve of Dox spiked into tissue lysates. Inset (upper graph), HER3 cell surface levels on mouse TNBC tumor line measured by flow cytometry, comparing cells stained with primary and secondary antibodies (1°+2° Ab) against cells receiving secondary antibody alone (2° Ab alone) and cells receiving no antibody treatment (Control). F.I., fluorescence intensity.

**Supplementary Figure S16-S17.** Growth of 4T1 bilateral flank tumors (determined by volume measurement) in BALB/c mice during systemic treatment with indicated doses of Lipodox, HerDox, Empty (drug lacking) HPK particles (at doses equating 0.2 mg/kg HerDox) and saline. Data represent mean±SD of 5 mice per treatment group. Due to the multiple significant differences detected between treatments at specified time points (&, Day 10; #, Day 14), comparisons are shown next to the figure legend. *, $p<0.05$; **, $p<0.01$; ***, $p<0.001$; ****, $p<0.0001$. Day 0 corresponds to first day of treatment (~5 days after tumor implant). S14 summarizes mouse weights during indicated treatments. Data represent mean±SD of 5 mice per treatment group.

**Supplementary Figure S18.** Cytokine titer measurements from sera of tumor-free BALB/c mice after being inoculated with empty NBPs (at doses equating 0.2 mg/kg HerDox) or saline twice/week for 4 weeks. Samples were processed for standard ELISA and probed used a MultiAnalyte ELISArray cytokine titer kit (Qiagen).

## *SUPPLEMENTARY FIGURES (S19-S21)*

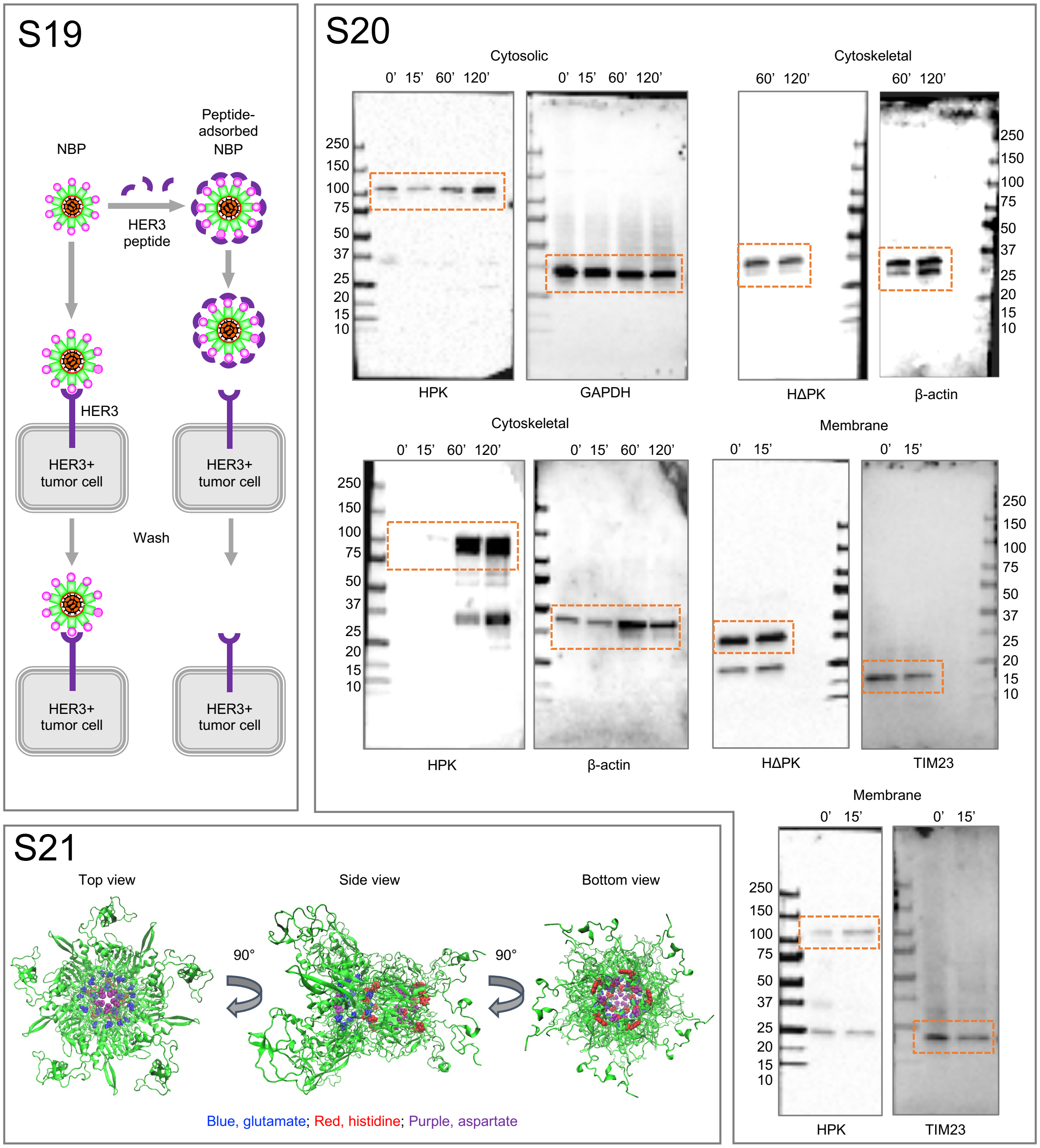

**Supplementary Figure S19.** Schematic showing approach for using HER3 peptide to block HER3 receptor binding through pre-adsorption of particles before exposure to receptor or cells.

**Supplementary Figure S20.** Full immunoblots of endosomal (membrane) and post-endosomal (cytosolic, cytoskeletal) fractions isolated from HER3+ MDA-MB-435 human tumor cells harvested and processed at the indicated time points during uptake of HPK or the PB-deleted construct, H∆PK. Membrane fractions are delineated by TIM23, cytosolic fractions are delineated by GAPDH, and cytoskeletal fractions are delineated by β-actin. Low MW (25 kDa) bands seen in the membrane fractions are likely produced by residual neuregulin bound to the cell membrane. The MW's of the standard ladder bands are shown in kDa.

**Supplementary Figure S21.** Ribbon structure of the HPK penton base domain showing protonatable-residues (blue, Glu; red, His; magenta, Asp) lining the inner barrel of the pentamer viewed from sequential rotated angles.

## ***SUPPLEMENTARY FIGURES (S22-S26)***

# S22

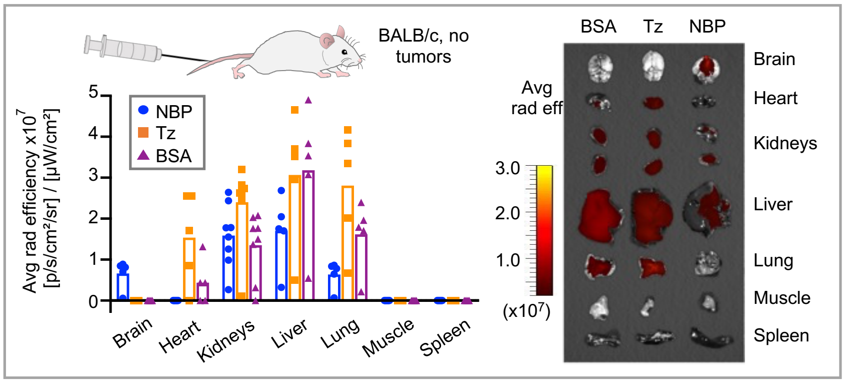


# S23

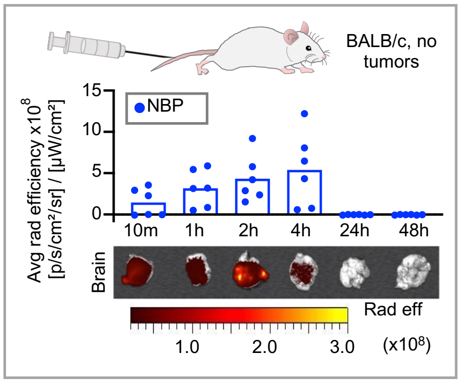


# S24

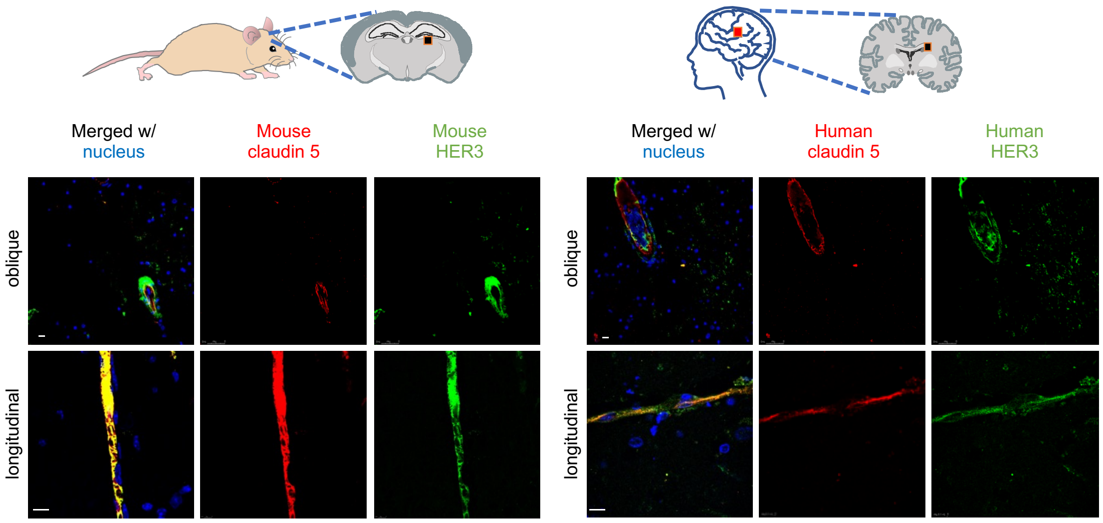


# S25

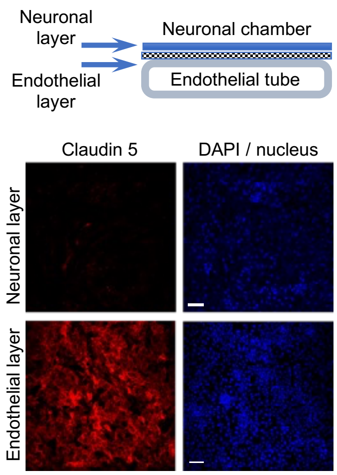


# S26

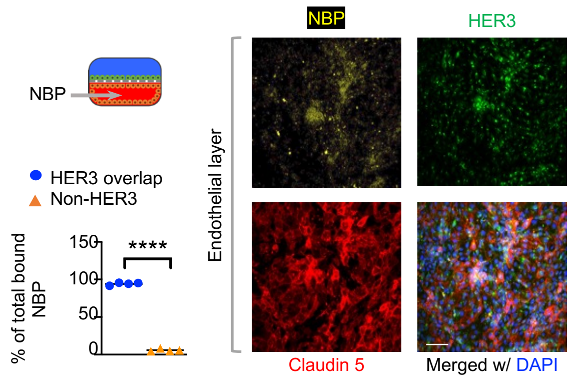

**Supplementary Figure S22.** Tissue distribution of indicated fluorescently tagged reagents at 4h after systemic delivery in tumor-free mice (n=5 mice per treatment reagent). Tissues shown were acquired from representative mice of each cohort. Tz, trastuzumab. BSA, bovine serum albumin. Data points represent the mean radiant emission collected from the indicated organ or tumor from each mouse (N=5 mice).

**Supplementary Figure S23.** Fluorescent NBP accumulation in brains harvested at indicated time points after systemic delivery of labeled HPK particles. Brains shown below graph were acquired from representative mice of each cohort. Data points represent the mean radiant emission collected from the indicated organ or tumor from each mouse (N=6 mice).

**Supplementary Figure S24.** Immunohistofluorescence of frontal cortex from non-diseased adult murine and human brains showing HER3 overlap with blood vessels within brain specimens. Specimens were obtained from female adult immunodeficient mice (6+ months) and adult human frontal cortex. Scale bar: 10 µm.

**Supplementary Figure S25.** BBB chip neuronal and endothelial chamber surfaces showing exclusivity of the claudin 5 tight junction marker at the endothelial surface. Graphic (top) shows cross sectional map of BBB chip, delineating each chamber and corresponding layer. Scale bar, 50 µm.

**Supplementary Figure S26.** Immunofluorescence detection and quantification of NBP/HER3 overlap on the (proximal) endothelial layer after 4 h of NBP flow into the endothelial micro-chamber. ****$p<0.0001$ (95% CI) using two-tailed unpaired t test (n=4). Data are presented as mean±SD. Scale bar, 50 µm.

# S27

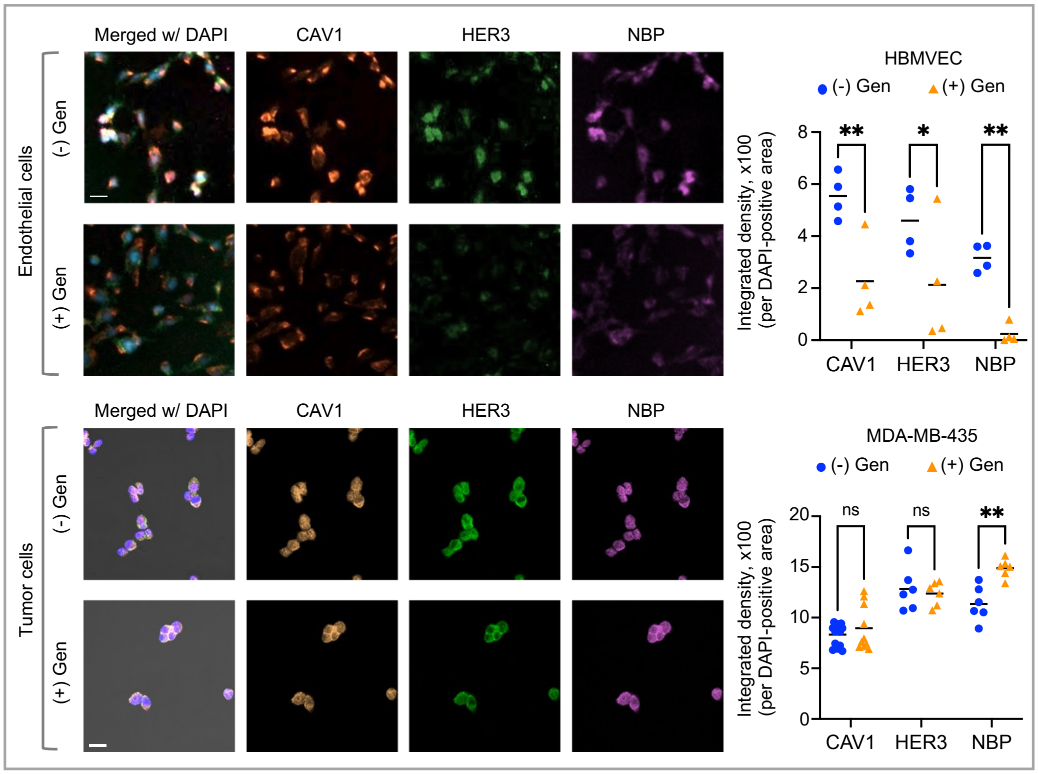

Merged w/ DAPI
CAV1
HER3
NBP
Endothelial cells
(-) Gen
(+) Gen
HBMVEC
(-) Gen
(+) Gen
Integrated density, x100 (per DAPI-positive area)
**
*
**
CAV1
HER3
NBP
Tumor cells
MDA-MB-435
ns
ns
**


# S28

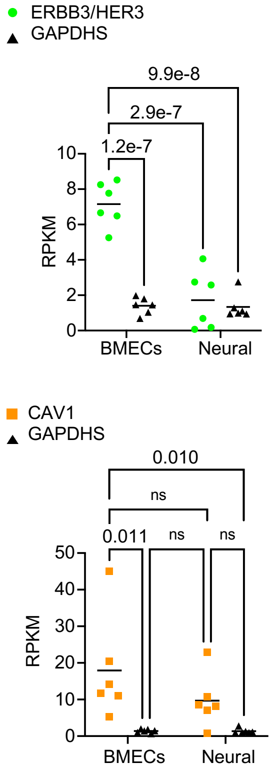

ERBB3/HER3
GAPDHS
9.9e-8
2.9e-7
1.2e-7
RPKM
BMECs
Neural
CAV1
GAPDHS
0.010
ns
0.011
ns
ns
RPKM
BMECs
Neural

**Supplementary Figure S27.** Micrographs, immunocytofluorescence of endothelial (HBMVEC) and HER3+ tumor (MDA-MB-435) cell lines at 1h after NBP uptake -/+ genistein (Gen). Scale bar: 10 μm. Graphs, quantification of indicated markers under each treatment condition in each cell line. **p=0.0026 (CAV1), *p=0.0171 (HER3), **p=0.0060 (HBMVEC NBP), **p=0.002434 (MDA-MB-435 NBP), HBMVEC: n=4 independent fields from triplicate cell treatments. MDA-MB-435: n=14 (CAV1), n=6 (HER3, NBP) independent fields from triplicate cell treatments.

**Supplementary Figure S28.** Gene transcript levels of HER3 (ErbB3) and caveolin 1 (CAV1) based on reads per kilobase per million (RPKM) from the human iPSC-derived brain microvascular endothelial cells (BMECs) and neural cells RNA-seq dataset (Acc No. GSE97324). ****p<0.0001, *p=0.0108 (BMEC CAV1 vs BMEC GAPDHS), *p=0.0104 (BMEC vs neural), n=6 independent iPSC-derived lines.

## ***SUPPLEMENTARY FIGURES (S29-S30)***

S29

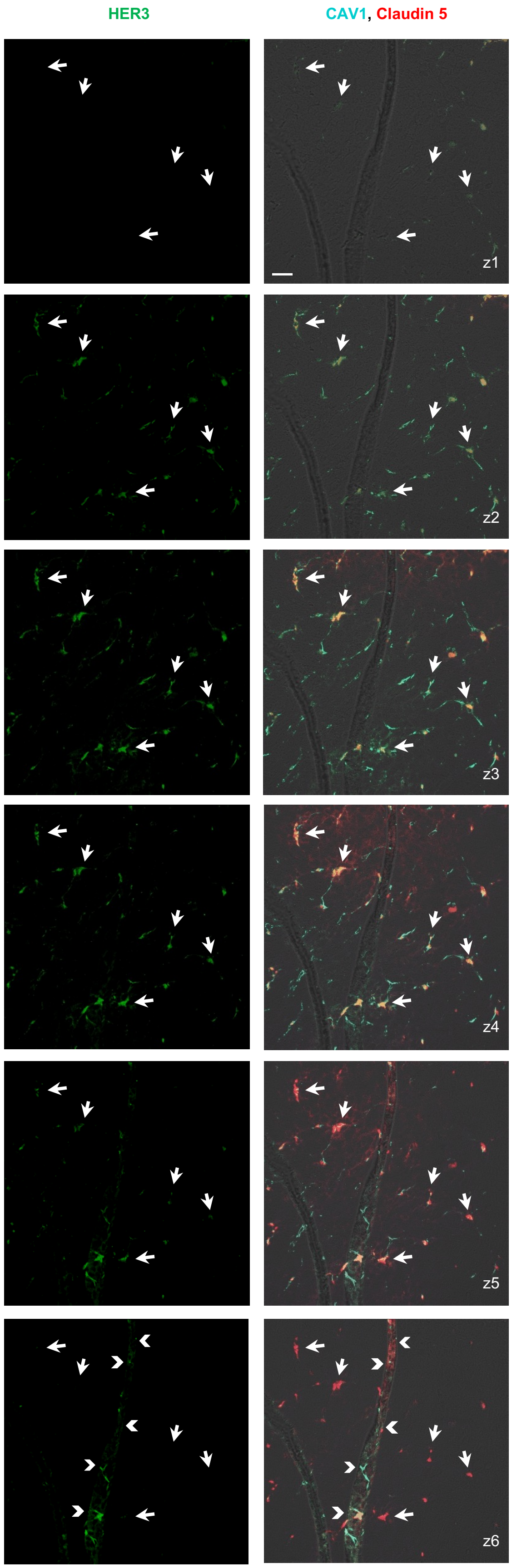


S30

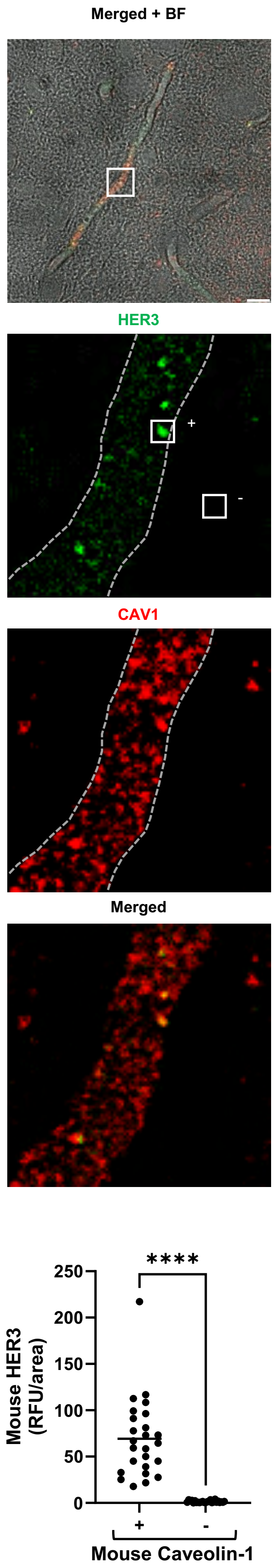

**Supplementary Figure S29.** Immunohistofluorescence stain for HER3 and caveolin 1 (CAV1) localization in relation to vasculature (claudin 5) in non-diseased mouse brain. Micrographs represent six (z1-z6) 1 μm serial visual planes along the viewing z-axis. Arrows highlight HER3-positive areas in lower row of micrographs showing same location in relation to CAV1 and claudin 5 positive areas in upper row. Scale bar, 30 μm.

**Supplementary Figure S30.** Immunohistofluorescence of frontal cortex from non-diseased adult murine brain showing blood vessels within brain specimens. Top panel, brain specimen showing immunostain for HER3 and caveolin 1 (CAV1) overlaid by brightfield image. Scale bar, 10 μm. Boxed area is enlarged in middle panels showing the channel separated immunofluorescent staining for HER3 and CAV1. Dashed lines delineate the edges of the microvessel. White squares in enlarged image delineate representative HER3 positive (+) and negative (-) areas selected to quantify the overlap between HER3 and CAV1 in the specimen (summarized by the graph). Data points in the graph represent the mean fluorescence intensities of individual regions of interest in each selected zone. Images were acquired by high resolution confocal microscopy at 100x magnification.

# S31

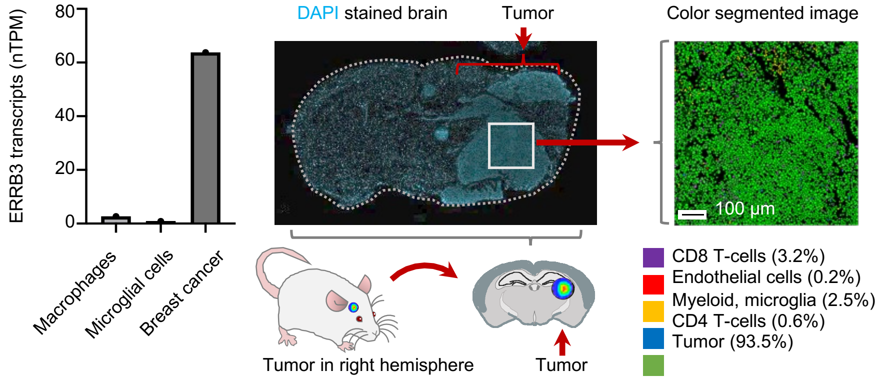
ERRB3 transcripts (nTPM)
80
60
40
20
0
Macrophages
Microglial cells
Breast cancer
DAPI stained brain
Tumor
Color segmented image
100 µm
Tumor in right hemisphere
Tumor
CD8 T-cells (3.2%)
Endothelial cells (0.2%)
Myeloid, microglia (2.5%)
CD4 T-cells (0.6%)
Tumor (93.5%)

# S32

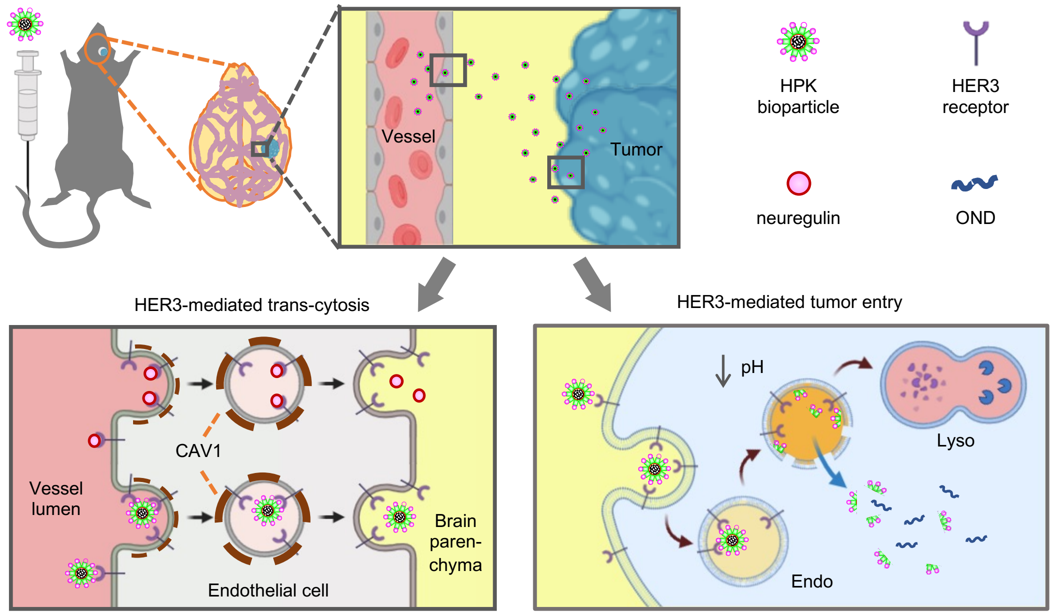
Vessel
Tumor
HPK
bioparticle
HER3
receptor
neuregulin
OND
HER3-mediated trans-cytosis
HER3-mediated tumor entry
CAV1
Vessel
lumen
Endothelial cell
Brain
paren-
chyma
pH
Lyso
Endo

# S33

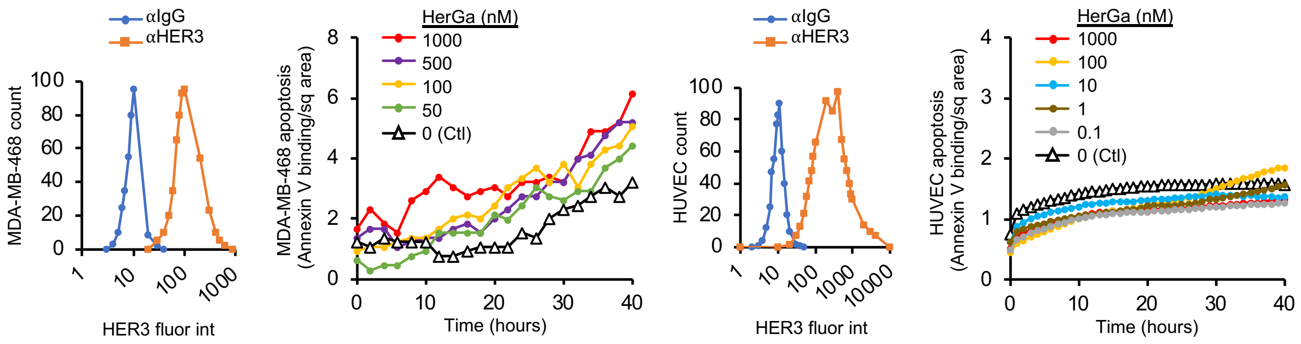
αIgG
αHER3
MDA-MB-468 count
100
80
60
40
20
0
1
10
100
1000
HER3 fluor int
HerGa (nM)
1000
500
100
50
0 (Ctl)
MDA-MB-468 apoptosis
(Annexin V binding/sq area)
8
6
4
2
0
0
10
20
30
40
Time (hours)
αIgG
αHER3
HUVEC count
100
80
60
40
20
0
1
10
100
1000
10000
HER3 fluor int
HerGa (nM)
1000
100
10
1
0.1
0 (Ctl)
HUVEC apoptosis
(Annexin V binding/sq area)
4
3
2
1
0
0
10
20
30
40
Time (hours)

**Supplementary Figure S31.** Macrophage, microglial population. Graph summarizes the RNAseq analysis of human gene expression datasets from the Human Protein Atlas comparing HER3/ERBB3 transcript levels in macrophages and microglia (single cell dataset) and 62 breast cancer cell lines. Color segmented image was acquired from 4T1 TNBC tumor shown in brain specimen of a mock (saline) treated mouse. Color segmentation was used to quantify the clustered cell populations represented in the specimen.

**Supplementary Figure S32. Summary of HER3-mediated BBB passage and tumor entry.** The HPK bioparticle exploits the native transcytosis pathway of neuregulin by binding HER3 receptors in the vessel lumen and undergoing non-acidifying transcytosis, followed by HER3-mediated endocytosis in tumor cells. Endosome (Endo) acidification triggers opening of the HPK capsomere enabling penton base -mediated membrane destabilization and release of vesicle contents, avoiding delivery to degradative lysosomes (Lyso). Graphics created using BioRender.

**Supplementary Figure S33.** Cell surface levels of HER3 and evaluation of apoptosis in human TNBC (MDA-MB-468) and human endothelial (HUVEC) cell cultures during exposure to HerGa. Flow cytometry was used to determine HER3 cell surface levels, comparing HER3 antibody ($\alpha$HER3) to secondary antibody alone ($\alpha$IgG). A live cell analysis system (IncuCyte) was used to measure binding of fluorescent Annexin V to apoptotic cells. Each data point represents an average of three wells.

## SUPPLEMENTARY FIGURES (S34-S38)

S34

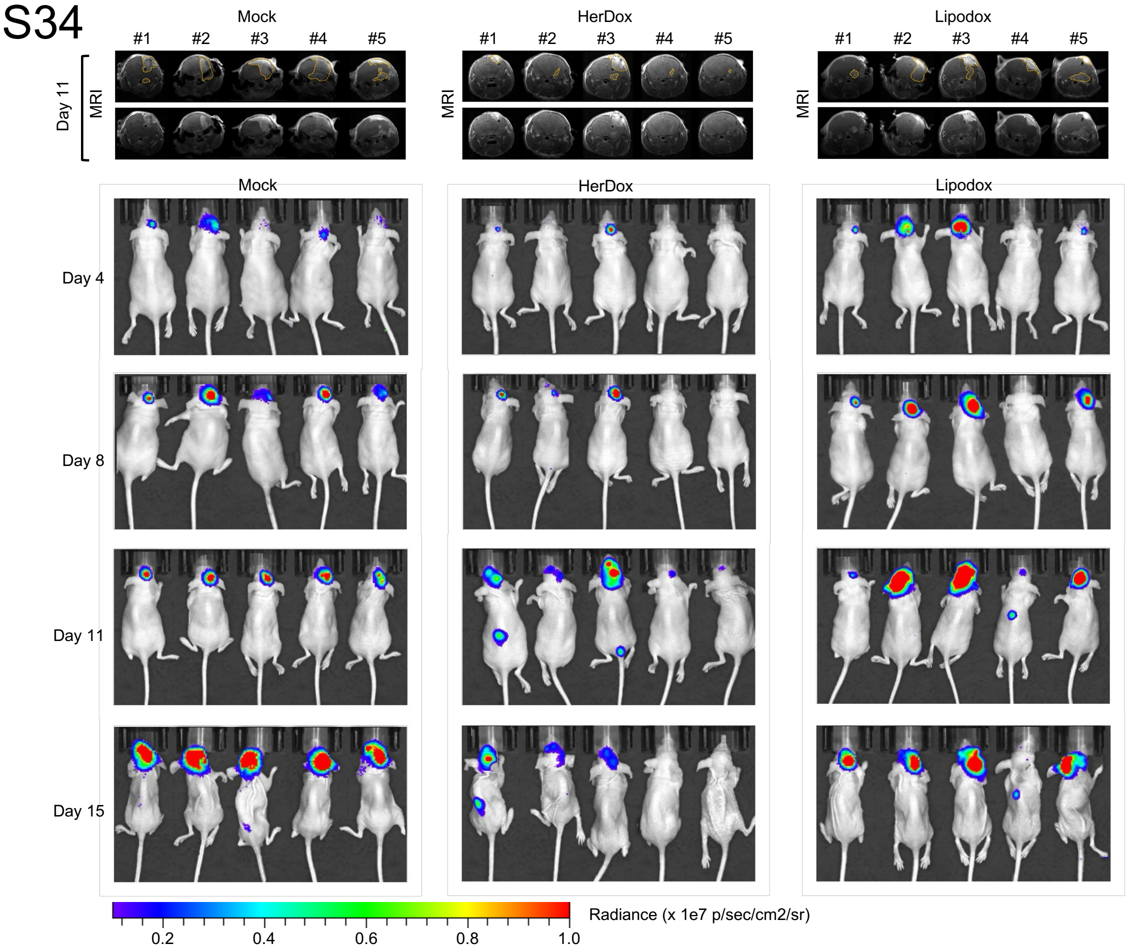


S35

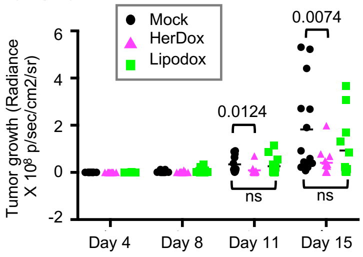


S36

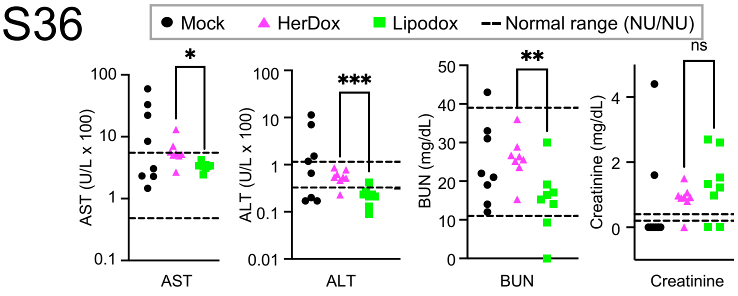


S37

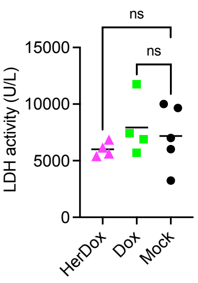


S38

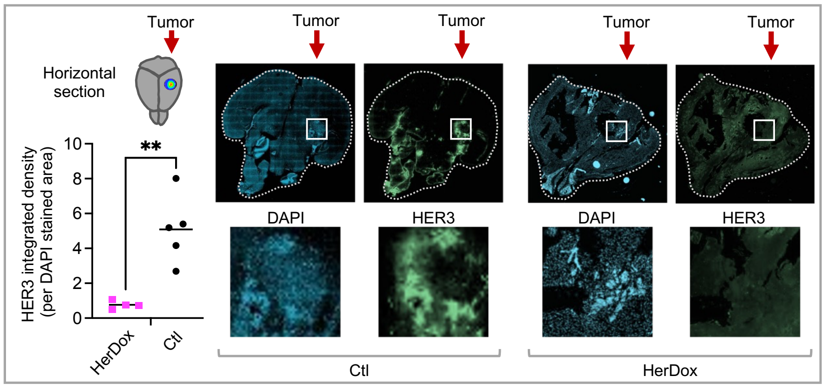

**Supplementary Figure S34.** Brain MRI (coronal view) and BLI of representative mice from each cohort bearing intracranial (IC) TNBC tumors. MRI images are shown with and without outlines of tumors, as delineated by blinded MRI core staff.

**Supplementary Figure S35.** Tumor radiance from individual mice of each cohort (n=12 each for HerDox and Lipodox, n=14 for Mock) with means indicated. Significance was assessed using 2-way ANOVA, followed by Tukey's post hoc test.

**Supplementary Figure S36-S37.** Blood analytes from treated mice. Dashed lines indicate normal ranges for each analyte per the Charles River blood chemistry report (see text for reference). Significances were determined between HerDox and Lipodox for each analyte using 2-tailed unpaired t tests in S36 and 1-way ANOVA with Bartlett's test in S37. AST, *p=0.0228 (n=8); ALT, ***p=0.0005 (n=8); BUN, **p=0.0 099 (n=8); ns, not significant (n=8).

**Supplementary Figure S38.** Immunohistofluorescence of brain specimens from HerDox-treated mice. Schematic shows location of IC tumor and orientation of brain sections shown in micrographs. Micrographs show staining for indicated biomarkers. Lower row shows enlargement of delineated area in upper row. Graph summarizes HER3 positivity per DAPI-stained area in each tumor location. **p=0.0034, HerDox (n=4) vs Ctl (n=5) determined by 2-tailed unpaired t test.

## ***SUPPLEMENTARY FIGURES (S39-S41)***

S39

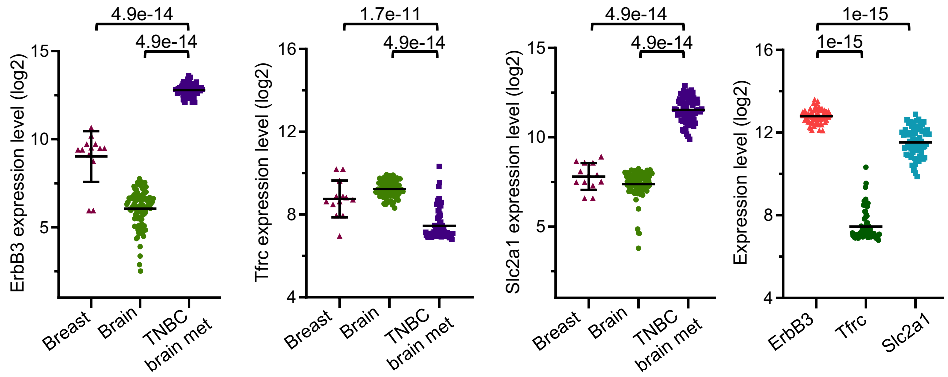


S40

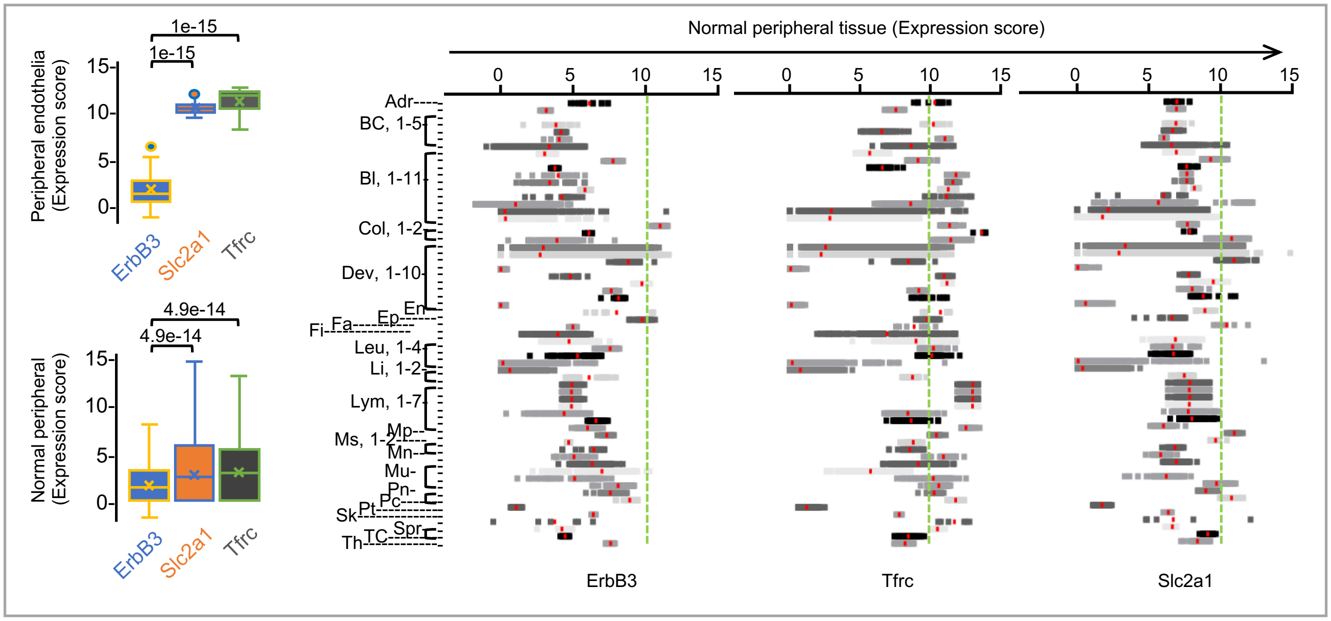


S41

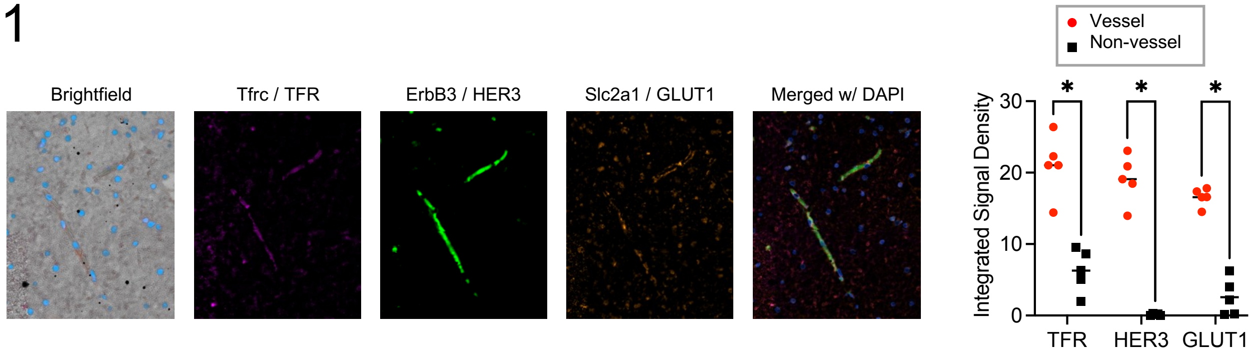

**Supplementary Figure S39.** Human gene expression analysis of HER3 (ErbB3), transferrin receptor (TFRC), and glucose receptor (SLC2A1) from brain metastatic TNBC (GSE76714), normal breast (Roth), and normal brain (GSE3594) datasets. ****p<0.0001.

**Supplementary Figure S40.** Left graphs, human gene expression analysis of ErbB3, SLC2A1, and TFCR from peripheral endothelial tissue (N=38 per group) and peripheral non-tumor tissues (N=31,408 per group). ****p<0.0001. Lower left graph summarizes itemized expression scores shown on the right, which represent human peripheral (non-brain) non-tumor tissues comparing ErbB3, TFCR, and SLC2A1 gene expression. Red line in each category indicates mean. Dashed vertical line delineates high expression threshold. Each y-axis tick mark represents a separate database. Multiple databases within the same category are enumerated. Sources and N of each database are listed in the *Methods*. Adr, adrenal; BC, B cell; Bl, blood; Col, colon; Dev, developmental; En, endothelial; Ep, epithelial; Fa, fallopian tube; Fi, fibroblasts; Leu, leukocytes; Li, liver; Lym, lymphocytes; Mp, macrophage; Ms, mesenchymal; Mn, monocytes; Mu, muscle; Pn, pancreatic; Pc, placenta; Pt, platelets; Sk, skeletal; Spr, spermatogonia; TC, T cells; Th, thymus.

**Supplementary Figure S41.** Micrographs show immunohistofluorescence of non-diseased adult human brain showing staining of TFR, HER3, and GLUT1 in relation to vessel structures within brain specimens. Graph summarizes relative signal densities of each biomarker at vessel vs non-vessel areas. *p=0.000237 (TFR, n=5); *p=0.000002 (HER3, n=5); *p=0.000005 (GLUT1, n=5).

***SUPPLEMENTARY FIGURES (S42-S45)***

S42

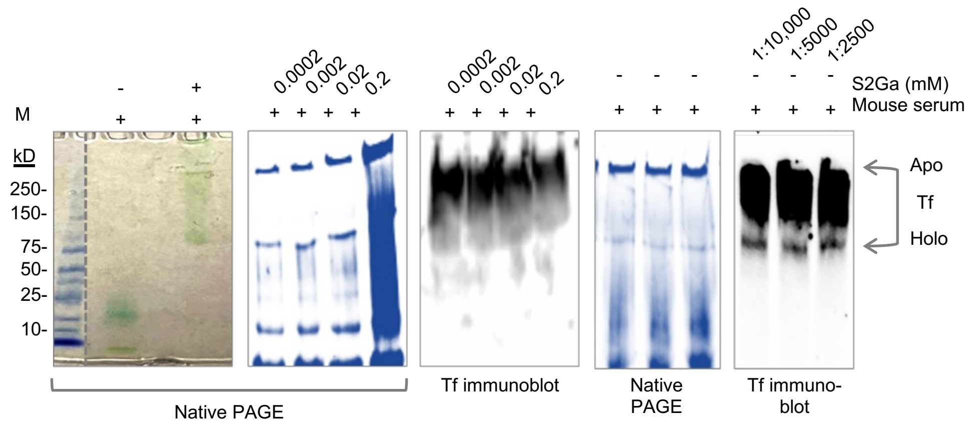


S43

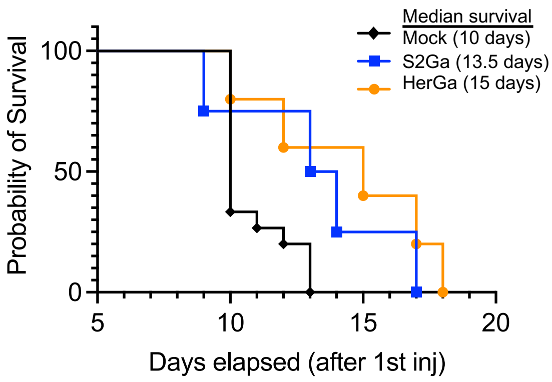


S44

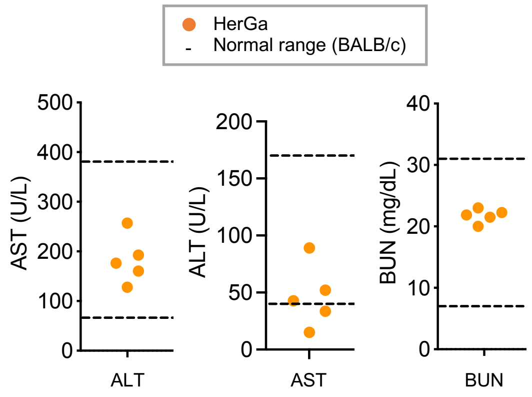


S45

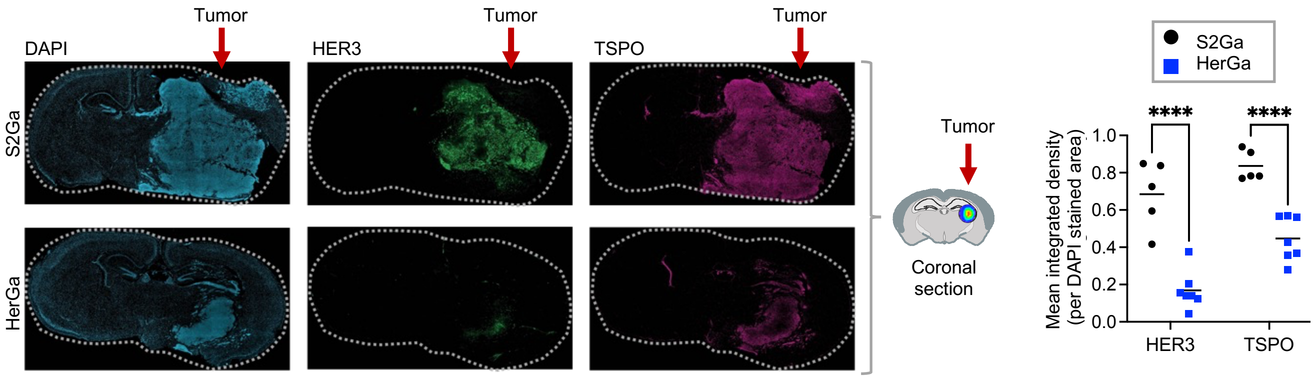

**Supplementary Fig. S42.** S2Ga binding to serum transferrin. Left to right: Left, Native PAGE of S2Ga (25 nmoles/lane) -/+ mouse serum (10 µL/lane). S2Ga and mouse serum were incubated for 30 min at RT before loading on gel. M, pre-stained molecular weight marker. S2Ga can be visualized on unstained gel by greenish pigmentation. Second gel shows native PAGE of mouse serum at indicated concentrations (10 µL/lane) on a trihalo-modified stain-free gel (BioRad) followed by UV visualization. Image is pseudo-colored to show protein in blue. Middle, Immunoblot of native PAGE is shown using anti-transferrin antibody (SinoBiological) at 1:10,000 dilution. Right two panels show native PAGE of mouse serum alone and immunoblotted using anti-transferrin antibody (SinoBiological) at indicated dilutions. Tf, transferrin. Arrows point to molecular weight mobilities of apo (Apo) and holo (Holo) transferrin.

**Supplementary Fig. S43.** Kaplan-Meier survival probability curves for treated mice.

**Supplementary Fig. S44.** Blood analytes from treated mice. Dashed lines indicate normal ranges for each analyte per the Charles River blood chemistry report (see text for reference).

**Supplementary Fig. S45.** Immunohistofluorescence of brain specimens from HerGa and S2Ga treated mice. Schematic shows location of IC tumor and orientation of brain sections shown in micrographs. Micrographs show staining for indicated biomarkers. Graph summarizes HER3 and TSPO positivity per DAPI-stained area in each tumor location. ****$p<0.0001$ (Ctl, n=5; HerGa, n=7).
