# Supplementary material for: Systemic HER3 ligand-mimicking nanobioparticles enter the brain and reduce intracranial tumour growth
Source: Nat Nanotechnol. 2025 Feb 21;20(5):683–96. doi: 10.1038/s41565-025-01867-7 (PMC12095042; doi:10.1038/s41565-025-01867-7)
Supplement: Supplementary file 2 [file 41565_2025_1867_MOESM2_ESM.pdf]

nature portfolio

Corresponding author(s): Lali K Medina-Kauwe

Last updated by author(s): Sep 11, 2024

Reporting Summary

Nature Portfolio wishes to improve the reproducibility of the work that we publish. This form provides structure for consistency and transparency in reporting. For further information on Nature Portfolio policies, see our Editorial Policies and the Editorial Policy Checklist.

Statistics

For all statistical analyses, confirm that the following items are present in the figure legend, table legend, main text, or Methods section.

n/a	Confirmed	
☐	☒	The exact sample size (*n*) for each experimental group/condition, given as a discrete number and unit of measurement
☐	☒	A statement on whether measurements were taken from distinct samples or whether the same sample was measured repeatedly
☐	☒	The statistical test(s) used AND whether they are one- or two-sided<br>*Only common tests should be described solely by name; describe more complex techniques in the Methods section.*
☐	☒	A description of all covariates tested
☐	☒	A description of any assumptions or corrections, such as tests of normality and adjustment for multiple comparisons
☐	☒	A full description of the statistical parameters including central tendency (e.g. means) or other basic estimates (e.g. regression coefficient) AND variation (e.g. standard deviation) or associated estimates of uncertainty (e.g. confidence intervals)
☐	☒	For null hypothesis testing, the test statistic (e.g. *F*, *t*, *r*) with confidence intervals, effect sizes, degrees of freedom and *P* value noted<br>*Give P values as exact values whenever suitable.*
☒	☐	For Bayesian analysis, information on the choice of priors and Markov chain Monte Carlo settings
☒	☐	For hierarchical and complex designs, identification of the appropriate level for tests and full reporting of outcomes
☒	☐	Estimates of effect sizes (e.g. Cohen's *d*, Pearson's *r*), indicating how they were calculated

Our web collection on statistics for biologists contains articles on many of the points above.

Software and code

Policy information about availability of computer code

Data collection

• The AMBER simulation (version 18) package was used for computational modeling.
• Spectramax M2 spectrophotometer SoftMax Pro Software (version 7.x) was used for measuring absorbances and fluorescences.
• Zetasizer Software version 7.01 was used for dynamic light scattering.
• MetaXpress Version 6.7.2.290 image editing software and CellReporterXpress 2.9.4.19394 imaging and analysis software were used for image acquisition and analysis on the Molecular Devices ImageXpress® Pico Automated Cell Imaging System.
• Aynix OS Version 2.0 was used for the Orflo Technologies Moxi GO benchtop cell analyzer
• Leica Application Suite X (LAS X) 3.3.0.16799 was used for the Leica SPE laser-scanning confocal microscope
• NIS Elements version 5.42.01 and IMARIS version 10.2.0 were used for image acquisition and analysis of BBB chips using a Nikon A1R confocal microscope.
• PerkinElmer IVIS Lumina Spectrum LivingImageTM software version 4.8.0.
• MRI, Bruker Paravision 5.1 software

Data analysis

• GraphPad Prism 10.3.1 was used for general data analysis.
• The R2 Genomics Analysis and Visualization Platform (http://r2.amc.nl) was used for gene expression data analysis.

For manuscripts utilizing custom algorithms or software that are central to the research but not yet described in published literature, software must be made available to editors and reviewers. We strongly encourage code deposition in a community repository (e.g. GitHub). See the Nature Portfolio guidelines for submitting code & software for further information.

Data

Policy information about availability of data

All manuscripts must include a data availability statement. This statement should provide the following information, where applicable:

- Accession codes, unique identifiers, or web links for publicly available datasets
- A description of any restrictions on data availability
- For clinical datasets or third party data, please ensure that the statement adheres to our policy

Databases/datasets analyzed in the current study are publicly accessible through the following accession codes: brain metastatic TNBC (GSE76714), normal breast (GSE7307), normal brain (GSE3594) RNA-seq datasets; iPSC-derived brain microvascular endothelial cells and neural cells RNA-seq dataset (GSE97324).

Research involving human participants, their data, or biological material

Policy information about studies with human participants or human data. See also policy information about sex, gender (identity/presentation), and sexual orientation and race, ethnicity and racism.

Reporting on sex and gender	*The focus of this research is on triple-negative breast cancer hence the findings apply to only one sex.*
Reporting on race, ethnicity, or other socially relevant groupings	*This study does not include race, ethnicity, or other socially relevant groupings.*
Population characteristics	*Biological material (cell lines and primary tumor cells) were originally obtained from women with breast tumors. Patient age range is 31-72 years.*
Recruitment	*Primary tumor cells were obtained by patient consent under IRB protocol 29973.*
Ethics oversight	*Cedars-Sinai Medical Center Institutional Review Board (IRB)*

Note that full information on the approval of the study protocol must also be provided in the manuscript.

Field-specific reporting

Please select the one below that is the best fit for your research. If you are not sure, read the appropriate sections before making your selection.

☒ Life sciences ☐ Behavioural & social sciences ☐ Ecological, evolutionary & environmental sciences

For a reference copy of the document with all sections, see nature.com/documents/nr-reporting-summary-flat.pdf

Life sciences study design

All studies must disclose on these points even when the disclosure is negative.

Sample size	*In vitro sample sizes were determined based on power analysis of previous similar data* to yield 90% power comparing treatment groups in a two-factor ANOVA at the 0.05 significance level. In vivo sample sizes involving tumor growth were based on power analysis of previous tumor growth studies* to achieve at least 90% power at the 0.05 significance level in a repeated-measures ANOVA.<br>*Nucleic acids research 2019, 47 (21), 11020-11043; Journal of Controlled Release 2018, 271, 127-138*
Data exclusions	*No data were excluded from the analyses in these studies.*
Replication	*The data reported reflect successful reproduction of experiments that were repeated at least three times with a minimum of three replicates per treatment group unless otherwise indicated based on power analysis of previous similar studies.*
Randomization	*For evaluating therapeutic efficacy, mice were randomized at tumor establishment into separate treatment groups with each group comprising a similar set of large, medium, and small tumors if tumor sizes differed from one another based on detection method (palpability, volume measurement and/or bioluminescence). For in vitro experiments, cells prepared for treatment were suspended in a volume of media so that repeating aliquots contained equivalent cell numbers, which comprised the replicate samples for experimental groups.*
Blinding	*To ensure rigor, all measurements were collected in blinded fashion with cohort identities unknown to the researcher collecting them.*

Behavioural & social sciences study design

All studies must disclose on these points even when the disclosure is negative.

Study description	*Briefly describe the study type including whether data are quantitative, qualitative, or mixed-methods (e.g. qualitative cross-sectional,*

Study description	*quantitative experimental, mixed-methods case study).*
Research sample	*State the research sample (e.g. Harvard university undergraduates, villagers in rural India) and provide relevant demographic information (e.g. age, sex) and indicate whether the sample is representative. Provide a rationale for the study sample chosen. For studies involving existing datasets, please describe the dataset and source.*
Sampling strategy	*Describe the sampling procedure (e.g. random, snowball, stratified, convenience). Describe the statistical methods that were used to predetermine sample size OR if no sample-size calculation was performed, describe how sample sizes were chosen and provide a rationale for why these sample sizes are sufficient. For qualitative data, please indicate whether data saturation was considered, and what criteria were used to decide that no further sampling was needed.*
Data collection	*Provide details about the data collection procedure, including the instruments or devices used to record the data (e.g. pen and paper, computer, eye tracker, video or audio equipment) whether anyone was present besides the participant(s) and the researcher, and whether the researcher was blind to experimental condition and/or the study hypothesis during data collection.*
Timing	*Indicate the start and stop dates of data collection. If there is a gap between collection periods, state the dates for each sample cohort.*
Data exclusions	*If no data were excluded from the analyses, state so OR if data were excluded, provide the exact number of exclusions and the rationale behind them, indicating whether exclusion criteria were pre-established.*
Non-participation	*State how many participants dropped out/declined participation and the reason(s) given OR provide response rate OR state that no participants dropped out/declined participation.*
Randomization	*If participants were not allocated into experimental groups, state so OR describe how participants were allocated to groups, and if allocation was not random, describe how covariates were controlled.*

Ecological, evolutionary & environmental sciences study design

All studies must disclose on these points even when the disclosure is negative.

Study description	*Briefly describe the study. For quantitative data include treatment factors and interactions, design structure (e.g. factorial, nested, hierarchical), nature and number of experimental units and replicates.*
Research sample	*Describe the research sample (e.g. a group of tagged Passer domesticus, all Stenocereus thurberi within Organ Pipe Cactus National Monument), and provide a rationale for the sample choice. When relevant, describe the organism taxa, source, sex, age range and any manipulations. State what population the sample is meant to represent when applicable. For studies involving existing datasets, describe the data and its source.*
Sampling strategy	*Note the sampling procedure. Describe the statistical methods that were used to predetermine sample size OR if no sample-size calculation was performed, describe how sample sizes were chosen and provide a rationale for why these sample sizes are sufficient.*
Data collection	*Describe the data collection procedure, including who recorded the data and how.*
Timing and spatial scale	*Indicate the start and stop dates of data collection, noting the frequency and periodicity of sampling and providing a rationale for these choices. If there is a gap between collection periods, state the dates for each sample cohort. Specify the spatial scale from which the data are taken*
Data exclusions	*If no data were excluded from the analyses, state so OR if data were excluded, describe the exclusions and the rationale behind them, indicating whether exclusion criteria were pre-established.*
Reproducibility	*Describe the measures taken to verify the reproducibility of experimental findings. For each experiment, note whether any attempts to repeat the experiment failed OR state that all attempts to repeat the experiment were successful.*
Randomization	*Describe how samples/organisms/participants were allocated into groups. If allocation was not random, describe how covariates were controlled. If this is not relevant to your study, explain why.*
Blinding	*Describe the extent of blinding used during data acquisition and analysis. If blinding was not possible, describe why OR explain why blinding was not relevant to your study.*

Did the study involve field work? ☐ Yes ☐ No

Field work, collection and transport

Field conditions	*Describe the study conditions for field work, providing relevant parameters (e.g. temperature, rainfall).*
Location	*State the location of the sampling or experiment, providing relevant parameters (e.g. latitude and longitude, elevation, water depth).*
Access & import/export	*Describe the efforts you have made to access habitats and to collect and import/export your samples in a responsible manner and in*

Access & import/export | compliance with local, national and international laws, noting any permits that were obtained (give the name of the issuing authority, the date of issue, and any identifying information).

Disturbance | Describe any disturbance caused by the study and how it was minimized.

Reporting for specific materials, systems and methods

We require information from authors about some types of materials, experimental systems and methods used in many studies. Here, indicate whether each material, system or method listed is relevant to your study. If you are not sure if a list item applies to your research, read the appropriate section before selecting a response.

Materials & experimental systems

n/a	Involved in the study	
☐	☒	Antibodies
☐	☒	Eukaryotic cell lines
☒	☐	Palaeontology and archaeology
☐	☒	Animals and other organisms
☒	☐	Clinical data
☒	☐	Dual use research of concern
☒	☐	Plants

Methods

n/a	Involved in the study	
☒	☐	ChIP-seq
☒	☐	Flow cytometry
☒	☐	MRI-based neuroimaging

Antibodies

Antibodies used

- Thermo Fisher Scientific ERBB3 monoclonal antibody, H3.105.5 (Ab105), lot # RF2226781E, 1:500
- R&D Systems sheep anti-mouse ERBB3/HER3 polyclonal antibody (AF4518), lot # CBAZ0121051, 1:400
- Invitrogen F(ab')2-goat anti-mouse IgG (H+L) cross-adsorbed secondary antibody Alexa Fluor™ 488 conjugate (A-11017), lot # 2048172, 1:500
- Abcam donkey anti-sheep IgG H&L Alexa Fluor® 488 conjugate (ab150177), lot # GR3386562-1, 1:1000
- Abcam donkey anti-sheep IgG H&L Alexa Fluor® 647 conjugate (ab150179), lot # GR3285522-1, 1:1000
- Cell Signaling HER3/ErbB3 XP® rabbit mAb #12708 (D22C5), lot # 4: IHC (1:250), ICC (1:200)
- Cell Signaling goat anti-rabbit IgG (H+L), F(ab')2 fragment Alexa Fluor® 488 conjugate (4412), lot # 23, 1:1000
- Abcam anti-RAB7 antibody, Rab7-117 (ab50533), lot # GR3213219-1, 1:50
- Abcam anti-Early Endosome Antigen 1 (EEA1) antibody, (ab206860), lot # GR3231042-7, 1:100
- R&D Systems anti-GAPDH antibody, 686613 (MAB5718), lot # CEZS0319111, 1:10,000
- R&D Systems anti-beta-actin antibody, 937215 (MAB8929), lot # CJQV0321041, 1:5000
- BD Biosciences anti-TIM23 antibody, 32 (611222), lot # 9087758, 1:5000
- Abcam Goat Anti-Rabbit IgG H&L HRP conjugate (ab6721), lot # 1042368-7, 1:3000
- Abcam Goat Anti-Mouse IgG H&L HRP conjugate (ab6789), lot # GR3335361-9, 1:2000
- Invitrogen claudin-5 monoclonal antibody, 4C3C2 (35-2500), lot # RH235294, 1:50
- Abcam Anti-Adenovirus Type 5 antibody (ab6982), lot # GR127734-10, 1:100
- Qiagen Anti RGS·His Antibody (34610), lot # 157041658, 1:100
- Abcam Anti-Caveolin-1 antibody (ab2910), lot # 1061095-2, 1:500

Validation

[From the Supplemental Materials, Section S1.4]: The Thermo Fisher Scientific H3.105.5 (Ab105) mouse antibody recognizing human and mouse HER3 was used at 1:500 dilution following the indicated methodological procedures as described on patient-derived breast tumor specimens (Fig. 2D), human tumor cells for intracellular trafficking studies (Fig. 2E), and isolated human brain microvessel endothelial cells (Fig. 4H). Antibody specificity was validated by siRNA-mediated knock-down of HER3 transcript expression as described previously,* which reduced immunorecognition of HER3 to undetectable levels on human brain microvessel endothelial cells (Fig. 4H). Secondary antibody alone (Invitrogen goat anti-mouse IgG secondary antibody Alexa Fluor® 488 conjugate A-11017 at 1:500 dilution) lacked detectable immunoreactivity in the absence of the primary HER3 antibody on the same cells (Fig. 4H). The R&D Systems sheep anti-mouse HER3 antibody (AF4518) which also cross reacts with human HER3 was used at 1:400 dilution on mouse and human tumor and non-tumor cell lines (Fig. 1L; Supplementary Figs. S15, S24, S30), TMAs (Supplementary Figs. S10-S12), BBB chips (Fig. 4 A, B, D, E), and mouse tissue specimens undergoing co-immunostaining for CAV1 (Fig. 4I-J, 5C-D; Supplementary Fig. S26, S27) or other biomarkers (Supplementary Figs. S35, S38, S42). HER3 specificity was validated by siRNA-mediated knock-down of HER3 transcript expression as described previously,1 which reduced immunorecognition of HER3 to undetectable levels on human tumor cells (Fig. 2A). Secondary antibody alone (Donkey anti-sheep IgG Alexa Fluor® 488 conjugate ab150177 or Alexa Fluor® 647 conjugate ab150179) lacked detectable immunoreactivity in the absence of the primary HER3 antibody on mouse tumor and non-tumor cells (Fig. 1L; Supplementary Fig. S15) and human tumor and endothelial lines (Supplementary Fig. S30). Immunohistological staining in brain specimens were independently confirmed with a rabbit antibody recognizing both mouse and human HER3 (Cell Signaling D22C5) and a goat anti-rabbit IgG secondary antibody (Cell Signaling Alexa Fluor® 488 conjugate #4412) (Fig. 3 G, I, J; Supplementary Fig. S22). Secondary antibody alone lacked detectable immunoreactivity in the absence of the primary HER3 antibody on human tumor cell lines (Supplementary Fig. S9).

* Alonso-Valenteen, F., Pacheco, S., Srinivas, D., Rentsendorj, A., Chu, D., Lubow, J., Sims, J., Miao, T., Mikhael, S., Hwang, J. Y., Abrol, R., Medina Kauwe, L. K., HER3-targeted protein chimera forms endosomolytic capsomeres and self-assembles into stealth nucleocapsids for systemic tumor homing of RNA interference in vivo. Nucleic acids research 2019, 47 (21), 11020-11043. PMC6868389

All other primary antibodies:

• Abcam anti-RAB7 antibody, Rab7-117 (ab50533), lot # GR3213219-1, 1:50
Validated for ICC in human cell lines at the dilution used per the antibody profiling data provided on the manufacturer's website.

• Abcam anti-Early Endosome Antigen 1 (EEA1) antibody, (ab206860), lot # GR3231042-7, 1:100
Validated for ICC in human cell lines at the dilution used per the antibody profiling data provided on the manufacturer's website.

• R&D Systems anti-GAPDH antibody, 686613 (MAB5718), lot # CEZS0319111, 1:10,000
Validated for WB of human and mouse species at the dilution used per the antibody profiling data provided on the manufacturer's website.

• R&D Systems anti-beta-actin antibody, 937215 (MAB8929), lot # CJQV0321041, 1:5000
Validated for WB of human and mouse species at the dilution used per the antibody profiling data provided on the manufacturer's website.

• BD Biosciences anti-TIM23 antibody, 32 (611222), lot # 9087758, 1:5000
Validated for WB of human species at the dilution used per the antibody profiling data provided on the manufacturer's website.

• Invitrogen claudin-5 monoclonal antibody, 4C3C2 (35-2500), lot # RH235294, 1:50
Validated for IHC/ICC/IF of human and mouse species at the dilution used per the antibody profiling data provided on the manufacturer's website.

• Abcam Anti-Adenovirus Type 5 antibody (ab6982), lot # GR127734-10, 1:100
Validated for WB, ICC/IF, ELISA of human adenovirus 5 samples at the dilution used per the antibody profiling data provided on the manufacturer's website.

• Qiagen RGS·His Antibody (34610), lot # 157041658, 1:100

Validated for WB, IHC/ICC, ELISA of RGS-6xHis tagged protein at the dilution used per the antibody profiling data provided on the manufacturer's website.

• Abcam Anti-Caveolin-1 antibody (ab2910), lot # 1061095-2, 1:500
Validated for ICC/IF of human and mouse species at the dilution used per the antibody profiling data provided on the manufacturer's website.

Eukaryotic cell lines

Policy information about cell lines and Sex and Gender in Research

Cell line source(s): The following cell lines were originally obtained from the American Type Culture Collection (ATCC): a female mouse triple-negative mammary tumor line (4T1), a female human breast tumor-derived melanoma line (MDA-MB-435), female human triple-negative breast cancer lines (BT-549, MDA-MB-468, MDA-MB-231), female human glioma line (LN229), a mouse embryo fibroblast line (NIH-3T3), and human endothelial cell line (HUVEC). Human brain microvascular endothelial cells (HBMVECs) came from Cell Systems. The male human U251 glioblastoma line was originally obtained from the NCI cell line repository. The 4T1-LucGFP reporter line was generated in house by stable lentiviral transduction of a construct (kindly provided by Dr. M. Kahn, University of Southern California) expressing luciferase and IRES-driven GFP and validated for expression of HER3 on the cell surface. The female human HER2+ breast cancer line (JIMT-1) was kindly provided by the lab of Dr. J. Ljubimova, Cedars-Sinai Medical Center. The MDA-MB-231(+) and MDA-MB-231(-) sub-lines were isolated as naturally evolved HER3 positive and HER3 negative TNBC lines (based on cell surface HER3 levels). Dr. Dr. X. Cui, Cedars-Sinai Medical Center, kindly provided the original HER3 negative MDA-MB-231 line and the brain-passaged MDA-MB-435 sub-line (MDA-MB-435-BR4). The human bone-metastatic prostate cancer line (RANKL) was kindly provided by the lab of Dr. L. Chung, Cedars-Sinai Medical Center. The human ovarian cancer line (A2780-ADR) was obtained from Sigma-Aldrich.

Authentication: All cell lines were authenticated by STR profiling using an external authentication service (IDEXX BioAnalytics CellCheck). Through this testing, the MDA-MB-435 Br4 line was identified as originating from a non-small cell lung cancer. Additional authentication by the original vendors included CO1 barcoding (4T1, LN229), G6PD type A isozyme analysis (MDA-MB-468), and karyotyping (U251, HUVEC).

Mycoplasma contamination: All cell lines were tested for mycoplasma contamination using an external service (IDEXX BioAnalytics STAT-Myco™ PCR testing). Only mycoplasma-free cells were used in these studies.

Commonly misidentified lines (See ICLAC register): MDA-MB-435 melanoma cells are commonly misidentified as breast cancer cells. These cells express high cell surface HER3 and were used as a comparison against the breast cancer and other cell lines.

Palaeontology and Archaeology

Specimen provenance: Provide provenance information for specimens and describe permits that were obtained for the work (including the name of the issuing authority, the date of issue, and any identifying information). Permits should encompass collection and, where applicable, export.

Specimen deposition: Indicate where the specimens have been deposited to permit free access by other researchers.

Dating methods	*If new dates are provided, describe how they were obtained (e.g. collection, storage, sample pretreatment and measurement), where they were obtained (i.e. lab name), the calibration program and the protocol for quality assurance OR state that no new dates are provided.*

☐ Tick this box to confirm that the raw and calibrated dates are available in the paper or in Supplementary Information.

Ethics oversight	*Identify the organization(s) that approved or provided guidance on the study protocol, OR state that no ethical approval or guidance was required and explain why not.*

Note that full information on the approval of the study protocol must also be provided in the manuscript.

Animals and other research organisms

Policy information about studies involving animals; ARRIVE guidelines recommended for reporting animal research, and Sex and Gender in Research

Laboratory animals	*Mouse (Mus musculus), BALB/c, female, 6 week*<br>*Mouse (Mus musculus), NU/NU, female, 4-6 week*
Wild animals	*The study did not involve wild animals*
Reporting on sex	*Findings apply to only one sex.*
Field-collected samples	*The study did not involve collecting samples from the field.*
Ethics oversight	*The Cedars-Sinai Institutional Animal Care and Use Committee (IACUC) provided guidance on and approved the protocol.*

Note that full information on the approval of the study protocol must also be provided in the manuscript.

Clinical data

Policy information about clinical studies

All manuscripts should comply with the ICMJE guidelines for publication of clinical research and a completed CONSORT checklist must be included with all submissions.

Clinical trial registration	*Provide the trial registration number from ClinicalTrials.gov or an equivalent agency.*
Study protocol	*Note where the full trial protocol can be accessed OR if not available, explain why.*
Data collection	*Describe the settings and locales of data collection, noting the time periods of recruitment and data collection.*
Outcomes	*Describe how you pre-defined primary and secondary outcome measures and how you assessed these measures.*

Dual use research of concern

Policy information about dual use research of concern

Hazards

Could the accidental, deliberate or reckless misuse of agents or technologies generated in the work, or the application of information presented in the manuscript, pose a threat to:

No	Yes	
☒	☐	Public health
☒	☐	National security
☒	☐	Crops and/or livestock
☒	☐	Ecosystems
☒	☐	Any other significant area

Experiments of concern

Does the work involve any of these experiments of concern:

No	Yes	
☒	☐	Demonstrate how to render a vaccine ineffective
☒	☐	Confer resistance to therapeutically useful antibiotics or antiviral agents
☒	☐	Enhance the virulence of a pathogen or render a nonpathogen virulent
☒	☐	Increase transmissibility of a pathogen
☒	☐	Alter the host range of a pathogen
☒	☐	Enable evasion of diagnostic/detection modalities
☒	☐	Enable the weaponization of a biological agent or toxin
☒	☐	Any other potentially harmful combination of experiments and agents

Plants

Seed stocks	*Report on the source of all seed stocks or other plant material used. If applicable, state the seed stock centre and catalogue number. If plant specimens were collected from the field, describe the collection location, date and sampling procedures.*
Novel plant genotypes	*Describe the methods by which all novel plant genotypes were produced. This includes those generated by transgenic approaches, gene editing, chemical/radiation-based mutagenesis and hybridization. For transgenic lines, describe the transformation method, the number of independent lines analyzed and the generation upon which experiments were performed. For gene-edited lines, describe the editor used, the endogenous sequence targeted for editing, the targeting guide RNA sequence (if applicable) and how the editor was applied.*
Authentication	*Describe any authentication procedures for each seed stock used or novel genotype generated. Describe any experiments used to assess the effect of a mutation and, where applicable, how potential secondary effects (e.g. second site T-DNA insertions, mosiacism, off-target gene editing) were examined.*

ChIP-seq

Data deposition

☐ Confirm that both raw and final processed data have been deposited in a public database such as GEO.

☐ Confirm that you have deposited or provided access to graph files (e.g. BED files) for the called peaks.

Data access links<br>*May remain private before publication.*	*For "Initial submission" or "Revised version" documents, provide reviewer access links. For your "Final submission" document, provide a link to the deposited data.*
Files in database submission	*Provide a list of all files available in the database submission.*
Genome browser session<br>(e.g. UCSC)	*Provide a link to an anonymized genome browser session for "Initial submission" and "Revised version" documents only, to enable peer review. Write "no longer applicable" for "Final submission" documents.*

Methodology

Replicates	*Describe the experimental replicates, specifying number, type and replicate agreement.*
Sequencing depth	*Describe the sequencing depth for each experiment, providing the total number of reads, uniquely mapped reads, length of reads and whether they were paired- or single-end.*
Antibodies	*Describe the antibodies used for the ChIP-seq experiments; as applicable, provide supplier name, catalog number, clone name, and lot number.*
Peak calling parameters	*Specify the command line program and parameters used for read mapping and peak calling, including the ChIP, control and index files used.*
Data quality	*Describe the methods used to ensure data quality in full detail, including how many peaks are at FDR 5% and above 5-fold enrichment.*
Software	*Describe the software used to collect and analyze the ChIP-seq data. For custom code that has been deposited into a community repository, provide accession details.*

Flow Cytometry

Plots

Confirm that:

- [ ] The axis labels state the marker and fluorochrome used (e.g. CD4-FITC).
- [ ] The axis scales are clearly visible. Include numbers along axes only for bottom left plot of group (a 'group' is an analysis of identical markers).
- [ ] All plots are contour plots with outliers or pseudocolor plots.
- [ ] A numerical value for number of cells or percentage (with statistics) is provided.

Methodology

Sample preparation	*Describe the sample preparation, detailing the biological source of the cells and any tissue processing steps used.*
Instrument	*Identify the instrument used for data collection, specifying make and model number.*
Software	*Describe the software used to collect and analyze the flow cytometry data. For custom code that has been deposited into a community repository, provide accession details.*
Cell population abundance	*Describe the abundance of the relevant cell populations within post-sort fractions, providing details on the purity of the samples and how it was determined.*
Gating strategy	*Describe the gating strategy used for all relevant experiments, specifying the preliminary FSC/SSC gates of the starting cell population, indicating where boundaries between "positive" and "negative" staining cell populations are defined.*

- [ ] Tick this box to confirm that a figure exemplifying the gating strategy is provided in the Supplementary Information.

Magnetic resonance imaging

Experimental design

Design type	*Indicate task or resting state; event-related or block design.*
Design specifications	*Specify the number of blocks, trials or experimental units per session and/or subject, and specify the length of each trial or block (if trials are blocked) and interval between trials.*
Behavioral performance measures	*State number and/or type of variables recorded (e.g. correct button press, response time) and what statistics were used to establish that the subjects were performing the task as expected (e.g. mean, range, and/or standard deviation across subjects).*

Acquisition

Imaging type(s)	*Specify: functional, structural, diffusion, perfusion.*
Field strength	*Specify in Tesla*
Sequence & imaging parameters	*Specify the pulse sequence type (gradient echo, spin echo, etc.), imaging type (EPI, spiral, etc.), field of view, matrix size, slice thickness, orientation and TE/TR/flip angle.*
Area of acquisition	*State whether a whole brain scan was used OR define the area of acquisition, describing how the region was determined.*

Diffusion MRI ☐ Used ☐ Not used

Preprocessing

Preprocessing software	*Provide detail on software version and revision number and on specific parameters (model/functions, brain extraction, segmentation, smoothing kernel size, etc.).*
Normalization	*If data were normalized/standardized, describe the approach(es): specify linear or non-linear and define image types used for transformation OR indicate that data were not normalized and explain rationale for lack of normalization.*
Normalization template	*Describe the template used for normalization/transformation, specifying subject space or group standardized space (e.g. original Talairach, MNI305, ICBM152) OR indicate that the data were not normalized.*
Noise and artifact removal	*Describe your procedure(s) for artifact and structured noise removal, specifying motion parameters, tissue signals and physiological signals (heart rate, respiration).*

Volume censoring | *Define your software and/or method and criteria for volume censoring, and state the extent of such censoring.*

Statistical modeling & inference

Model type and settings | *Specify type (mass univariate, multivariate, RSA, predictive, etc.) and describe essential details of the model at the first and second levels (e.g. fixed, random or mixed effects; drift or auto-correlation).*

Effect(s) tested | *Define precise effect in terms of the task or stimulus conditions instead of psychological concepts and indicate whether ANOVA or factorial designs were used.*

Specify type of analysis: ☐ Whole brain ☐ ROI-based ☐ Both

Statistic type for inference | *Specify voxel-wise or cluster-wise and report all relevant parameters for cluster-wise methods.*

(See Eklund et al. 2016)

Correction | *Describe the type of correction and how it is obtained for multiple comparisons (e.g. FWE, FDR, permutation or Monte Carlo).*

Models & analysis

n/a	Involved in the study
☐	☐ Functional and/or effective connectivity
☐	☐ Graph analysis
☐	☐ Multivariate modeling or predictive analysis

Functional and/or effective connectivity | *Report the measures of dependence used and the model details (e.g. Pearson correlation, partial correlation, mutual information).*

Graph analysis | *Report the dependent variable and connectivity measure, specifying weighted graph or binarized graph, subject- or group-level, and the global and/or node summaries used (e.g. clustering coefficient, efficiency, etc.).*

Multivariate modeling and predictive analysis | *Specify independent variables, features extraction and dimension reduction, model, training and evaluation metrics.*
